# Transcriptomic Insights into Mechanisms of Early Seed Maturation in the Garden Pea (*Pisum sativum* L.)

**DOI:** 10.3390/cells9030779

**Published:** 2020-03-23

**Authors:** Yury V. Malovichko, Oksana Y. Shtark, Ekaterina N. Vasileva, Anton A. Nizhnikov, Kirill S. Antonets

**Affiliations:** 1 Laboratory for Proteomics of Supra-Organismal Systems, All-Russia Research Institute for Agricultural Microbiology (ARRIAM), Podbelskogo sh., 3, Pushkin, 196608 St. Petersburg, Russia; a.nizhnikov@arriam.ru; 2 Faculty of Biology, St. Petersburg State University, 199034 St. Petersburg, Russia; evasilieva@arriam.ru; 3 Department of Biotechnology, All-Russia Research Institute for Agricultural Microbiology (ARRIAM), Podbelskogo sh., 3, Pushkin, 196608 St. Petersburg, Russia; oshtark@yandex.ru

**Keywords:** garden pea, *Pisum sativum* L., Sprint-2, seed, transcriptome, maturation, early maturation, desiccation, transcription, transposable element

## Abstract

The garden pea (*Pisum sativum* L.) is a legume crop of immense economic value. Extensive breeding has led to the emergence of numerous pea varieties, of which some are distinguished by accelerated development in various stages of ontogenesis. One such trait is rapid seed maturation, which, despite novel insights into the genetic control of seed development in legumes, remains poorly studied. This article presents an attempt to dissect mechanisms of early maturation in the pea line Sprint-2 by means of whole transcriptome RNA sequencing in two developmental stages. By using a de novo assembly approach, we have obtained a reference transcriptome of 25,756 non-redundant entries expressed in pea seeds at either 10 or 20 days after pollination. Differential expression in Sprint-2 seeds has affected 13,056 transcripts. A comparison of the two pea lines with a common maturation rate demonstrates that while at 10 days after pollination, Sprint-2 seeds show development retardation linked to intensive photosynthesis, morphogenesis, and cell division, and those at 20 days show a rapid onset of desiccation marked by the cessation of translation and cell anabolism and accumulation of dehydration-protective and -storage moieties. Further inspection of certain transcript functional categories, including the chromatin constituent, transcription regulation, protein turnover, and hormonal regulation, has revealed transcriptomic trends unique to specific stages and cultivars. Among other remarkable features, Sprint-2 demonstrated an enhanced expression of transposable element-associated open reading frames and an altered expression of major maturation regulators and DNA methyltransferase genes. To the best of our knowledge, this is the first comparative transcriptomic study in which the issue of the seed maturation rate is addressed.

## 1. Introduction

Members of the Fabaceae family, colloquially referred to as legumes, are of great interest to agriculture because of their high seed protein content. Of these, the garden pea (*Pisum sativum* L.) is presumably one of the most important crop cultures grown ubiquitously for both human and animal feeding [1]. Despite the economic value of pea seeds, genetic programs underlying seed maturation and vigor have not been completely revealed. Until recently [2], the situation has been further confounded with the lack of a pea genome sequence, making the pea one of the most prominent ‘genomic orphans’ [3]. At the same time, extensive genetic studies might shed light on key agricultural properties of pea seeds, including the accumulation of nutrients, storage longevity, and maturation rate.

Legume seeds’ development process is conditionally divided into three stages, referred to as pre-maturation, maturation, and desiccation, respectively [4]. The presence of a desiccation stage indicates that legume seeds belong to the so-called orthodox type, opposed to recalcitrant seeds, which skip the desiccation and dormancy phases and proceed to germination once the maturation is over [5]. Though the exact timing and duration of these stages may vary depending on the species, cultivar, and environmental conditions, they exist in all legume species and reflect the genetic and cellular events occurring in seed tissues. In the first stage, which is predominantly controlled by maternal signals, embryo growth and morphogenesis are sustained by active cell division [6,7]. This phase differs from the pre-maturation stage in both the switching to filial development control and the transition from cell division to cell expansion growth. Another feature of the middle development stage is the intense accumulation of nutrient entities, including carbohydrates, fatty acids, and storage proteins [8,9,10,11]. These processes are governed by four main regulators: ABSCISIC ACID INSENSITIVE3 (ABI3), FUSCA3 (FUS3), LEAFY COTYLEDON2 (LEC2) and LEC1, with the former three being B3-containing transcription activators and commonly referred to as AFL (ABI3, FUS3, LEC2) [12]. Finally, the desiccation stage denotes the cessation of seed growth and intense metabolic activity. Seeds diminish in linear sizes and undergo dehydration, which triggers the expression of stress-related genes, including DNA reparation enzymes [13], ROS scavengers [14], and protein unfolding and aggregation inhibitors, such as LATE EMBRYOGENESIS ABUNDANT (LEA) proteins [15]. Once having passed all these stages, the seed proceeds to a dormant state until germination. Transitions between these stages are governed by signals of both filial and maternal origin, including abscisic acid (ABA), gibberelin (GA), and seed sugar composition [4].

Though this scheme encompasses the main idea of seed development, the exact timing of the described stages varies immensely among flowering plants. Two distinct mechanisms, namely accelerated rates of seed development (precocious maturation) and the early germination of mature plants (precocious germination), may result in the shortening of these timings. A vivid example of precocious germination is the aforementioned recalcitrant seeds found in plants dwelling in humid environments, including some tropical legumes [5]. In its extreme variant, also called viviparity, precociously germinating seeds sprout while still residing in maternal fruits [16]. In plants bearing orthodox seeds, viviparity is found in desiccation-intolerant mutants [17,18,19]. Research on both viviparous mutants and natural producers of recalcitrant seeds indicates that premature germination occurs due to the distortion of ABA signal pathways by the repression of ABA biosynthesis [20,21] or mutations in genes encoding downstream transcription factors [17]. Compared to this, precocious, or early, maturation mechanisms remain unclear. It is possible that early seed maturation may be concordant with an early vegetative maturity trait, which has been studied in the pea and soybean and is thought to be under the control of numerous quantitative trait loci (QTL) [22,23]. Moreover, the induction of early maturation can be achieved by drying, as has been shown in soybean [24,25].

To date, studies dedicated to the transcriptomic or proteomic profiling of developing seeds have been performed for several legume species, including the chickpea [26], soybean [27,28], barrel medic [29,30,31], common bean [13,32], and garden pea [33,34,35]. These large-scale assays allow global genetic programs involved in seed maturation to be dissected, and expression changes to be traced over time. However, none of the studies have addressed the problem of seed maturation precocity in legumes. To solve this, we performed full transcriptome sequencing (RNA-Seq) of seeds belonging to the pea genetic line Sprint-2. These plants demonstrate early vegetative maturation and are therefore widely used in studies on normal physiology and symbiosis establishment in peas [36]. For this line, rapid seed maturation has been previously claimed, but not properly dissected; we have assumed, however, that early seed maturation could accompany the early maturation of germinated plants. The dissection of transcriptomic dynamics in Sprint-2 is coupled here with a comparison to the open access data for pea lines with an average maturation rate. A particular importance of various transcription regulators and dynamics of maturation-associated cell activities, such as storage protein synthesis, the stress response, and carbohydrate metabolism, was observed during differential expression analysis as a prominent source of potent early maturation markers.

## 2. Materials and Methods

### 2.1. Plant Material

The *Pisum sativum* L. genetic line Sprint-2 from the collection of the All-Russia Research Institute for Agricultural Microbiology (ARRIAM, Saint Petersburg, Russia) was used in this study. The line was initially obtained as a result of crossing cv. Avanti (Siberian Research Institute of Plant Cultivation and Breeding, Krasnoobsk, Novosibirsk region, Russia) with genotypes K-7036 and K-419 (N.I. Vavilov All-Russian Institute for Plant Genetic Resources, Saint Petersburg, Russia) and subsequent selection for precocity and survival in a growth chamber to serve as an object of genetic laboratory studies [37]. The line is characterized by a relatively short stem (30–50 cm), early flowering start (24–28 days from planting dry seed), early maturity (50–55 days from planting dry seed to harvesting the first mature dry seed), and determinate growth (no more than five pods per plant). The line carries the following genetic markers: white flowers (*a*), absence of an anthocyanin ring in the leaf axil (*d*), anthocyanin spots on the seed coat (*Fs*), yellow cotyledons (*I*), marbled seed coat (*M*), and round cotyledons *(R*) [37]. Additionally, Sprint-2 has a normal karyotype and axial location of flowers.

Plants were grown in pots with ‘Terra vita Universal’ peat soil (MNPP FART Ltd., St. Petersburg, Russia), which had the following characteristics: pH (KCl) 6.0; 150 mg/L available nitrogen (NH_4_^+^ + NO_3_^−^); 270 mg/L available phosphorous (P_2_O_5_); and 300 mg/L available potassium (K_2_O). The following climatic conditions were maintained by using the Heraeus Voetch (Lindenstruth, Germany) growth chamber: day/night 16/8 h; 21/19 °C; relative humidity 75%; and illumination 10,000 lux.

**Figure 1:**
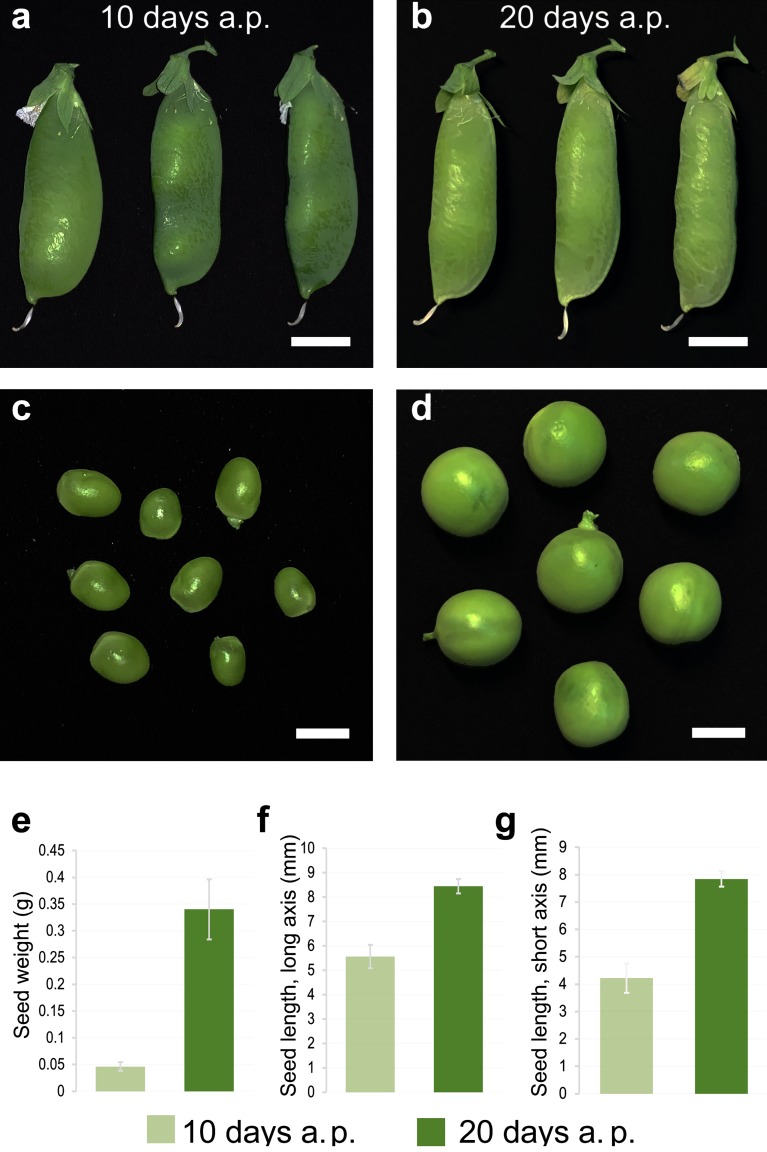
Morphometry of the *Pisum sativum* L. genetic line Sprint-2 seeds collected at 10 and 20 days after pollination (a.p.). (**a**,**b**) Pods are shown, scale bars are equal to 10 mm. (**c**,**d**) Seeds are shown, scale bars are equal to 5 mm. Average seed weights (**e**), where lengths along the long (**f**) and short (**g**) axes are shown. Data are presented as the means ± the standard deviations.

The pollination event was attributed to the date of full flower opening. Seeds were collected at 10 (flat pod) and 20 (pod fill) days after pollination (DAP) (according to Knott [38], with slight modifications), which stand for the pre-maturation and maturation stages of normal pea seed development, respectively (Figure 1). Collected seeds were immediately frozen in liquid nitrogen and then stored at –80 °C. Sample randomization was achieved by mixing seeds collected at the same stage at the end of each harvest period.

### 2.2. RNA Extraction, Library Preparation, and Whole Transcriptome RNA Sequencing

Four random samples from each of the seed pools, either 10 or 20 DAP, were chosen for RNA extraction and RNA-Seq library preparation. RNA was extracted with NucleoSpin RNA plant tissue (MACHEREY-NAGEL GmbH & Co. KG, Düren, Germany), according to the manufacturer’s protocol. One sample from each pool was then used for the preparation of a stranded library with increased coverage with the TruSeq Stranded Total RNA Library Prep Kit (Illumina, San Diego, CA, USA), and the other six samples were prepared with the TruSeq RNA Library Prep Kit v2 (Illumina).

The whole transcriptome RNA sequencing was performed with Illumina HiSeq 4000 (Illumina) in paired-end mode, with a read length of 2 × 100 bp, by Macrogen Inc. (Seoul, South Korea). Quality control was executed with HiSeq Control Software v3.3 and Real Time Analysis v2.7.3. Base calling was performed with the Illumina bcl2fastq (v2.17.1.14) package.

### 2.3. RNA-Seq Read Processing and De Novo Transcriptome Assembly

Raw reads were trimmed and quality-filtered using the *bbduk.sh* script from the BBMap package [39] v38.16. To ensure that neither adapter sequences nor spurious reads would pass quality control, quality-trimming was performed on both read ends with an average quality equal to 20, k-mer size equal to 23, and minimal read length equal to 50. Indication on library statistics before and after trimming is given in Table S1.

Libraries with an increased coverage and stranded layout were corrected by using Rcorrector [40] v1.0.4 with default settings and further used for de novo assembly with Trinity v2.8.4 [41] with default settings. 10 DAP- and 20 DAP-corrected stranded libraries were pooled together to increase the total coverage. The quality of the resulting assembly was assessed by aligning respective stranded libraries back to assemblies with bowtie2 v.2.3.5 [42], aligning assemblies against the Viridiplantae IPG database with BLAST [43], and testing for the benchmarking universal single-copy ortholog (BUSCO) content [44]. Assembly statistics were obtained with *TrinityStats.pl* support scripts from the Trinity package. Putative coding regions were extracted with TransDecoder [45] with a minimal length cutoff equal to 100 amino acids. Deduplication of joint assembly was based on the pipeline suggested by Alves-Carvalho et al. [46]. In brief, the assembly was filtered of contigs not containing open reading frames (ORFs), contigs less than 300 nt long, and contigs covered by respective ORFs by less than 30% with a custom Python script. Next, proteins encoded in extracted ORFs were clustered with CD-Hit [47] with an identity cutoff value equal to 0.9 to eradicate duplicates. Resulting transcriptome assembly was subject to quality and representation assessment, as described for initial assemblies. Finally, contigs were mapped to a recently published genome assembly of the ‘Caméor’ pea cultivar available at https://urgi.versailles.inra.fr/download/pea/ using minimap2 [48] v2.17-r941 in *splice* mode with a transcript strand-restricted search of splicing sites. A detailed quality assessment was obtained with rnaQUAST [49] v1.6.0.

### 2.4. Transcriptome Assembly Annotation

Most of the annotation was deduced with the Trinotate [50] pipeline, which encompasses several tools, including BLAST, HMMer 3 [51], TmHMM [52], and signalP [53]. BLASTP and BLASTX were run with the Viridiplantae-specific IPG database (6,959,975 entries total) with an E-value cutoff equal to 1 × 10^−5^. The rest of the Trinotate-incorporated tools were launched with default parameters. MapMan annotation was performed independently by using the Mercator server [54], with *Arabidopsis*, *Oryza*, and *Chlamydomonas* databases as targets and blast cutoff of 80% of identity. KEGG terms were also deduced independently from Trinotate with the BLASTKoala server [55].

To increase the robustness of GeneOntology (GO) over-representation tests, the following steps were taken. First, non-redundant Pfam GO annotations were added to BLAST-deduced GO annotations. After that, eggNOG v4.5 [56] in *diamond* mode was launched against a protein FASTA file and resulting GO annotations were extracted. Finally, MapMan categories were translated into GO terms with a custom script and BIN2GO cross-mapping obtained from the MapMan official website.

### 2.5. Incorporation of Open Access Transcriptomic Data into Analysis

Comparative transcriptomic analysis was performed using transcriptomic data for cultivars Zhewan-1 and Zhongwan-6 provided by Liu et al. [33]. Zhewan-1 is a typical vegetable pea cultivar, i.e., it has a higher seed sugar content than Zhongwan-6, which is attributed to the so-called grain cultivars. Both Zhewan-1 and Zhongwan-6 seeds reach physiological maturity at 30 DAP. Transcriptome libraries of Zhewan-1 and Zhongwan-6 cultivar seed at 10 and 25 DAP were downloaded from NCBI SRA (accession SRP063052) using SRA Toolkit [57] v2.9.6 utilities. All four conditions were represented by a single paired-end cDNA library, and were trimmed with the *bbduk.sh* script in the same way as Sprint-2 libraries; the distribution of read numbers before and after trimming is indicated in Table S1. Since each condition was presented by a single library, artificial technical repeats were generated by subsampling each library with Seqtk software [58] into four paired-end pseudoreplicates comprising 75% randomly chosen pairs from the source library. We also note that pea material in the original studies was grown in uncontrolled conditions, which, together with the limited number of biological replicates, has a negative impact on the analysis credibility.

Transcriptome assembly from the original study was obtained from the NCBI GEO database (accession GSE72573, supplementary material). The assembly was subject to the filtering and annotation pipeline, as described above. Contigs from the filtered assembly which failed to align to the Sprint-2 transcriptome assembly in the minimap2 *assembly* mode were added to the Sprint-2 assembly to form a joint transcriptome for interlinear differential expression (DE) estimation. To avoid duplicates, the joint assembly was also deduplicated by running CD-Hit against respective protein sequences with an identity cutoff value equal to 0.9; Sprint-2-derived contigs were used as cluster representatives where possible to increase the assembly fidelity.

### 2.6. Read Pseudoalignment and Estimation of Differential Expression

Kallisto [59] v.0.44.0 was used to pseudoalign all acquired RNA-Seq libraries against pantranscriptome assembly with a bootstrap number equal to 150. For Sprint-2 intralinear DE, estimation reads were aligned to the initial Sprint-2 assembly, while interlinear changes were estimated using the joint assembly. Resulting outputs were further assessed for differential expression with the sleuth [60] R package, v0.30.0. Transcripts were treated as differentially expressed if they passed both the likelihood ratio test and Wald test at a q-value cutoff value equal to 0.001 and lnFC (sleuth’s ‘b’ parameter) modulus cutoff value equal to 0.5.

### 2.7. GO Over-Representation Tests

GO over-representation was performed with the topGO R package [61], v.2.34.0. The Fisher hypergeometric criterion and ‘weight01’ graph reduction algorithm were used to infer over-represented terms with a false discovery rate (FDR)-adjusted p-value cutoff value equal to 0.01. Dubious terms not related to plant physiology were removed from the results though parsed and analyzed independently. The results were visualized via ggplot2 [62] and RcolorBrewer [63] v1.1-2 R packages.

### 2.8. Sample Clustering

Three different approaches were implemented to test the differences in maturation dynamics between Sprint-2, Zhewan-1, and Zhongwan-6. First, transcript per million (TPM) metrics from each alignment were extracted and combined into a single matrix. These data were further used to perform PCA dimension reduction using the ggbiplot [64] v0.55 R package. For the sake of consistency, pairwise combinations of the first five principal components were used in PCA plots. Eigenvector summands for each PC were extracted with the respective BLAST annotations and ranged in the decreasing order of their multipliers. Another approach included finding distances between TPM vectors with Poissonian model clustering [65], as implemented in the PoiClaClu v1.0.2.1 R package and subsequent result plotting on a heatmap generated with the Pheatmap [66] v1.0.12 package. Several iterations of distance plotting were performed to test the impact of different transcript functional groups on sample similarity. Finally, cosine similarity metrics were calculated for Sprint-2 stages and cultivars in comparable maturation stages. For this, a ternary vector indicating whether a transcript was upregulated (1), downregulated (-1), or not affected by significant transcription changes (0) was allocated to each comparison. Cosine similarity was then calculated for each pair of vectors using the cosine function from the lsa [67] v0.73.1 R package.

### 2.9. Term-Wise Transcript Clustering

Common transcription trends between cultivars and time points were performed with self-organizing maps implemented in the oposSOM [68] R package, v2.4.0. Map sizes were selected according to the developers’ recommendations on the dataset size. Mapping was performed first for the total transcriptome and then for specific functional groups formed according to MapMan annotations. Transcripts allocated to group overexpression spots were tested for the over-representation of GO terms, as described above. Visualization of the transcriptomic landscape was based on the oposSOM group overexpression reports recolored in Adobe Photoshop v.CS5.

All transcripts were allocated coordinates denoting the mean TPM values at earlier (10 DAP) and later (20 or 25 DAP) stages of seed development in the respective cultivars and then plotted using the ggplot2 package. To achieve the desired analysis consistency, transcripts were mapped according to their belonging to one of the selected functional categories composed of related GO and MapMan terms. A full list of terms used for functional category composition can be found in the companion RMarkdown file. Outliers were identified using Cook’s distance method [69], as implemented in the stats R package [70] v3.6.1, with cut-off equal to four-fold the mean Cook’s distance from the linear regression dependency. Individual transcripts posing specific interest based on transcript clustering or literature material were found by performing BLAST against the set of reference entries obtained from NCBI RefSeq. If more than one isoform was detected in the assembly, all the isoforms were further plotted, with arbitrary numeric identifiers added. Absent transcripts indicate the loss of data during sleuth variance shrinkage.

### 2.10. Variant Calling and Single Nucleotide Polymorphism (SNP) Annotation

Variant calling of two pea lines against Sprint-2 initial assembly was performed with the *run_variant_calling.py* script from the Trinity toolkit. The pipeline wrapped with the script was comprised of STAR [71] v. 2.7.1a, PicardTools [72] v2.12.1, and Genome Analysis Toolkit (GATK) [73] v3.8. Due to the presence of alternative spliceforms, which could cause ambiguous read mapping, the transcripts were first fetched to a supertranscript level with another Trinity toolkit script, Trinity_gene_splice_modeler.py, and the read files were merged cultivar-wise. The pipeline raw output was then cleaned of indels and hard-filtered with GATK 4.1.2 VariantFiltration utility with the following settings: FS > 30, QD < 2, DP < 15, QUAL < 30, MQ < 40, SOR > 3, MQRankSum −12, and ReadPosRankSum < −8. SNPs, having passed filtering, were transferred from genes to transcripts by shifting their coordinates with a custom Python script. For genes encoding more than one spliceform, SNPs were attributed to each of the transcripts encoded.

For the annotation of filtered and corrected variants, an snpEff [74] v4.3t reference base was created by using the Sprint-2 transcriptome sequences and respective gene feature layout. Annotated vcf files were then divided into homozygous and heterozygous entries based on the GATK for independent downstream treatment. For the Sprint-2 cultivar, homozygous polymorphic sites were discarded as false positives of either assembly or STAR alignment, as suggested elsewhere [75]. Variants with erroneous snpEff labels were also discarded for all three cultivars. Gene annotation and over-representation analysis for SNP subsets were performed as described above for differentially expressed genes. The SNP distribution was visualized with the UpSetR R package [76].

## 3. Results

### 3.1. RNA-Seq De Novo Assembly and Annotation of Pea Seed Transcriptomes

As a result of RNA-Seq, a total of 1,949,126,068 reads were obtained (Table S1). Due to the sound volume of the initial library, strict quality trimming was applied, resulting in the loss of approximately 20% of reads. Untrimmed libraries are available at NCBI SRA under the accession numbers SRX6816033-SRX6816040.

**Figure 2:**
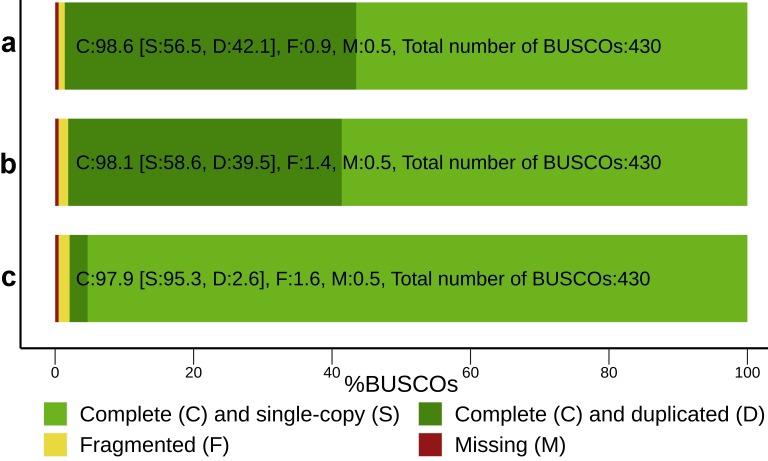
Assessment of BUSCO content improvement during transcriptome assembly preparation. (**a**) The initial Trinity assembly statistics. (**b**) A filtered assembly containing duplicates. (**c**) A final deduplicated assembly.

Because of the persistent ‘genomic orphan’ status of the pea, de novo assembly with Trinity was performed using stranded and k-mer-corrected libraries. Subsequent filtering and deduplication of the produced assembly resulted in an increase of assembly statistics (Table 1 and Table S2) and BUSCO assessment results (Figure 2). Final assembly comprised 25,756 transcripts (contigs) distributed between 2112 genes (supertranscripts). The assembly is available at NCBI Transcriptome Shotgun Assembly (TSA) under accession number GHVW00000000.2.

A total of 24,244 and 24,463 transcripts were identified with BLASTP and BLASTX, respectively, against a Viridiplantae IPG database, while 1192 transcripts lacked any BLAST annotation (Table S3). Most of these orphan transcripts also lacked a domain layout according to the Pfam, TmHMM, and signalP results, as well as functional annotation form GO and MapMan ontologies, but were still preserved in the assembly. In total, 19,862 transcripts were assigned at least one GO term and 16,730 had a MapMan signature other than ‘not assigned.unknown’. Additionally, 600 terms were annotated as involved in transposable element (TE) mobility and integration; of these, more than 500 represent actual transposon-nested open reading frames from both DNA and RNA TEs.

To ensure that the assembly represented actual coding sequences of the pea genome, we mapped the whole transcriptome to the novel pea genome assembly performed by Kreplak et al. [2] using *minimap2* in splice mode. It was revealed that 25,732 of 25,756 contigs aligned to the genome assembly precisely one time, with no multi-mapped contigs present. Of the 24 unmapped transcripts, 14 contigs missed any annotation entirely, indicating either misassembly events or transcripts encoding novel proteins, and were preserved in the final assembly for the sake of consistency.

### 3.2. Transcriptomic Markers of Maturity during the Transition from Pre-Maturation (10 DAP) to Seed Filling (20 DAP)

**Figure 3:**
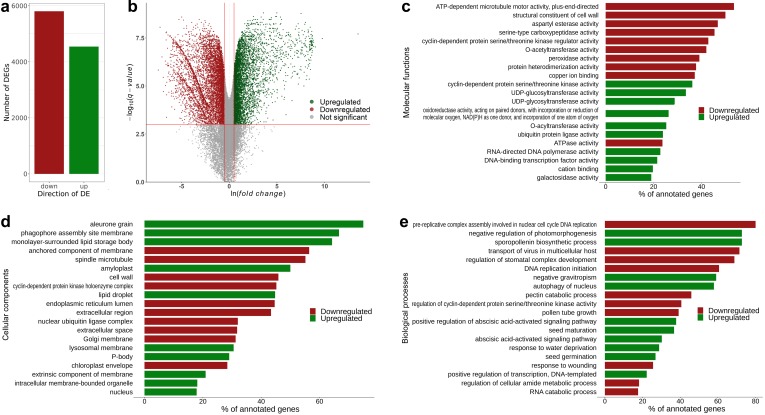
Transcriptomic changes in the transition from seed pre-maturation to seed filling stages in Sprint-2. (**a**) A total number of differentially expressed transcripts at 20 DAP compared to 10 DAP seeds. (**b**) Volcano plots demonstrating differentially expressed transcripts in 20 DAP seeds compared to 10 days after pollination (DAP) seeds. Plotted on the *x*-axis is the ln fold difference between 10 DAP and 20 DAP seeds. Plotted on the *y*-axis is the −log_10_(q-value). Genes whose expression changes are nonsignificant are shown in gray below the red line, while significant differentially expressed genes (FDR < 0.001) are divided into upregulated (green dots) and downregulated (red dots) genes. (**c**–**e**) Visualization of terms over-represented among differentially expressed genes from Biological Process, Cellular Component, and Molecular Function aspects, respectively. The first ten categories have been chosen per aspect and arranged by the adjusted q-value. Bars are ranked according to the number of transcripts from the respective category being differentially expressed.

We first assessed the differential expression occurring upon the transition from 10 to 20 DAP in Sprint-2. In most of the pea lines, these time points stand for pre-maturation and midmaturation, respectively; however, Sprint-2 seeds at 20 DAP show phenotypic traits intrinsic for either late maturation or desiccation onset. In total, 5805 transcripts were downregulated and 4543 transcripts were upregulated in Sprint-2 (Figure 3a,b, Tables S4 and S5a,b). The highest transcript numbers and log-fold changes (indicated by the ‘b’ parameter in the sleuth output) were registered for transcripts encoding storage proteins such as vicilin, convicilin, and legumin. Corresponding gene subsets were then tested for GO term over-representation using topGO (Figure 3b–d, Table S5c–h). Since the plants were grown in fully controlled conditions, we assumed that the observed differences reflect developmental changes solely, with minimal environmental influence.

Most of the observed changes manifested in the GO terms are consistent with the common logic of seed maturation (Table S5c–h). These include the (a) cessation of cell division and DNA replication in favor of expansion growth, (b) accumulation of storage moieties, and (c) enhancement of abscisic acid synthesis and respective signal transduction mechanisms. While terms related to DNA synthesis were concordantly repressed at 20 DAP, terms for RNA biogenesis showed more complicated patterns. Transcription-related terms were both up- and downregulated, categories for splicing and RNA deposition were upregulated, and RNA catabolic processes were negatively modulated. Other downregulated categories included nucleotide synthesis, cytosine methylation, and nucleosome modification. Protein turnover was prominently shifted to proteasomal degradation, as indicated by all three Gene Ontology aspects. In contrast to proteasome-dedicated degradation, terms related to serine- and threonine-targeted catabolic proteolysis showed significant downregulation in the Biological Process and Molecular Function aspects. Cell wall synthesis, endosomal transport, the tubulin cytoskeleton constituent, and microtubule-associated intracellular mobility were also alleviated in the 20 DAP seeds; these trends, however, might correspond to each other in the context of cell wall biogenesis. Numerous terms from Biological Process and Cellular Components also refer to cell autophagy, particularly to nucleus and mitochondrion digestion.

Most of the carbohydrate metabolism transcripts were repressed in the maturing seeds. In contrast to storage protein and oil accumulation, starch biosynthesis was not notably promoted at 20 DAP judging by the GO over-representation test, except for the ‘amyloplast’ Cellular Component term. Transcripts encoding the main enzymes of the pathway were selected manually according to BLAST annotation. Of the eight transcripts encoding starch synthases, five were upregulated and three were downregulated at 20 DAP; for four ADP-glucose pyrophosphorylase transcripts, three were upregulated, and only the starch branching enzyme sole transcript was upregulated. At the same time, sucrose synthesis was mostly downregulated, with one upregulated isoform of sucrose synthase against six downregulated ones. This allows us to suggest that at 20 DAP, starch biosynthesis is also promoted compared to the earlier time point. It is notable, however, that at 10 DAP, the TPM metrics for most of the found transcripts were prominently greater than zero, indicating that the onset of starch biosynthesis occurs at or before 10 DAP.

The yellowish pod color and increased tissue density observed at 20 DAP might indicate transition to the desiccation stage. This idea is supported by the upregulation of numerous terms related to desiccation, water deprivation, and response to oxidative stress, mostly from the Biological Process aspect. Most of the transcripts mapped to the respective terms stand for E3 ubiquitin ligases, aquaporins, components of lipid droplets, and dehydrin-like and storage proteins. Since no external water deprivation took place during plant growth, we assumed that plants had started preparing for the desiccation onset by 20 DAP, though a complete transition to desiccation could not be stated in this case.

In brief, most of the observed changes are quite consistent with pre-existing studies. However, several hallmark features, such as the cessation of cell proliferation, promoted autophagy, and boosted response to reactive oxygen species, hinder any definite categorization of the studied developmental stages. Together with the seed morphometric rates, it suggests that at 10 DAP, Sprint-2 enters the seed filling stage and at 20 DAP, it either resides in the late maturation period or starts to enter the desiccation stage.

### 3.3. Sprint-2 Shows Significant Gene Expression Differences to Two Cultivars with Common Development Rates in Both Pre-Maturation and Maturation Stages

To test whether a maturation boost takes place in Sprint-2 seeds, transcriptomic data on two other pea cultivars, namely Zhewan-1 and Zhongwan-6, from pre-existing research [33], were included in the analysis. These two cultivars mostly differ in sugar accumulation and, apparently, protein biosynthesis regulation. Late maturation in both Zhewan-1 and Zhongwan-6 occurs at 30 DAP. At the corresponding time points, Sprint-2 seeds have a greater fresh weight than both Zhewan-1 and Zhongwan-6 (see Figure 1 here and Figure 1 in the original paper). Both cultivars are represented by two RNA-Seq libraries corresponding to either 10 DAP or 25 DAP seeds (Figure 3a,b). As in both Zhewan-1 and Zhongwan-6, the middle stage, or seed maturation, spans from 15 to 25 days, we have suggested that comparisons of 20 DAP seeds of Sprint-2 and 25 DAP seeds of these two cultivars are plausible. The pan-transcriptome for Zhewan-1 and Zhongwan-6 was merged with the Sprint-2 assembly and further deduplicated to form a combined assembly suitable for DE estimation between pea cultivars (Tables S4 and S6). The combination of two transcriptome assemblies resulted in an increase of core statistics, such as N50 counts and the median contig length, and the amelioration of BUSCO statistics (Table S2). Alignment of Sprint-2 reads showed little to no changes in comparison to the results for the initial assembly (Table S7, Figure S1), and the 10 to 25 DAP differential expression results for Zhewan-1 and Zhongwan-6 were more or less consistent with those from the original study based on the GO over-representation tests (Table S8, Figure S2). Because of the uncontrolled growth condition in the original study, terms related to environmental stresses were excluded from further analysis [33].

**Figure 4:**
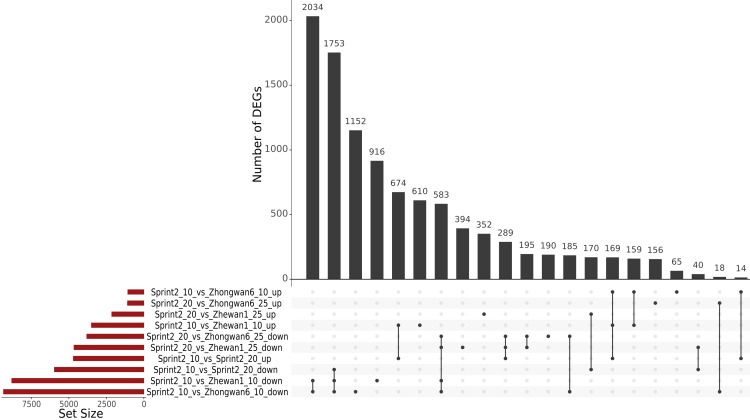
Numbers of differentially expressed genes (DEGs) from all the comparisons involving a combined assembly. In each pair, the latter condition indicates the predictor in the respective comparisons. Plotted are unique DEGs and the most notable intersections; for the full version, see Figure S3.

**Figure 5:**
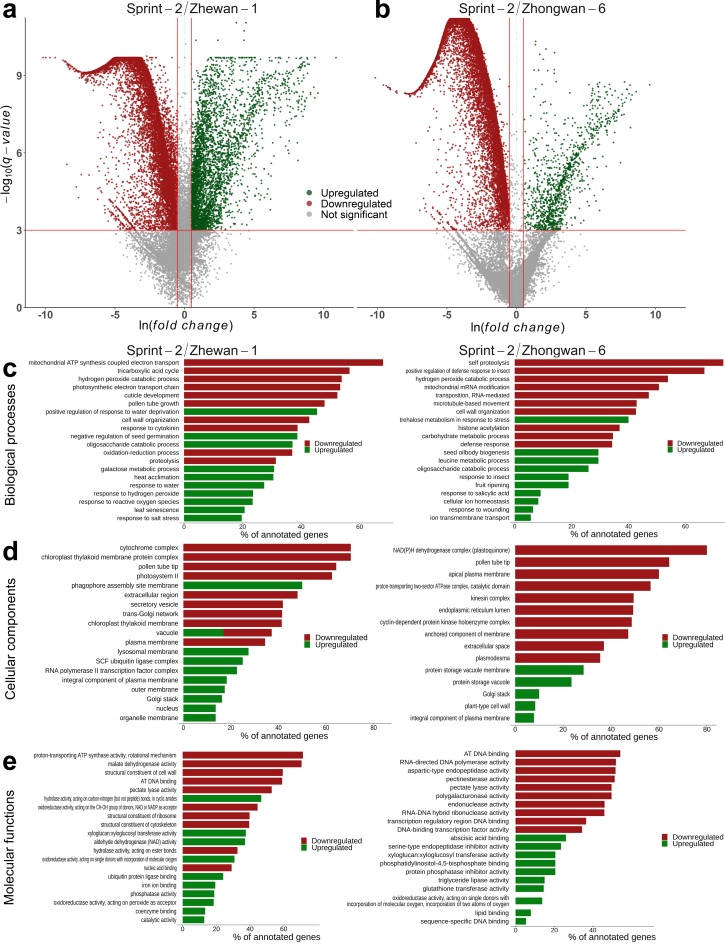
Differential expression in 10 DAP seeds of Zhewan-1 and Zhongwan-6 pea cultivars compared to Sprint-2. (**a**,**b**) Volcano plots demonstrating differentially expressed transcripts in Zhewan-1 and Zhongwan-6, respectively, compared to Sprint-2. Plotted on the *x*-axis is the ln fold difference between Sprint-2 and the corresponding cultivar. Plotted on the *y*-axis is the −log_10_(q-value). Genes whose expression changes are nonsignificant are shown in gray below the red line, while significant differentially expressed genes (FDR < 0.001) are divided into upregulated (green dots) and downregulated (red dots) genes. (**c**–**e**) Visualization of terms over-represented among differentially expressed genes from Biological Process, Cellular Component, and Molecular Function aspects, respectively. The first ten categories have been chosen per aspect and arranged by the adjusted q-value. Bars are ranked according to the number of transcripts from the respective category being differentially expressed.

Though Zhewan-1 and Zhongwan-6 have been shown to differ significantly, they shared a common DE pattern compared to Sprint-2 (Figure 4 and Figure 5, Table S9). In both cases, the number of downregulated genes compared to Sprint-2 exceeded that of upregulated ones. Most of the downregulated transcripts (5999 in total) were concordantly repressed in both cultivars, of which 1753 were also downregulated in Sprint-2 at 20 DAP and 2034 were not regulated in any other comparison (Figure 4). As a result, downregulated transcripts exhibited an over-representation of common terms related to photosynthesis, catabolic energy metabolism, vesicular transport, cell wall biogenesis, and the tubulin cytoskeleton (Table S9). The latter two, judging by the results obtained for Sprint-2, might be indicators of seed maturation. The enrichment of transcripts commonly downregulated in Zhewan-1 and Zhongwan-6 showed an unusual shift towards terms related to transposon activity but was otherwise consistent with the results obtained for full downregulated sets (Table S10, Figure S4). Transcripts downregulated in both comparisons, as well as in 20 DAP Sprint-2 seeds, did not share this feature and comprised terms mostly focused on seed development, carbohydrate metabolism, and cell wall biogenesis (Figure S5). The majority of upregulated transcripts were also represented by shared items (296 for Zhewan-1 and Zhongwan-6 exclusively and 616 shared with Sprint-2 20 DAP seeds), which were enriched in terms related to lipid storage and the oxidative stress response (Table S11, Figure S6). Taken together, the seeds of Zhewan-1 and Zhongwan-6 demonstrated more features of maturation at 10 DAP than those of Sprint-2.

**Figure 6:**
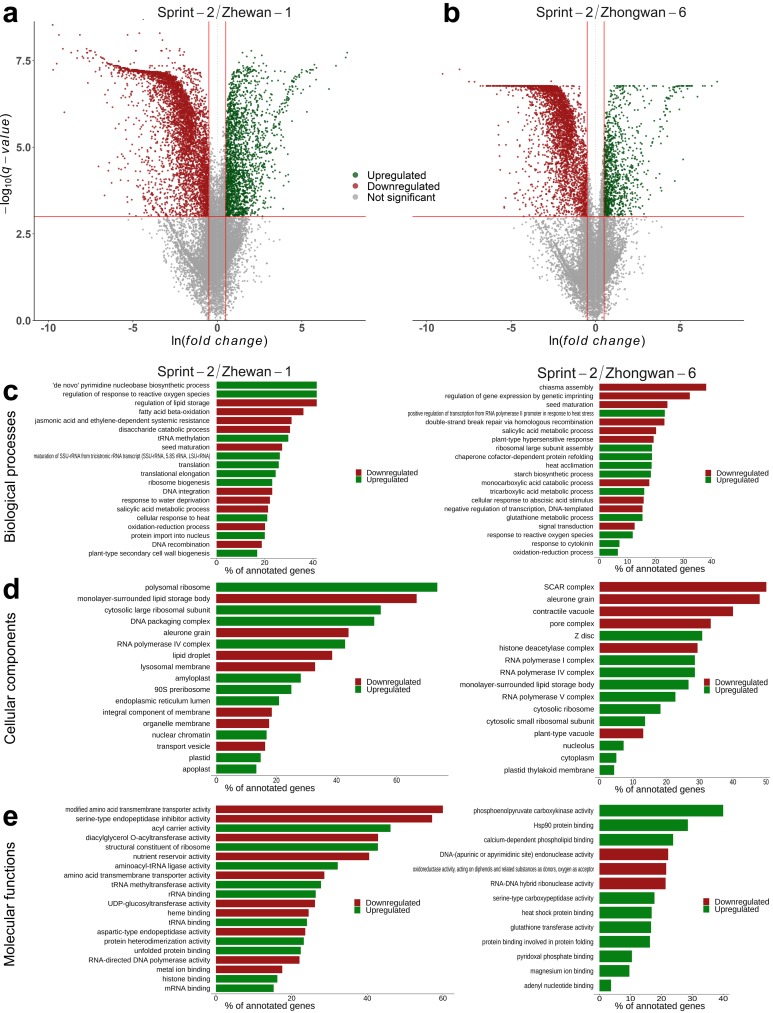
Differential expression in 20 DAP seeds of Zhewan-1 and Zhongwan-6 pea cultivars compared to Sprint-2. (**a**,**b**) Volcano plots demonstrating differentially expressed transcripts in Zhewan-1 and Zhongwan-6, respectively, compared to Sprint-2. Plotted on the *x*-axis is the ln fold difference between Sprint-2 and the corresponding cultivar. Plotted on the *y*-axis is the −log_10_(q-value). Genes whose expression changes are nonsignificant are shown in gray below the red line, while significant differentially expressed genes (FDR < 0.001) are divided into upregulated (green dots) and downregulated (red dots) genes. (**c**–**e**) Visualization of terms over-represented among differentially expressed genes from Biological Process, Cellular Component, and Molecular Function aspects, respectively. The first ten categories have been chosen per aspect and arranged by the adjusted q-value. Bars are ranked according to the number of transcripts from the respective category being differentially expressed.

At the apparent maturation stage (20 or 25 DAP), the situation was the opposite (Figure 6, Table S12). First, the ratio of upregulated to downregulated transcripts compared to Sprint-2 increased in both Zhewan-1 and Zhongwan-6. Upregulated transcripts included numerous terms related to translation, ribonucleoprotein transport, and unfolded protein maintenance. Provided that Zhewan-1 has been shown to surpass Zhongwan-6 in the expression rates of translation-related transcripts, such concordancy indicates a principal difference between Sprint-2 and these two lines. These terms also emerged in over-representation tests for 578 transcripts concordantly upregulated in Zhewan-1 and Zhongwan-6 (Figure S7, Table S13). Processes related to transcription and DNA replication also seemed to be downregulated in Sprint-2, as well as terms from the Cellular Component aspect referring to the chloroplast location. While promoted terms in Zhewan-1 centered around these two trends, those in Zhongwan-6 were more diverse and included the biosynthesis of amino acids, nucleotide bases, and other nitrogen-containing compounds. At the same time, terms upregulated in Sprint-2 mostly referred to the previously defined maturation and/or desiccation markers. These were the (a) response to water deprivation and oxidative stress; (b) promotion of abscisic acid signaling; and (c) accumulation of storage moieties except for starch, which was promoted in Sprint-2. A total of 2301 transcripts concordantly downregulated in Zhewan-1 and Zhongwan-6 confined terms for transposition, desiccation, and oxidative stress (Figure S8, Table S13). Combined with transcription, translation, and replication arrest and DNA repair term promotion, it can be suggested that Sprint-2 is much closer to desiccation and, presumably, dormancy, than Zhewan-1 and Zhongwan-6.

A particular feature of transcripts downregulated in Zhongwan-6 and Zhewan-1 is the over-representation of several terms related to transposon integration and transposition (Tables S9, S10, and S12). A manual search indicated that 172 and 211 transcripts annotated with at least one of the transposon-related GO terms were downregulated in Zhewan-1 and Zhongwan-6 at 25 DAP, respectively. Most of these transcripts had a consistent BLAST annotation referring to TE-nested reading frames. These findings were further corroborated by the upregulation of terms referring to DNA methylation, chromatin silencing, and plant-specific R polymerases IV and V, which are involved in transposon repression (Table S12c–e,k–m). At 10 DAP, the respective numbers were 327 and 394; if the logFC cutoff for DE is omitted, the respective GO terms become over-represented in 10 DAP comparisons (data not shown). Finally, no more than 30 transposon-related transcripts were upregulated compared to Sprint- 2 at any of the examined time points.

Judging by these results, the maturation timing pattern in Sprint-2 was altered compared to that in Zhewan-1 and Zhongwan-6 at the corresponding time points. Though dissimilarity in the experimental design between the present study and the work by Liu et al. limits the credibility of any inferred conclusions, we propose that Sprint-2 seeds consequently undergo maturation retardation around 10 DAP and acceleration between 10 and 20 DAP.

### 3.4. Evidence for the Non-Linear Maturation Timeline in Sprint-2

**Figure 7:**
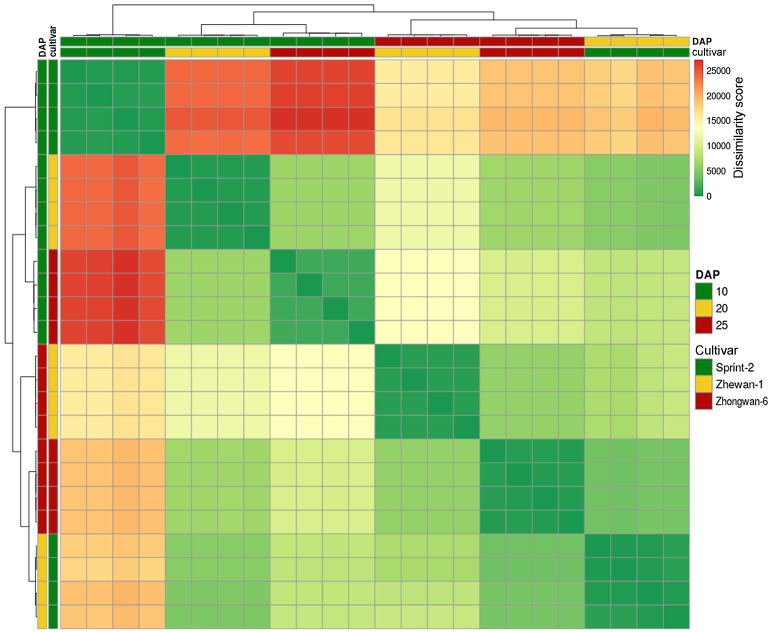
Poissonian clustering of transcriptomic samples. Cells denote transcriptomic profiles expressed for respective samples as transcript per million vectors. Color code indicates the dissimilarity measure based on the Poisson model of read distribution.

**Figure 8:**
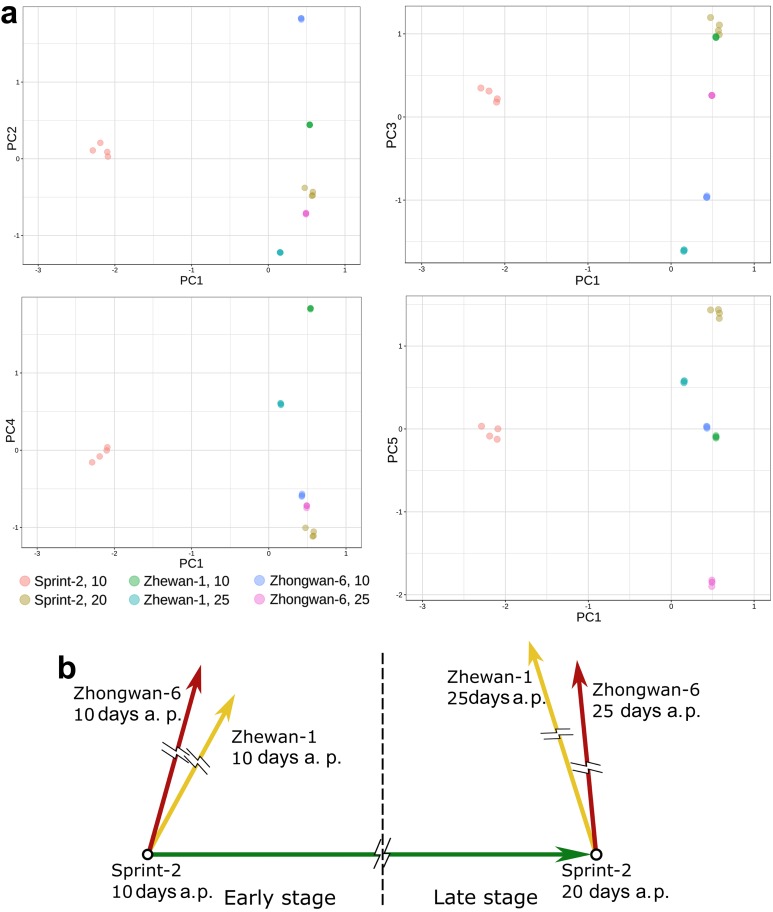
Discrepant maturation dynamics in Sprint-2 observed at a full-transcriptome scale. (**a**) Principle component analysis of three pea cultivars at two time points. Clustered values are vectors of transcripts per million values for respective samples. Primary components explain the percentage of observed variance, as follows: PC1: 60.0%; PC2: 11.9%; PC3: 8.9%; PC4: 5.2%; PC5: 4.2%. (**b**) Vectors of transcriptomic changes connecting states: from Sprint-2 10 days to Sprint-2 20 days (green vector), from Sprint-2 10 days to Zhongwan-6 10 days (red) and Zhewan-1 10 days (yellow), and from Sprint-2 20 days to Zhongwan-6 25 days (red) and Zhewan-1 25 days (yellow), are shown. Angles between vectors correspond to the arccosine of cosine similarity between vectors. The timeline of maturation is along the vector Sprint-2 10–20 days. Length of the vectors was chosen arbitrarily.

To prove the assumptions based on the DE tests, we attempted to reconstruct a uniform timeline involving all the studied samples. Poissonian clustering of expression vectors represented in transcript per million (TPM) metrics indicated an outlying position of Sprint-2 seeds at 10 DAP, while other replicates grouped more or less consistently with our expectations (Figure 7). The same idea was reproduced in PCA, in which the first primary component separated 10 DAP Sprint-2 seeds from other replicates which were in closer proximity (Figure 8a, Figure S9, Table S14). When represented in ternary form, differential expression vectors for Sprint-2 and two other pea lines exhibited angles with small negative cosine values (Figure 8b). Similar angles were formed by the Sprint-2 maturation vector and transition vectors from Sprint-2 at 20 DAP and Zhewan-1/Zhongwan-6 seeds at 25 DAP. Angles formed by the Sprint-2 maturation vector and transitions from Sprint-2 to two other cultivars at 10 DAP had positive cosines (0.48 in Zhewan-1 and 0.27 in Zhongwan-6, respectively). Taken together, these data underpin the deviant gene expression program in Sprint-2 at 10 DAP, while the onset of maturation speed-up is less obvious.

**Figure 9:**
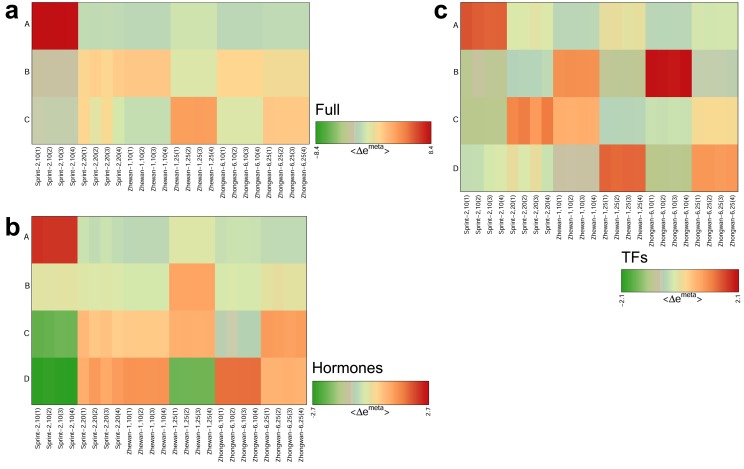
Self-organized map clustering of the seed transcriptome. Color code indicates Δe^meta^ values for each sample. (**a**) Full transcriptomic landscape. (**b**) Transcripts related to hormone metabolism and signaling. (**c**) Transcripts related to the regulation of gene expression.

Self-organizing map (SOM) clustering of the transcriptomic landscape (Figure 9a, Table S15, File S2) indicated features separating samples into several distinct groups. The group overexpression spot A separating 10 DAP Sprint-2 seeds comprised 46 transcripts enriched with terms related to peptidyl-prolyl isomerization in proteins and glycolysis. Notably, this spot included several lipid transfer proteins, abscisic acid, and cytokinin receptors. The larger spot B containing 119 transcripts separated Sprint-2 seeds at 10 DAP and Zhewan-1 seeds at 25 DAP. It contained terms related to the abscisic acid response, lipid and protein accumulation, and various stress responses. Spot C containing 70 genes was enriched with stress-related terms. These results further prove the distance between Sprint-2 seeds at 10 DAP and other time points.

### 3.5. Hallmark Features of Altered Seed Maturation at a Pathway-Scale Resolution

**Figure 10:**
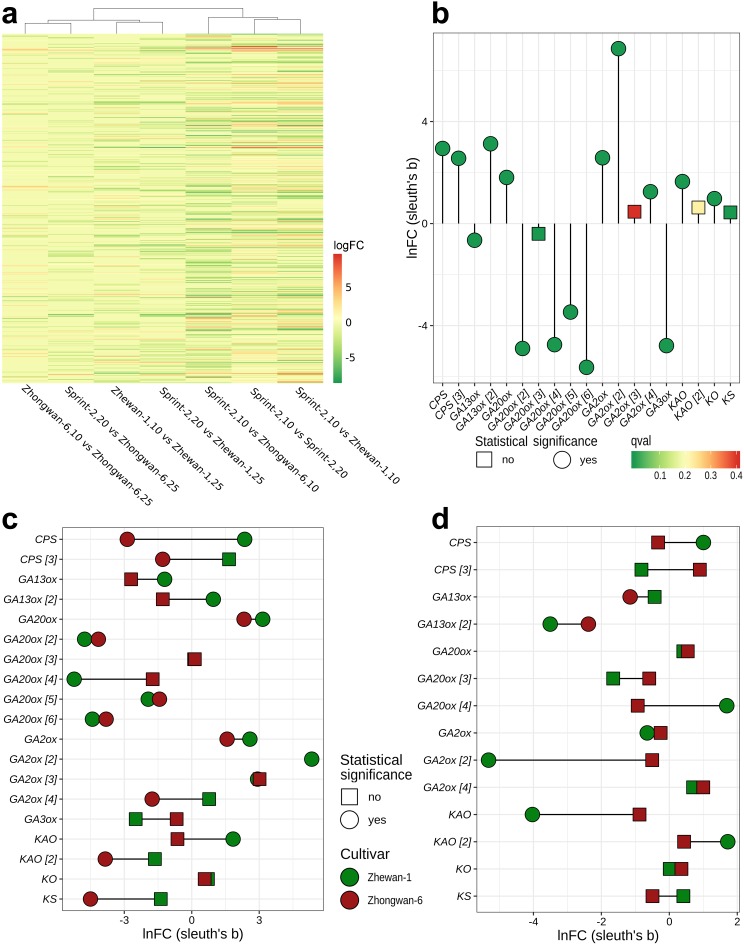
Expression dynamics of transcripts related to phytohormone biosynthesis. (**a**) Heatmap of expression changes in different comparisons. Plotted are logFC changes in the respective comparisons, where clustering was performed using Euclidean distances between samples. (**b**–**d**) Expression of transcripts related to gibberelin (GA) metabolism. Numeric axis values indicate logFC changes in respective DE tests. (**b**) Changes between 10 and 20 DAP seeds of Sprint-2. (**c**) Differential expression between Sprint-2 and Zhewan-1/Zhongwan-6 seeds at 10 DAP. (**d**) Differential expression between Sprint-2 and Zhewan-1/Zhongwan-6 mature seeds.

Assuming that the observed differences reflect altered maturation timing in Sprint-2 seeds, we next examined several transcript functional categories to find possible features of this phenomenon. We first focused our attention on the transcripts related to hormonal regulation and the regulation of transcription. The expression patterns of hormone-related genes within Sprint-2 were similar to those emerging between Sprint-2 and Zhewan-1/Zhongwan-6 seeds, which corroborates previous observations (Figure 10a). The implementation of SOM clustering revealed four group overexpression spots (Figure 9b, File S3). Of these, spot A (File S3) enriched in Sprint-2 10 DAP seeds contained 21 transcripts, most of which are related to the synthesis and degradation of phytohormones, predominantly auxin. The abundance of auxin-related transcripts is consistent with the involvement of this phytohormone in seed early development and partially underpins the idea of retardation in Sprint-2 seeds at this point (Table S16). Spot B was enriched in 25 DAP seeds of Zhewan-1 and mostly contained transcripts involved in brassinosteroid synthesis and signal transduction and, to a lesser extent, ethylene synthesis. Spot C was depleted in 10 DAP Sprint2 and Zhongwan-6 seeds. Despite being the smallest one, it harbored two essential enzymes of the gibberellin synthesis pathway, known as gibberellin 20-oxidase (GA20ox) and gibberellin 2-beta-dioxygenase (GA2ox), as well as the ABI3 transcription factor, which is one of the key regulators of seed germination. Finally, spot D with its 10 transcripts was depleted in 25 DAP seeds of Zhewan-1 and 10 DAP seeds of Sprint-2 and mostly contained stress hormone synthesis and signaling factors, including three ABA response factors.

Since the ABA/GA ratio is one of the main controlling mechanisms of seed maturation, we traced the expression of key enzymes involved in their biosynthesis. In Sprint-2, most of the enzymes specific for ABA biosynthesis were downregulated at 20 DAP, while those shared with other isoprenoid synthesis pathways were either upregulated or not differentially regulated (Figure S10a). Eight homologs of CYP707A2, which is a primary ABA catabolism agent during late seed development, were expressed in an unorchestrated way, with one transcript being downregulated, five being upregulated, and two left unaffected at 20 DAP. Compared to Sprint-2, in Zhewan-1 and Zhongwan-6 at 10 DAP, most of the CYP707A isoforms were upregulated, while ABA2 was massively downregulated (Figure S10b); in maturing seeds, both synthesis and degradation enzymes, except for ABA2 and ABA4, were downregulated compared to 20 DAP Sprint-2 seeds (Figure S10c). Gibberellin metabolism showed a more lucid mode of regulation: in Sprint-2, most of the synthesis enzymes except for GA13ox were repressed, while transcripts encoding gibberellin-inactivating enzyme GA20ox were upregulated (Figure 10b). Similar differences were observed between 10 DAP Sprint-2 seeds and those of Zhewan-1 and Zhongwan-6 seeds (Figure 9c), while in the maturing seeds, notable differences only arose in Zhewan-1 (Figure 10d). The expression dynamics of some of these transcripts, such as ABA2 from group spot A, corroborate the ideas inferred from SOM clustering.

**Figure 11:**
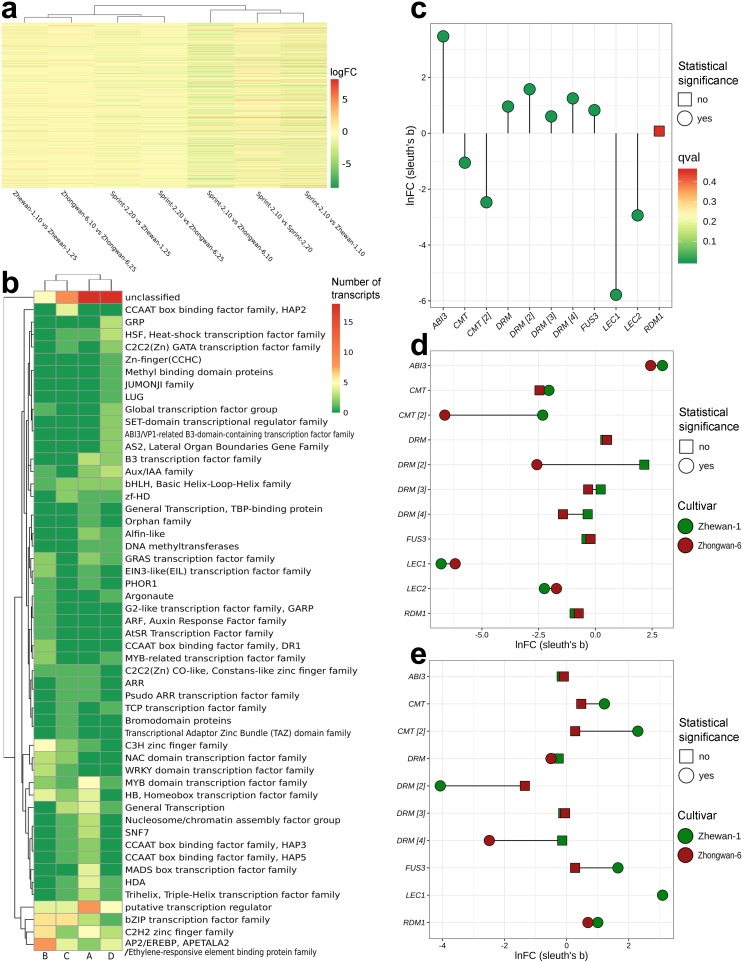
Expression dynamics of transcripts encoding transcription regulators. (**a**) Heatmap of expression changes in different comparisons. Plotted are logFC changes in the respective comparisons, where clustering was performed using Euclidean distances between samples. (**b**) Heatmap of MapMan terms related to transcription regulation affected by the differential expression. Columns denote SOM group overexpression spots A–D. Plotted are the numbers of transcripts annotated to the respective category. (**c**–**e**) Expression of transcripts encoding AFL factors and DNA methylases. Numeric axis values indicate logFC changes in respective DE tests. (**c**) Changes between 10 and 20 DAP seeds of Sprint-2. (**d**) Differential expression between Sprint-2 and Zhewan-1/Zhongwan-6 seeds at 10 DAP. (**e**) Differential expression between Sprint-2 and Zhewan-1/Zhongwan-6 mature seeds.

Euclidean clustering of transcriptional changes for transcription regulators was consistent with the previously observed patterns (Figure 11a). SOM clustering revealed four group overexpression spots in this set (File S4, Table S17). Based on the MapMan annotation, these clusters mostly comprised putative transcription factors, while most of the more definite terms were represented by single or a few transcripts (Figure 11b), with the only exception of spot B, in which AP2/EREBP was the most abundant category. This spot contained 63 transcripts enriched in Zhewan-1 and Zhongwan-6 seeds and mostly involved in stress responses and carbohydrate metabolism, according to GO annotation (Table S17). The other spot separating Zhewan-1 and Zhongwan-6 from Sprint-2 was spot D, which was enriched in 25 DAP seeds. Spot A denotes transcripts exclusively enriched in Sprint-2 10 DAP seeds and involved in mRNA binding and processing, while spot C unexpectedly aggregates mature seeds of Sprint-2 and Zhongwan-6 and 10 DAP seeds of Zhewan-1 with transcripts mostly showing the over-representation of terms relating to seed maturation and ABA signaling.

Transcription factors from the AFL group (*ABI3*, *FUS3*, and *LEC2*), together with chromatin remodeler LEC2, serve as key regulators during seed maturation. Their expression profiles across the experiment are shown in Figure 11. In Sprint-2, the expression of ABI3 and FUS3 increased during the transition from 10 to 20 DAP, while LEC1 and LEC2 became downregulated (Figure 11c). A similar pattern occurred in physiologically more mature 10 DAP seeds of Zhewan-1 and Zhongwan-6 compared to Sprint-2, though the expression of FUS3 was not affected in both cases (Figure 11d). In the 20 to 25 DAP comparison, Zhewan-1 exhibits the upregulation of LEC2 and FUS3, while none are differentially expressed in Zhongwan-6 (Figure 11e).

Following the surmise that transposable element activity in Sprint-2 might be related to DNA methylation alleviation, we also traced the expression of genes for key enzymes of this process (Figure 11c–e). The discordant mode of regulation for *CHROMOMETHYLASE* (*CMT*) and *DOMAINS REARRANGED METHYLASE* (*DRM*), which are enzymes involved in two distinct DNA methylation strategies, indicates the succession of these two programs in Sprint-2 seed maturation. In comparison, a product of *RNA- DIRECTED DNA METHYLATION 1* (*RDM1*) is involved in both DNA methylation pathways, and its expression was not altered between 10 and 20 DAP. Compared to Zhewan-1 and Zhongwan-6 seeds at 10 DAP, *CMT* transcripts were also downregulated, while in the 20 to 25 DAP comparison of both Zhewan-1 and Zhongwan-6, these patterns were inverted (Figure 11e), which, assuming that 10 DAP Sprint-2 seeds are retarded and 20 DAP seeds undergo developmental acceleration, delineates CMT- and DRM-dependent DNA methylation pathways.

**Figure 12:**
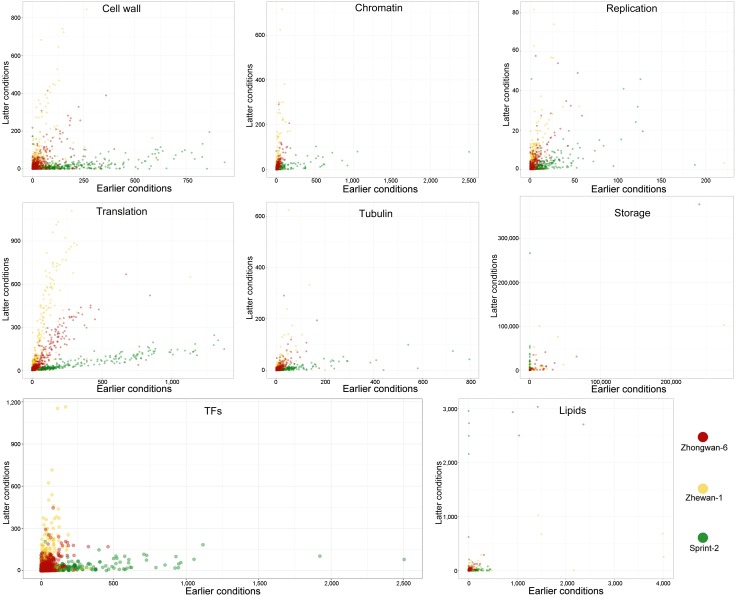
Term-wise scatterplots of early to late transcription dynamics in Sprint-2, Zhewan-1, and Zhongwan-6, where the *x*-axis denotes the mean expression at 10 DAP, and the *y*-axis denotes expression at a later (20 DAP for Sprint-2 and 25 DAP for Zhewan-1 and Zhongwan-6, respectively) stage. Transcripts belonging to the terms were selected according to the Gene Ontology annotation. For better visual interpretation, ‘Translation’ and ‘Cell wall’ scatterplots show only transcripts whose levels of expression do not exceed the indicated means; full images are presented in Figure S11.

For the rest of the process vital for seed development, transcripts attributed to respective terms were plotted with coordinates denoting the mean transcript abundance in the pre-maturation and maturation stages, respectively (Figure 12 and Figure S11). It appears that for most of the selected categories, the transcript distribution is consistently described by a simple linear regression, except for the terms related to desiccation and various stresses (data not shown). Most of the terms are close to the abscissa in Sprint-2, while in the other two lines, transcripts are scattered along the ordinate axis or medium line. The only notable exception here is the dynamics of storage protein gene expression, which seems to be absent in Sprint-2 at 10 DAP but is highly accelerated further on. For each category, outliers estimated by Cook’s distance metrics have been obtained and treated independently (Table S13). While some of these transcripts display apparent GO misannotations, others, especially those supported by particular DE analyses, represent putative cultivar key features.

Transcripts related to protein biosynthesis form nearly ideal linear dependencies in early to late coordinates (Figure 12 and Figure S11). In Zhewan-1 and Zhongwan-6, few outliers were associated with the 10 DAP axis and are represented by large ribosomal subunit proteins and SUI1-like translation initiation factors (Table S18). Replication-related terms, for instance, were sparsely dispersed because of the presumably misannotated transcripts involved in other information processes (Figure 12). Nonetheless, this reflects both active DNA replication involved in cell division and subsequent downregulation at 20 DAP, when DNA levels in the seed remain constant. An indirect corroboration for this comes from the meristem-related scatter plot (Figure S11), which shows a clear decline of the respective term in 20 DAP in Sprint-2, but not in the other two cultivars. Among the spurious outliers found in Sprint-2, a certain point lying nearly on the abscissa stands for the PCNA1 protein involved in DNA replication, which further promotes the idea of the abrupt cessation of replication at 20 DAP. For chromatin-related terms (Figure 12), several histone 1 isoforms fall out of the linear dependency, while three transcripts, encoding an HMG1 remodeling factor and H4 histones, respectively, show an especially notable dedication to the 10 DAP stage in Sprint-2.

In contrast to that, plots related to the reserve deposition show less linear dynamics (Figure 12). The most dramatic changes are observed for storage proteins, which are scattered with extreme sparsity in Zhewan-1 and Zhongwan-6, but strictly reside on the ordinate axis in Sprint-2. These data are concordant with differential expression tests, which indicated that while in Sprint-2, storage protein transcripts were absent at 10 DAP, they were massively upregulated later at 20 DAP, and in Zhewan-1 and Zhongwan-6, respective transcripts emerged at 10 DAP and were not upregulated upon the transition to 25 DAP. Triacylglycerid (TAG) synthesis exhibited an expression pattern seemingly concordant with that observed in information process transcripts (Figure 12), except for four outliers per cultivar standing for lipid droplet oleosins (Figure S11). It appears that fatty acid and TAG synthesis takes place prior to the actual forming of lipid droplets and that, in Sprint-2, lipid deposition takes places much later than in Zhewan-1 and Zhongwan-6. Finally, starch synthesis took place in Sprint-2 earlier than in Zhewan-1 and Zhongwan-6, which is consistent with the differential expression tests (Figure S11). At 20–25 DAP, most of the nine starch synthase transcripts were downregulated compared to the two cultivars, which was further indicated by the lower values of the ordinate (Figure S11f). A prominent feature observed here is the anomalous activity of alpha amylase (Figure 3) in Sprint-2 found exclusively in the earlier stage and absent at 20 DAP.

Two other features that we have previously suggested to be indicators of seed maturation are tubulin cytoskeleton plasticity and cell wall biogenesis (Figure 12). Both these categories formed nearly linear dependencies with the sustainable pattern of point distribution, but in the cell wall-related terms, storage protein transcripts were the outliers. While tubulin-associated terms were distributed consistently with the GO over-representation tests performed previously, those related to the cell wall supposedly have different dynamics, as they are upregulated in both 10 DAP (e.g., retarded) and 20 DAP (e.g., accelerated) Sprint-2 seeds. We suggest that two different transcript and/or term groups comprise this category, of which the one promoted in the 20 DAP seeds is obscured by the immense upregulation observed at 10 DAP when embryo morphogenesis and growth take place.

### 3.6. Single Nucleotide Polymorphism Sites Show a Non-Random Pattern of Distribution between Transcript Functional Groups

**Figure 13:**
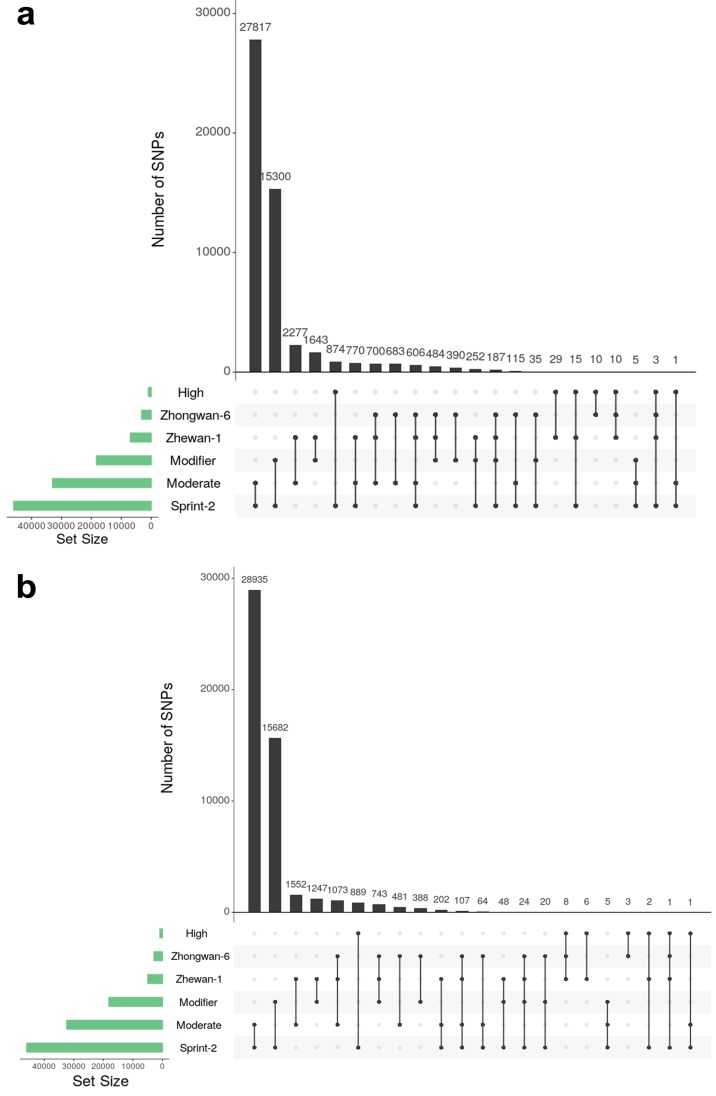
Representation of common and unique variants in three pea cultivars. Vertical bars indicate the numbers of variants falling in the respective intersection. Horizontal bars indicate absolute numbers of variants in the stated cultivar or with the stated effect. ‘High’, ‘Moderate’, and ‘Modifier’ categories refer to the respective variant effects according to snpEff2 notation. Vertical lines link cultivars and variant effects denoting respective variant intersections. (**a**) Intersection of heterozygous sites found in Sprint-2 with homozygous variants from Zhewan-1 and Zhongwan-6. (**b**) Intersection of heterozygous variants from all three studied cultivars.

A putative reason for the observed differences could lie in point mutations affecting key regulatory proteins. To test this hypothesis, we performed variant calling of Sprint-2, Zhewan-1, and Zhongwan-6 reads against a supertranscript set derived from Sprint-2 transcriptome assembly (Figure 13, Table 2). We found that Sprint-2 bore nearly 45,000 non-samesense polymorphic sites in the heterozygous state, with the mean value of 1.78 variants per transcript. Most of them were exclusive for this line and caused missense mutations (‘Moderate’ effect; see Figure 13). At the same time, 1883 and 384 of these variants were found to be present in Zhewan-1 in either a heterozygous or homozygous state, respectively. In Zhongwan-6, 946 heterozygous variants from Sprint-2 were found in a heterozygous state, while 216 were homozygous. Finally, 1956 homozygous and 1990 heterozygous variants were shared between Zhewan-1 and Zhongwan-6. Of these, 1824 homozygous variants and 1194 heterozygous ones were absent in Sprint-2, which indicates the higher level of similarity between Zhewan-1 and Zhongwan-6 than between either of them and Sprint-2.

A GO annotation of variant-bearing transcripts in Sprint-2 indicates that most of the missense, nonsense, and noncoding variants affect transcripts related to transposons and transposition-related chromatin events (Table S19), which is not surprising given the high transposon nested ORF expression reported in Sprint-2. Other over-represented categories include abscisic acid biosynthesis and reception, RNA polymerase II functional maintenance and RNA modification for noncoding variants, rRNA processing and histone modification for missense variants, and RNA processing and DNA repair for nonsense variants (Table S19, Figure S12).

We hypothesized that similar variants shared between Zhewan-1 and Zhongwan-6 could denote mutations involved in developmental acceleration in Sprint-2. Though both homozygous and heterozygous variants intersect significantly in these two cultivars, homozygous shared mutations show a more similar distribution of functions compared to heterozygous ones (Tables S20 and S21) and mostly refer to stress responses. Nonsense mutations show identical over-representation patterns because of both their small number and large intersection size, which includes common variants in pentatricopeptide (PPR)-containing RNA editing proteins. Homozygous missense variants in Zhewan-1 affect transcripts involved in abscisic acid biosynthesis and nucleosome positioning, which do not arise in Zhongwan-6, while cell metabolism-related terms show similarity to some degree in both missense and noncoding variants. To enhance the similarity analysis credibility, we tested common variants for GO over-representation separately (Table S22), which enabled us to track several novel common terms, including the gibberellin biosynthetic process in the missense subset and several terms related to translation in the noncoding variant subset.

Several master transcripts were independently addressed for the presence of SNP due to their likeliness to be involved in the observed differences. In the AFL group, ABI3 bore three common homozygous missenses in Zhewan-1 and Zhongwan-6 (Asp514Glu, Glu555Ala, and Pro581Gln), while in Zhewan-1, an exclusive heterozygous Ala400Gly also occurred. In FUS3, a single common substitution, Met267Leu, was also homozygous, together with a common 3′-UTR variant. Finally, no SNPs were reported in LEC1 and LEC2. We also investigated whether reported evidence for the impact of mutations in DICER-LIKE1 (DCL1) could take place in Sprint-2, since certain microRNAs repress precocious AFL expression [77,78]. In two of the six observed isoforms expressed in Sprint-2, two repeating heterozygous missenses (Leu77Ile, Tyr166Phe and Leu832Ile, Tyr921Phe, respectively) were found, while in Zhewan-1 and Zhongwan-6, only 5′-UTR was present. These data provide evidence for the genetic differences between the studied cultivars, which might also be responsible for maturation dynamics alteration.

## 4. Discussion

Observed transcriptomic changes in Sprint-2 seeds provide certain insights into proper maturation stage estimation for this cultivar. According to the timeline proposed for Zhewan-1 and Zhongwan-6 cultivars [33], 10 DAP seeds correspond to the pre-maturation stage (i.e., morphogenesis) and 20 DAP refer to the midmaturation stage. Respective transcriptomic landscapes fit into this timing and reflect changes occurring in the maturation stage, including the accumulation of storage moieties, cessation of cell division, and gradual reinforcement of desiccation tolerance [11]. Protein and RNA turnover pose more complex pathways, with intrinsically non-linear dynamics. For instance, among transcripts related to protein degradation, an unusually high ratio of terms related to proteasomal degradation to those standing for cytoplasmic proteases occurred at 20 DAP. Both protein categories have been reported to be promoted in both maturation and desiccation stages in Cruciferae [79,80] and at the desiccation onset in common bean (*Phaseolus vulgaris*) [32], though the most active turnover has been reported for early pre-maturation stages [29,32]. In contrast to apparently high rates of proteasomal activity, ribosomal biosynthesis decreased in Sprint-2, which serves as a marker of the end of maturation. This finding is consistent with the downregulation of methionine and S-adenosylmethionine synthases, which is proposed to be one of the indicative traits for translational slowdown [29]. RNA splicing, including alternative splicing events, in seeds has been studied more thoroughly in soybean [81] and arabidopsis [82], where the promotion of alternative splicing takes place in later maturation stages. An increasing production of DNA repair proteins previously reported for *P. vulgaris* [13,32] has been confirmed in Sprint-2. We found 156 genes related to DNA repair to be downregulated and 91 to be upregulated at 20 DAP. Though these transcripts fail to produce any GO over-representation, PCNA1, RPA, RUB1, CHD3, and H2AX are exclusively tied to the 10 DAP time point in Sprint-2, which is consistent with the data for *P. vulgaris*. While double-strand break-sensing proteins such as RAD50 and NAP1 homologs suggested as desiccation markers by Parreira et al. [32] were not upregulated in 20 DAP Sprint-2, we conclude that most of the trends related to DNA preservation in Sprint-2 seeds resemble those occurring at desiccation onset. Taken together, we assume that at 20 DAP, Sprint-2 seeds successfully transfer to desiccation or are at least at the end of late maturation.

The results that can be inferred from the comparison of Sprint-2 and Zhewan-1 and Zhongwan-6, representing two lines with no development acceleration reported, are limited in their credibility due to both differences in the experimental design and lack of biological repeats in the original study by Liu et al. [33]. Nonetheless, analysis of the most general trends, such as those reflected in GO terms, gives certain evidence on transcriptomic differences between these three lines. At 10 DAP, Sprint-2 lags in the synthesis of storage compounds and response to hypoxia-induced oxidative stress. At the same time, terms related to photosynthesis, cell wall, and plant histo- and morphogenesis were notably upregulated as if the seeds were residing in the pre-maturation stage. The advance from photosynthesis to storage deposition is a feature of the so-called green carbohydrate abundant seeds [11]. Together with the enhanced cell wall synthesis, this might explain why the carbohydrate metabolism was elevated at 10 DAP in comparison to Zhewan-1 and Zhongwan-6. This affects not only sugar catabolism, but unusual activity of a certain alpha amylase, which, together with elevated glycolysis and mitochondrial respiration, might promote early embryo linear growth, as has been shown in other plants [83,84]. Another vivid feature is the nearly complete lack of storage protein transcripts in Sprint-2 at 10 DAP. This gets more interesting since in both Zhewan-1 and Zhongwan-6, the expression of respective genes apparently meets the maximum values at 10 DAP. The upregulation of abscisic acid-related terms corroborates the credibility of this data, since it is known to promote the accumulation of storage proteins and other reserve deposition [85]. The residual DNA replication activity visible in Figure 12 in Zhewan-1 and Zhongwan-6 at 25 DAP may be related to endoreplication processes observed in cotyledon cells upon storage protein production [86,87]. Taken together, at 10 DAP, Sprint-2 seeds are physiologically retarded, rather than accelerated. This assumption is further corroborated by the huge (1753) number of transcripts commonly downregulated in 10 DAP Zhewan-1 and Zhongwan-6 seeds concordantly.

At 20 DAP, Sprint-2 seeds, on the other hand, show a definitive shift towards desiccation compared to 25 DAP seeds of the two other cultivars. Most of the differentially expressed genes center around catabolic processes, resource deposition, and cell desiccation. The prominent translational retardation (Figure 12) again indicates temporal proximity to desiccation. A plausible explanation for this phenomenon lies in the upregulated response to abscisic acid, which has been shown to alleviate translation globally upon the arrival of desiccation and dormancy [88,89]. A rapid accumulation of storage protein mRNA, LEA proteins (data not shown), and expression of lipid droplet structural component genes (Figure 12) seem to be the consequences of the same abscisic acid burst. A somewhat discrepant regulation of transcripts related to cell wall biogenesis and modification supports the hypothesis on two distinct populations of cell wall-related proteins in seed development, of which one is produced exclusively during embryo morphogenesis and the other one increases in abundance during storage accumulation [11]. Finally, at this point, the differential expression of transcripts related to double-strand DNA break repair between Sprint-2 and the two other pea lines occurs. This not only supports the hypothesis on the importance of DSB repair in desiccating seeds [13], but also indicates that repair mechanisms in seeds that have passed the maturation stage are more similar to those exploited in the pre-maturation stage than in maturing seeds.

The unexpected yet prominent feature distinguishing Sprint-2 from the two other pea cultivars is the upregulation of TE-nested reading frames at both 10 and 20 DAP. Despite the uncertainty brought about by the difference in the experimental designs, these results seem credible, since Zhewan-1 and Zhongwan-6 used for transcriptome sequencing had been grown in uncontrolled conditions exposed to stressful influences, while transposon activation is usually activated during stress [90,91]. Within Sprint-2, this upregulated state was further promoted upon the transition from 10 to 20 DAP. Previously, it was shown that DNA methylation in the CHH- and CHG context leads to transposable element silencing in maturing seeds of soybean [92,93]. However, the impact of TE activation following methylation loss in soybean on both gene expression and genome integrity seems to be neglectable [94], indicating that epigenetic TE silencing may serve as a failsafe mechanism against any spurious transposon-induced mutagenesis. Though transcriptome studies per se cannot give satisfying evidence for methylation patterns, in this work, we have found that CMT transcripts are downregulated during seed maturation in Sprint-2, and DRM transcripts are upregulated. Respective proteins target different loci during DNA methylation, with CMT being involved in CHG and CHH methylation in H1 and H3K9me2 regions and DRM being involved in the CHH methylation of heterochromatin-nested transposons [95]. Though the role of transposons in seed development has scarcely been dissected, it is known that, in soybean, a certain CACTA transposon affects the expression of anthocyanin genes via the insertion modulation of MYB master regulator activity [96], and deficiency in the DECREASE IN DNA METHYLATION1 (DDM1) SWI2/SNF2 chromatin remodeler associated with the methylated regions leads to the emergence of novel transposon-induced phenotypes [97]. Furthermore, the induction of TE mobility may enhance variability capacities. In plants, certain transposable elements are induced in stress conditions [98,99]. Since the abscisic acid insensitivity trait is favorable for stress tolerance, it is possible that a TE insertion could have affected one of the ABI genes crucial for seed development and further supported by the selection in Sprint-2, as was previously shown in *Arabidopsis* [100]. We thus suppose that enhanced transposon mutagenesis could have led to the emergence of the early maturing phenotype in Sprint-2 and that subsequent transposition events could be hidden by a phenocopy or negatively selected. The observed TE upheaval in Sprint-2 might be, on the other hand, a consequence of DNA hypomethylation occurring due to a lack of functional enzyme copies. For instance, the inactivation of CMT and DRM methyltransferases expressed discordantly in Sprint-2 affects a small number of genes related to the stress response and anatomical structure formation [94].

Taken together, the obtained results indicate that the dynamics of seed development in the early maturing pea cultivar Sprint-2 significantly differ from those observed in cultivars with a common growth and development rate. While 10 DAP seeds show signatures of the pre-maturation stage, such as cell division, cell wall synthesis, tubulin cytoskeleton plasticity, and effective photosynthesis, at 20 DAP, Sprint-2 seeds stand at the desiccation onset, as indicated by translation impairment, the depletion of cell catabolism, nutrient accumulation, and the acute response to hypoxia and dehydration. Certain possible mechanisms, including DNA methylation depletion, transposable element activation, the differential expression of master regulators, point mutations, and abscisic acid signaling are proposed to explain the observed phenomenon.

## Figures and Tables

**Table 1:** Assembly statistics of seed transcriptomes.

Assembly	Per Transcript Statistics			Longest Isoform Statistics			Percent of Reads Aligned
	N50	Median Contig Length	Average Contig Length	N50	Median Contig Length	Average Contig Length	
**Initial**	1970	781	1199.79	1677	461	901.07	98.83
**Final**	2034	1415	1636.26	2073	1454	1665.17	77.62

N50 statistics are given in nucleotides. Read alignment statistics are given according to the bowtie2 assay. For the full statistical report, see Table S2 and File S2.

**Table 2:** Distribution of single nucleotide polymorphisms in three pea cultivars.

Cultivar	Moderate		High		Modifier	
	Homozygous	Heterozygous	Homozygous	Heterozygous	Homozygous	Heterozygous
**Sprint-2**	NA *	29,314	NA *	893	NA *	15,799
**Zhewan-1**	2934	4353	17	57	2062	2566
**Zhongwan-6**	1725	2104	12	23	1175	1096

* Homozygous variants found in Sprint-2 have been excluded from the analysis.

## Supplementary Materials

The following are available online at https://www.mdpi.com/2073-4409/9/3/779/s1: Table S1: An overview of libraries used in the RNA-Seq assay; Table S2: Sprint-2 transcriptome assembly statistics; Table S3: Complete transcriptome assembly annotation; Table S4: Number of differentially expressed genes (DEGs) from all of the performed comparisons; Table S5: Transcripts differentially expressed upon the transition from 10 to 20 DAP in Sprint-2; Table S6: Combined transcriptome assembly annotation; Table S7: Differential expression in Sprint-2 seeds estimated by alignment to the combined transcriptome assembly; Table S8: Differential expression in Zhewan-1 and Zhongwan-6 seeds estimated by alignment to the combined transcriptome assembly; Table S9: Differential expression in 10 DAP seeds of Zhewan-1 and Zhongwan-6 pea cultivars in comparison to Sprint-2; Table S10: Transcripts concordantly downregulated in Zhewan-1 and Zhongwan-6 compared to Sprint-2 at 10 DAP; Table S11: Transcripts concordantly upregulated in Zhewan-1 and Zhongwan-6 compared to Sprint-2 at 10 DAP; Table S12: Differential expression in maturing seeds of Zhewan-1 and Zhongwan-6 pea cultivars in comparison to Sprint-2; Table S13: Transcripts concordantly up- and downregulated in Zhewan-1 and Zhongwan-6 at 25 DAP compared to Sprint-2 at 20 DAP; Table S14: Annotation of eigenvector summands for five primary components from full transcriptome PCA; Table S15: Functional annotation of transcriptomic landscape overexpression spots according to self-organizing map clustering; Table S16: Functional annotation of transcriptomic landscape overexpression spots restricted to transcripts related to hormone signaling and metabolism; Table S17: Functional annotation of transcriptomic landscape overexpression spots restricted to transcripts related to gene expression; Table S18: Outlier transcripts based on term-wise linear regression models; Table S19: Gene Ontology over-represented terms in transcripts bearing heterozygous variants in Sprint-2; Table S20: Gene Ontology over-represented terms in variant-marked transcripts in Zhewan-1; Table S21: Gene Ontology over-represented terms in variant-marked transcripts in Zhognwan-6; Table S22: Gene Ontology over-represented terms for common variants in Zhewan-1 and Zhongwan-6; Figure S1: Intralinear differential expression in Sprint-2 assessed by aligning to the combined reference; Figure S2: Intralinear differential expression n Zhewan-1 and Zhongwan-6 assessed by aligning to the combined reference; Figure S3: A full UpSet representation of the differentially expressed genes from all the performed comparisons; Figure S4: Gene Ontology over-represented terms in transcripts concordantly downregulated in Zhewan-1 and Zhongwan-6 compared to Sprint-2 at 10 DAP; Figure S5: Gene Ontology over-represented terms in transcripts concordantly downregulated in Zhewan-1 and Zhongwan-6 at 10 DAP and in Sprint-2 at 20 DAP compared to Sprint-2 at 10 DAP; Figure S6: Gene Ontology over-represented terms in transcripts concordantly upregulated in Zhewan-1 and Zhongwan-6 compared to Sprint-2 at 10 DAP; Figure S7: Gene Ontology over-represented terms in transcripts concordantly upregulated in Zhewan-1 and Zhongwan-6 at 25 DAP compared to Sprint-2 at 20 DAP; Figure S8: Gene Ontology over-represented terms in transcripts concordantly downregulated in Zhewan-1 and Zhongwan-6 at 25 DAP compared to Sprint-2 at 20 DAP; Figure S9: Principle component analysis of three pea cultivars at two time points; Figure S10: Expression of transcripts related to ABA metabolism in three cultivars at two time points; Figure S11: Term-wise scatterplots of early to late transcription dynamics in Sprint-2, Zhewan-1, and Zhongwan-6; Figure S12: Gene Ontology over-representation in transcripts marked with heterozygous variants in Sprint-2; File S1: rnaQUAST output for the Sprint-2 transcriptome assembly; File S2: oposSOM output for the full transcriptomic landscape; File S3: oposSOM output for transcripts related to hormone metabolism and signaling; File S4: oposSOM output for transcript related to gene expression regulation. All scripts used in this work are available at https://github.com/lab7arriam/Cells_2020.
