# Supplementary figures and images for: Transcriptomic Insights into Mechanisms of Early Seed Maturation in the Garden Pea (Pisum sativum L.)
Source: Cells. 2020 Mar 23;9(3):779. doi: 10.3390/cells9030779 (PMC7140803; doi:10.3390/cells9030779)

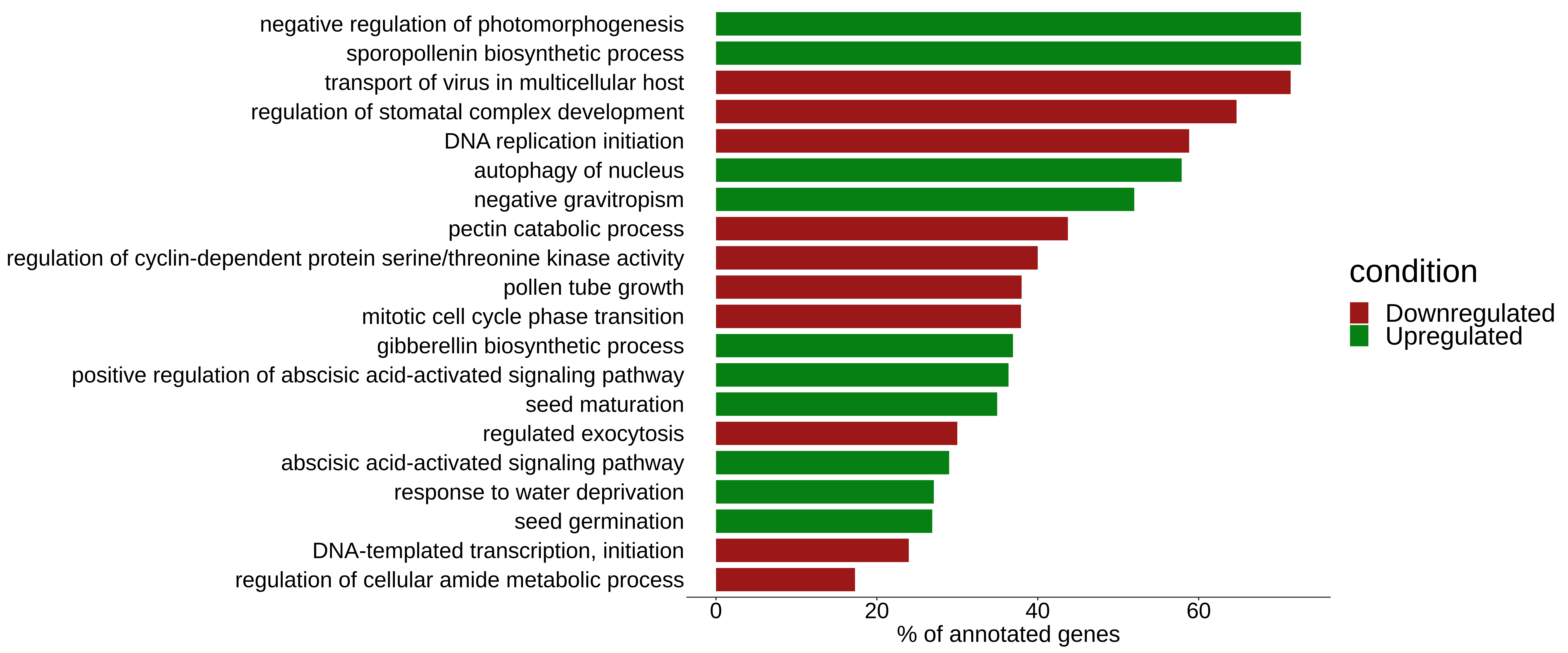

Supplement: Supplementary file 1 [file cells-09-00779-s001.zip › Supplementary materials/FigS1/BP.tiff]

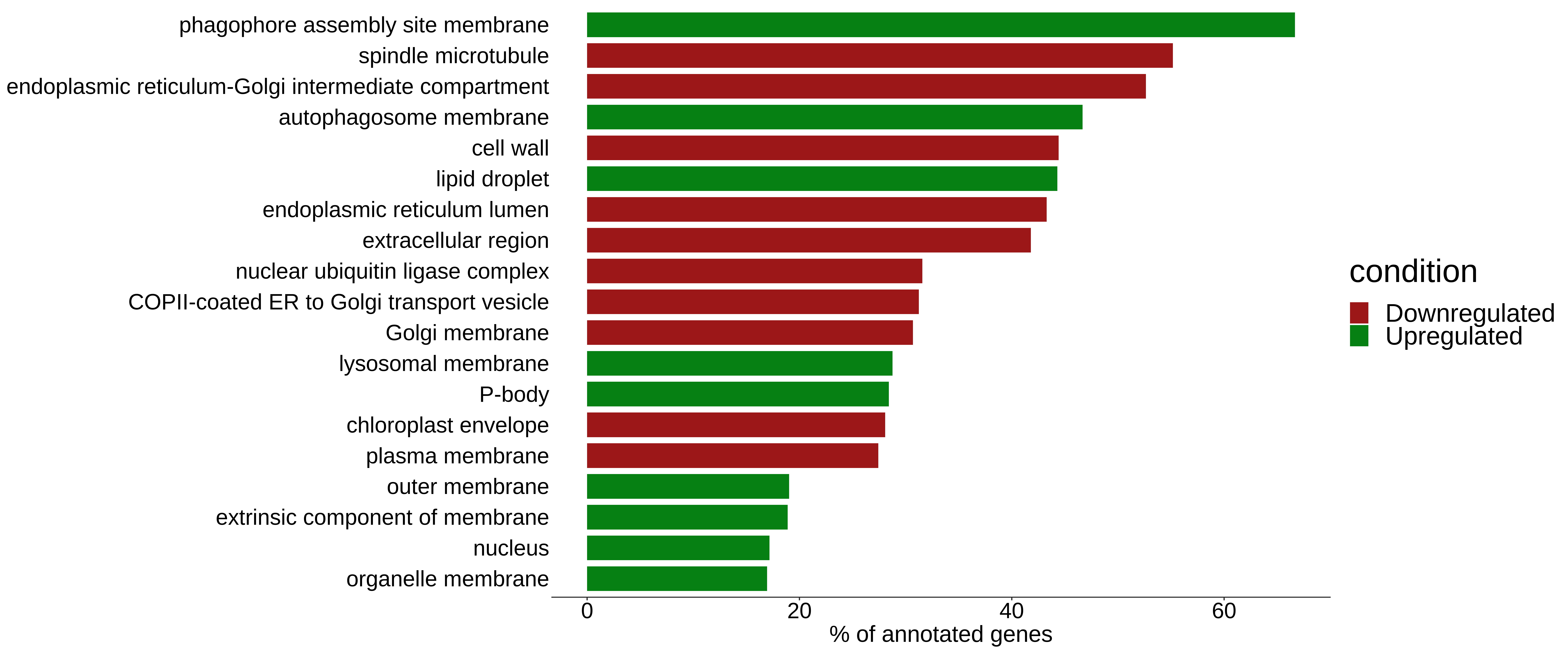

Supplement: Supplementary file 1 [file cells-09-00779-s001.zip › Supplementary materials/FigS1/CC.tiff]

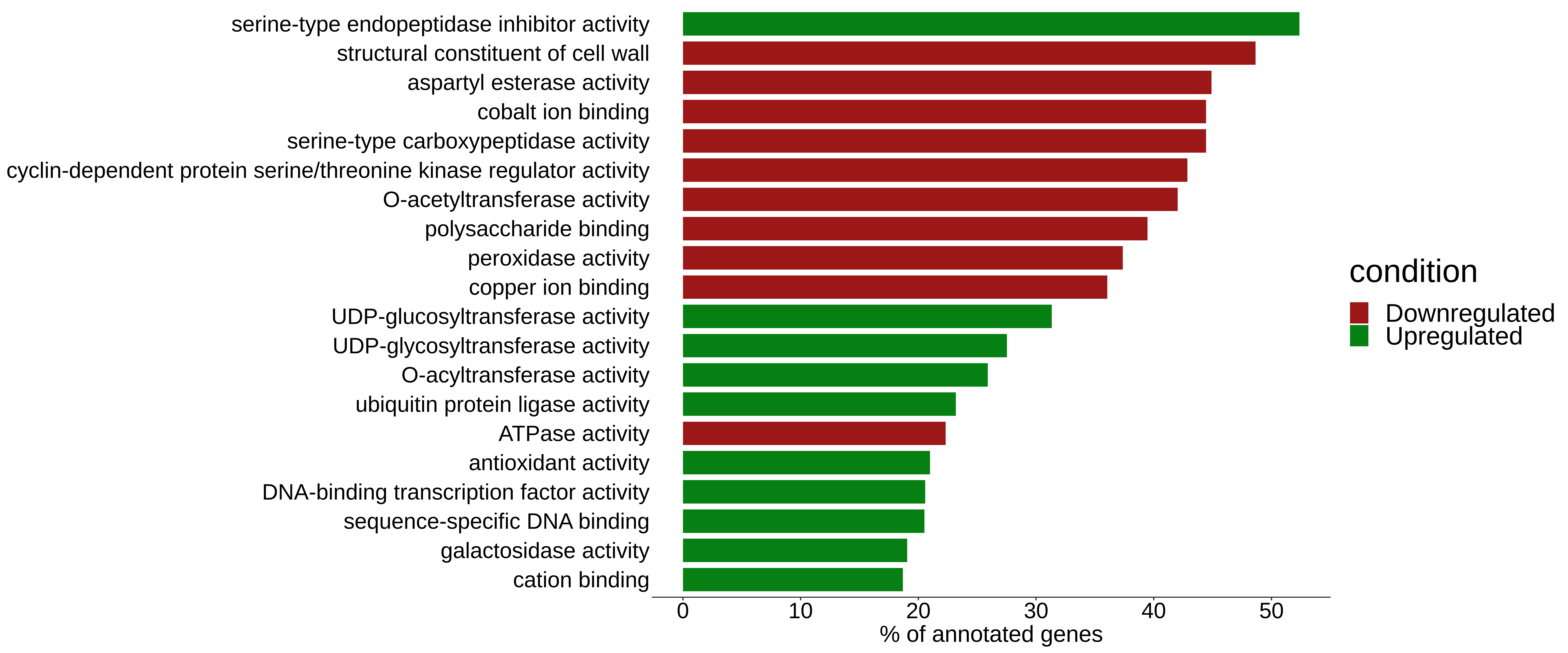

Supplement: Supplementary file 1 [file cells-09-00779-s001.zip › Supplementary materials/FigS1/MF.tiff]

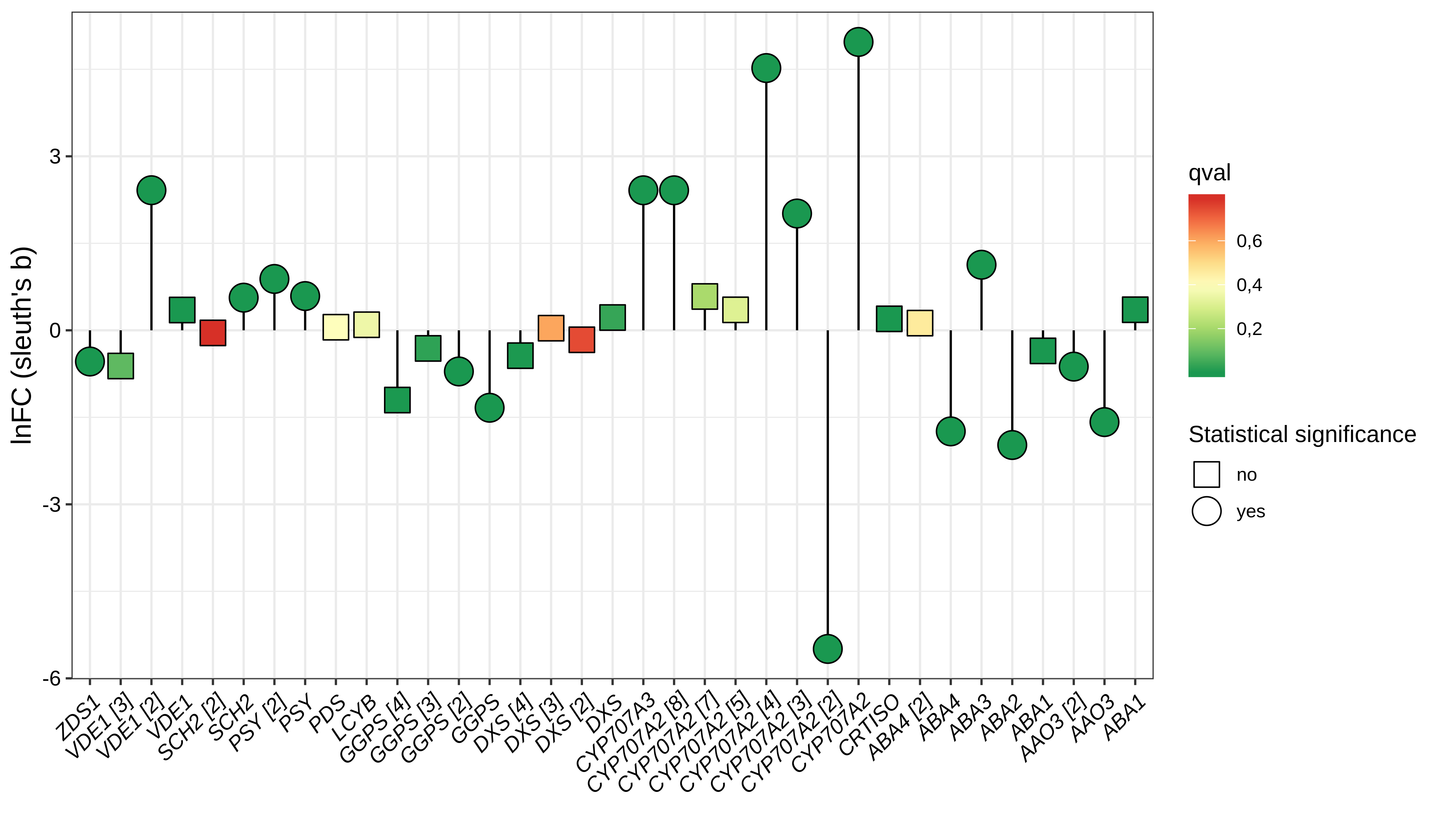

Supplement: Supplementary file 1 [file cells-09-00779-s001.zip › Supplementary materials/FigS10/A.tff]

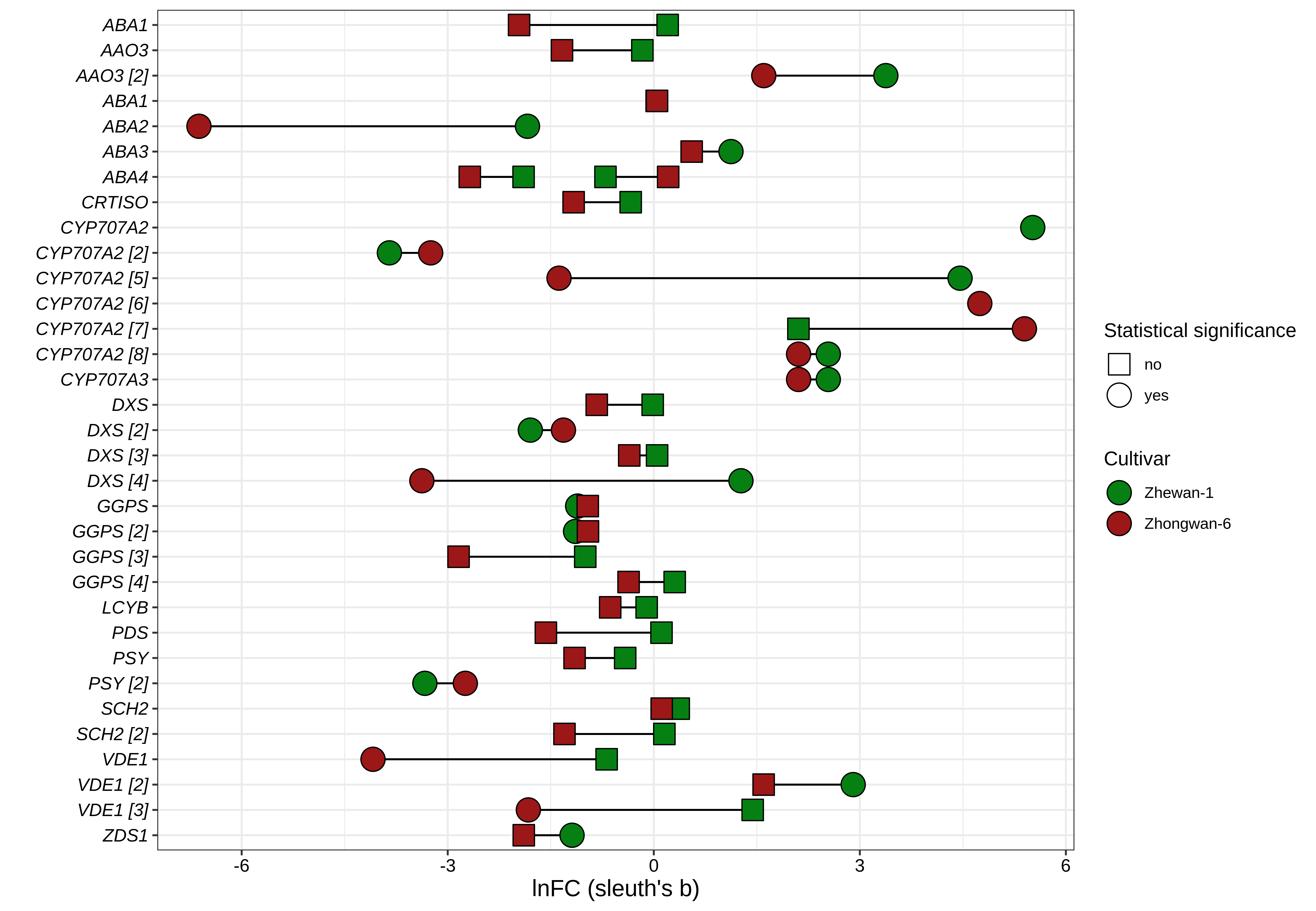

Supplement: Supplementary file 1 [file cells-09-00779-s001.zip › Supplementary materials/FigS10/B.tiff]

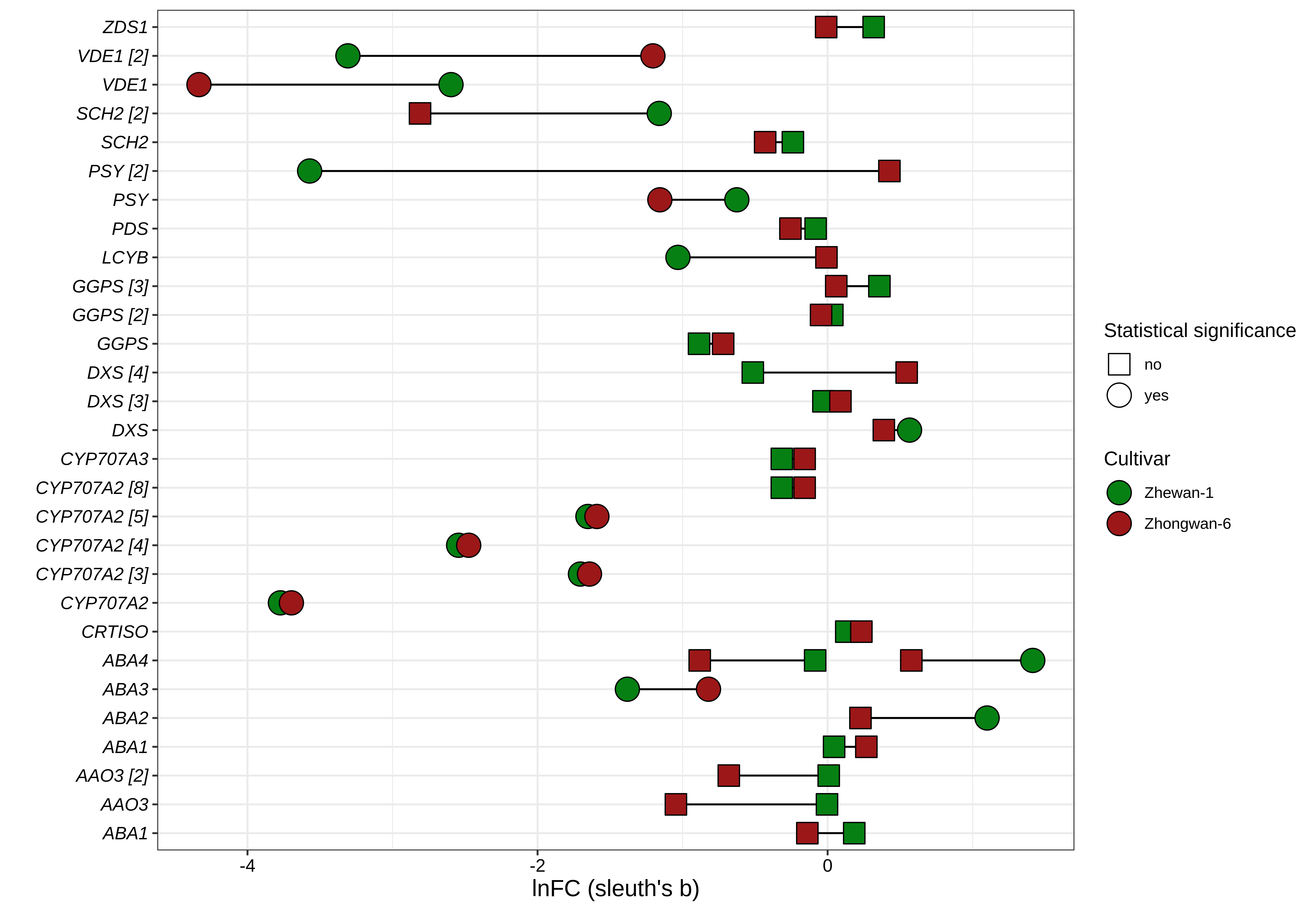

Supplement: Supplementary file 1 [file cells-09-00779-s001.zip › Supplementary materials/FigS10/C.tiff]

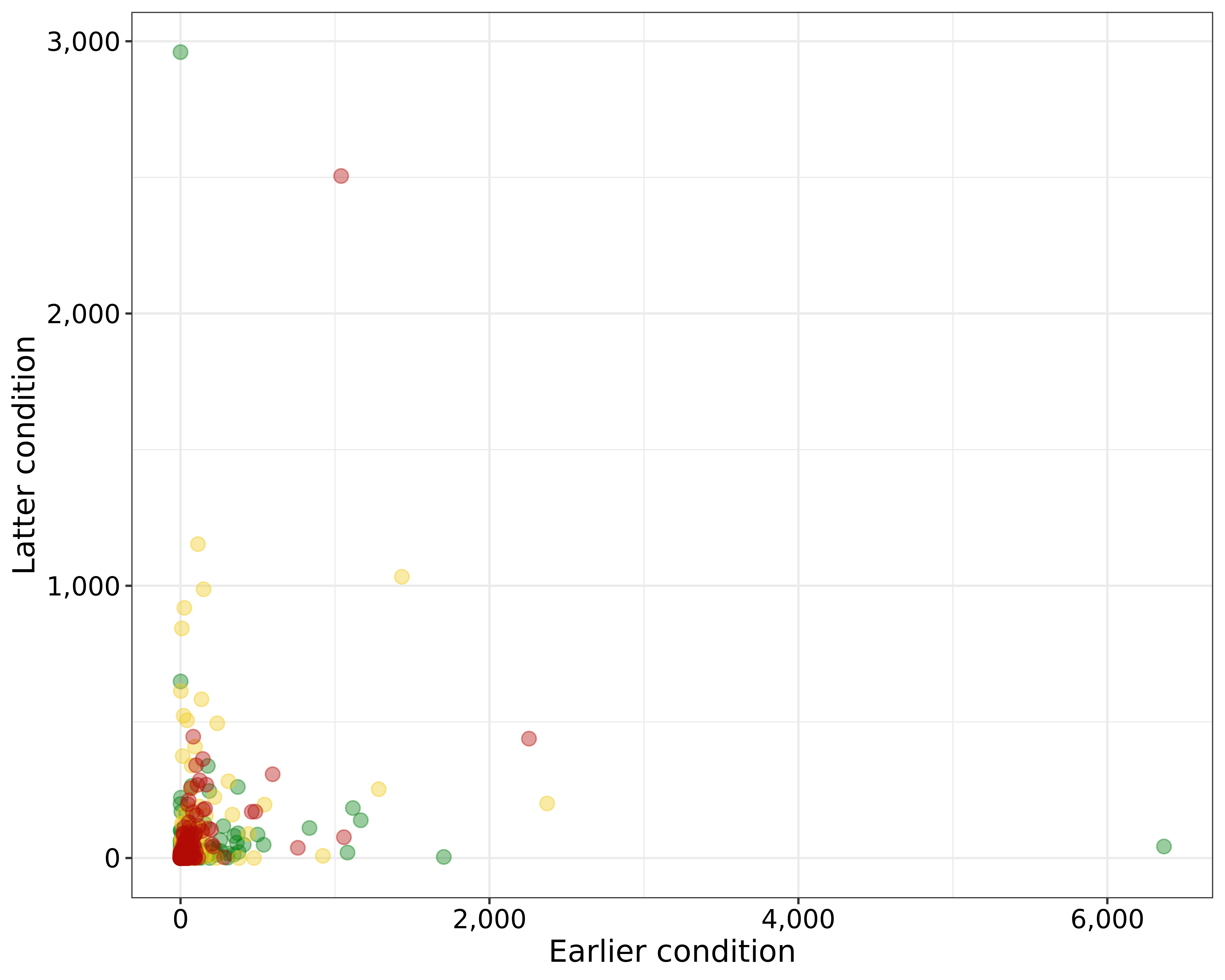

Supplement: Supplementary file 1 [file cells-09-00779-s001.zip › Supplementary materials/FigS11/1.tif]

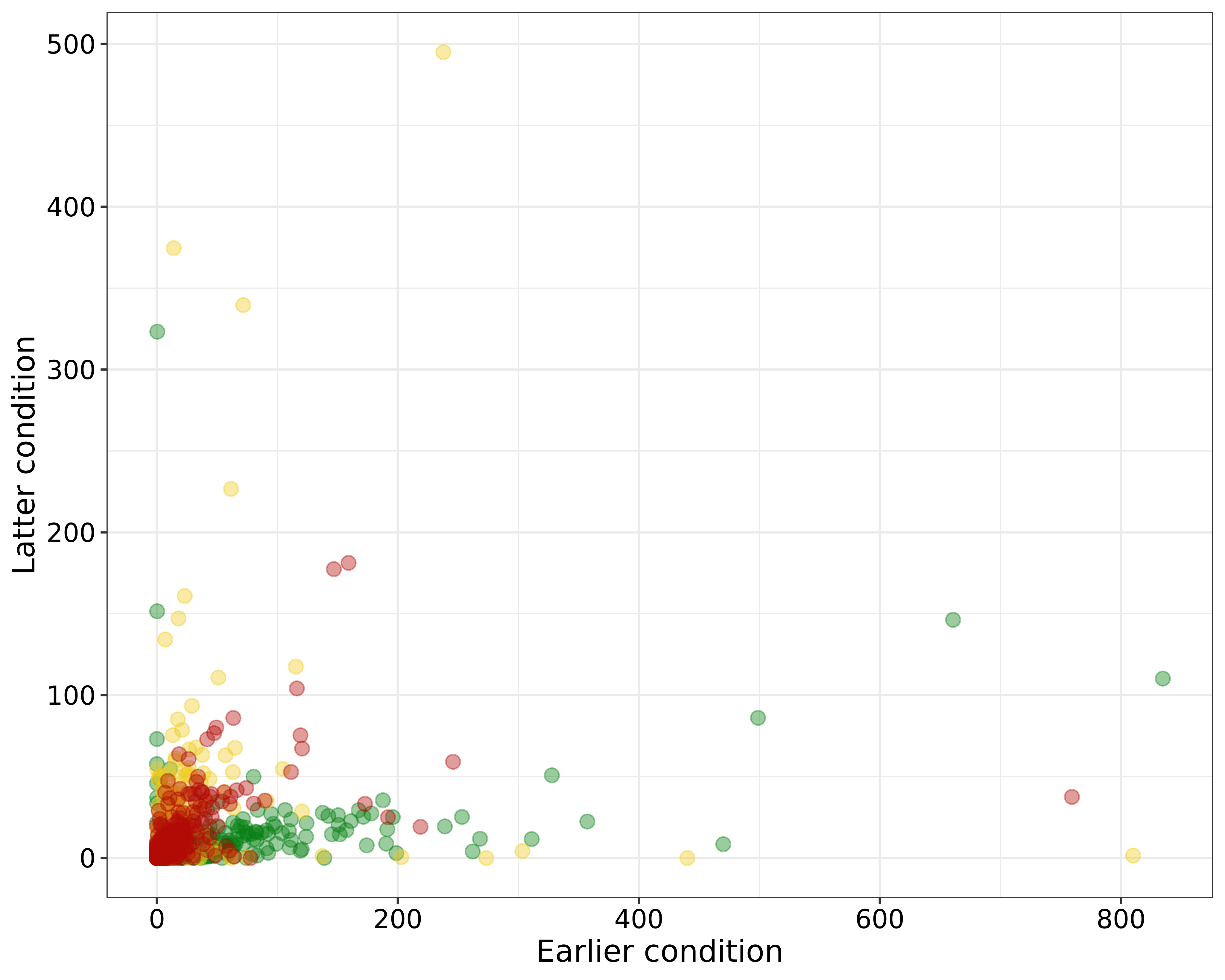

Supplement: Supplementary file 1 [file cells-09-00779-s001.zip › Supplementary materials/FigS11/10.tif]

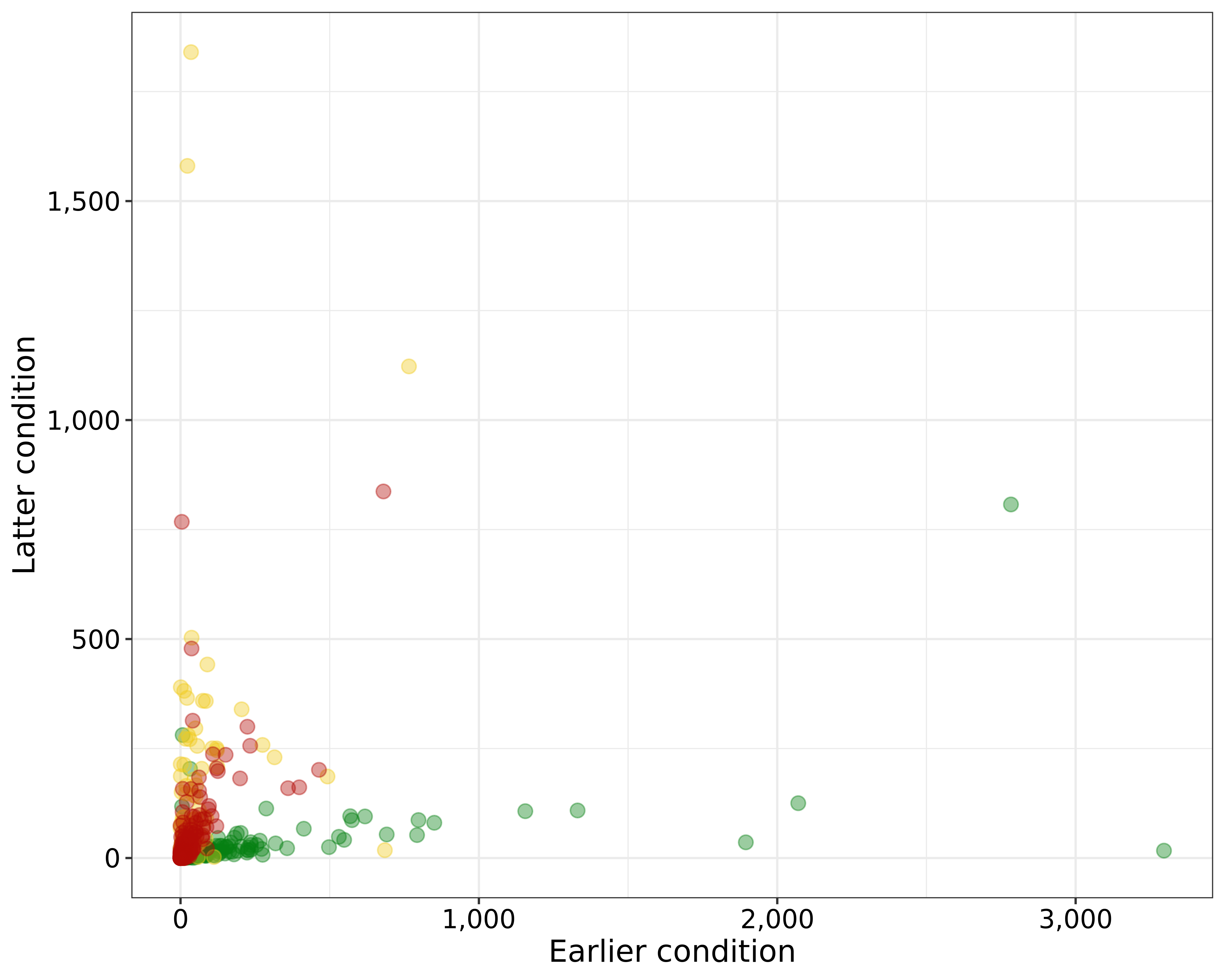

Supplement: Supplementary file 1 [file cells-09-00779-s001.zip › Supplementary materials/FigS11/11.tif]

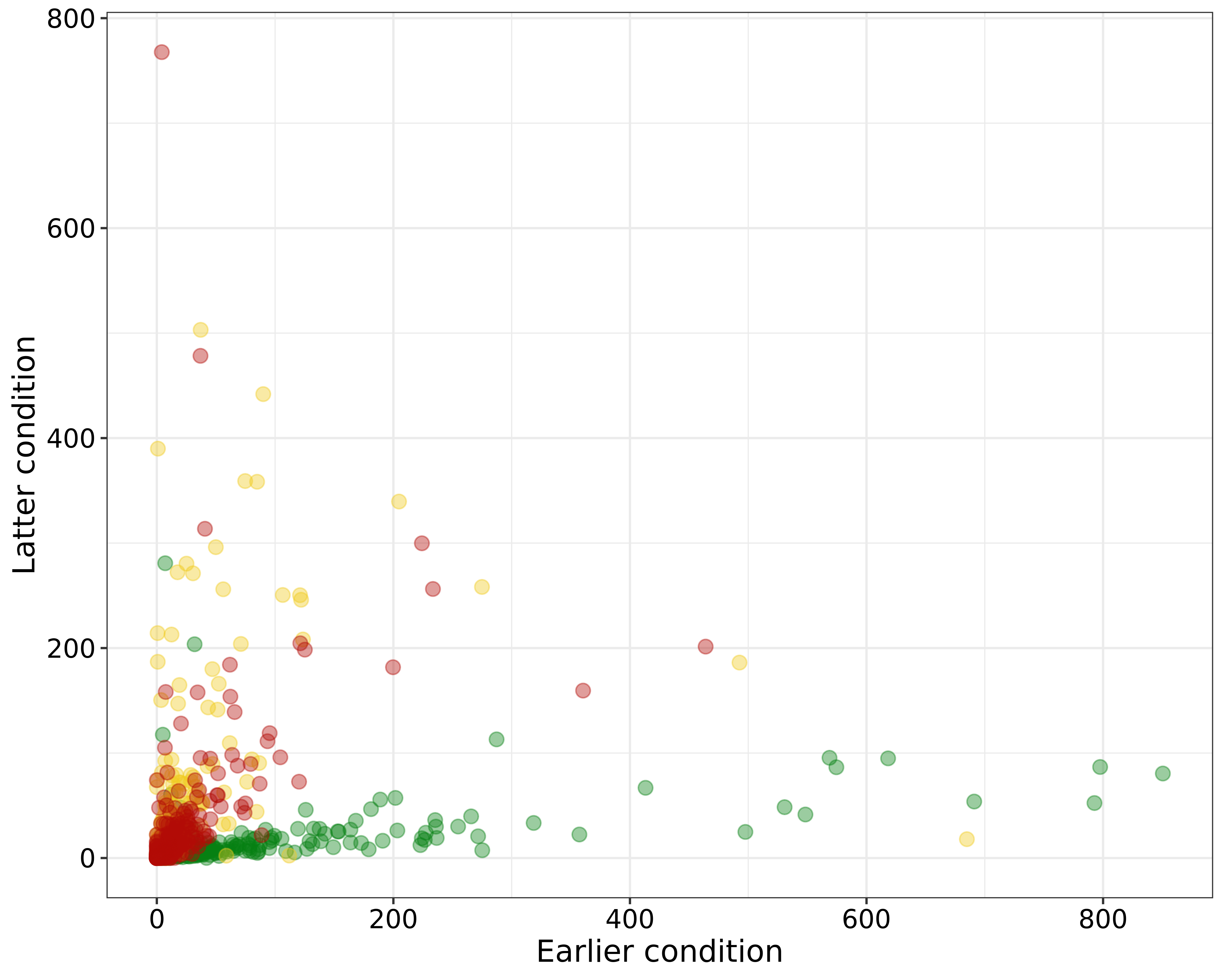

Supplement: Supplementary file 1 [file cells-09-00779-s001.zip › Supplementary materials/FigS11/12.tif]

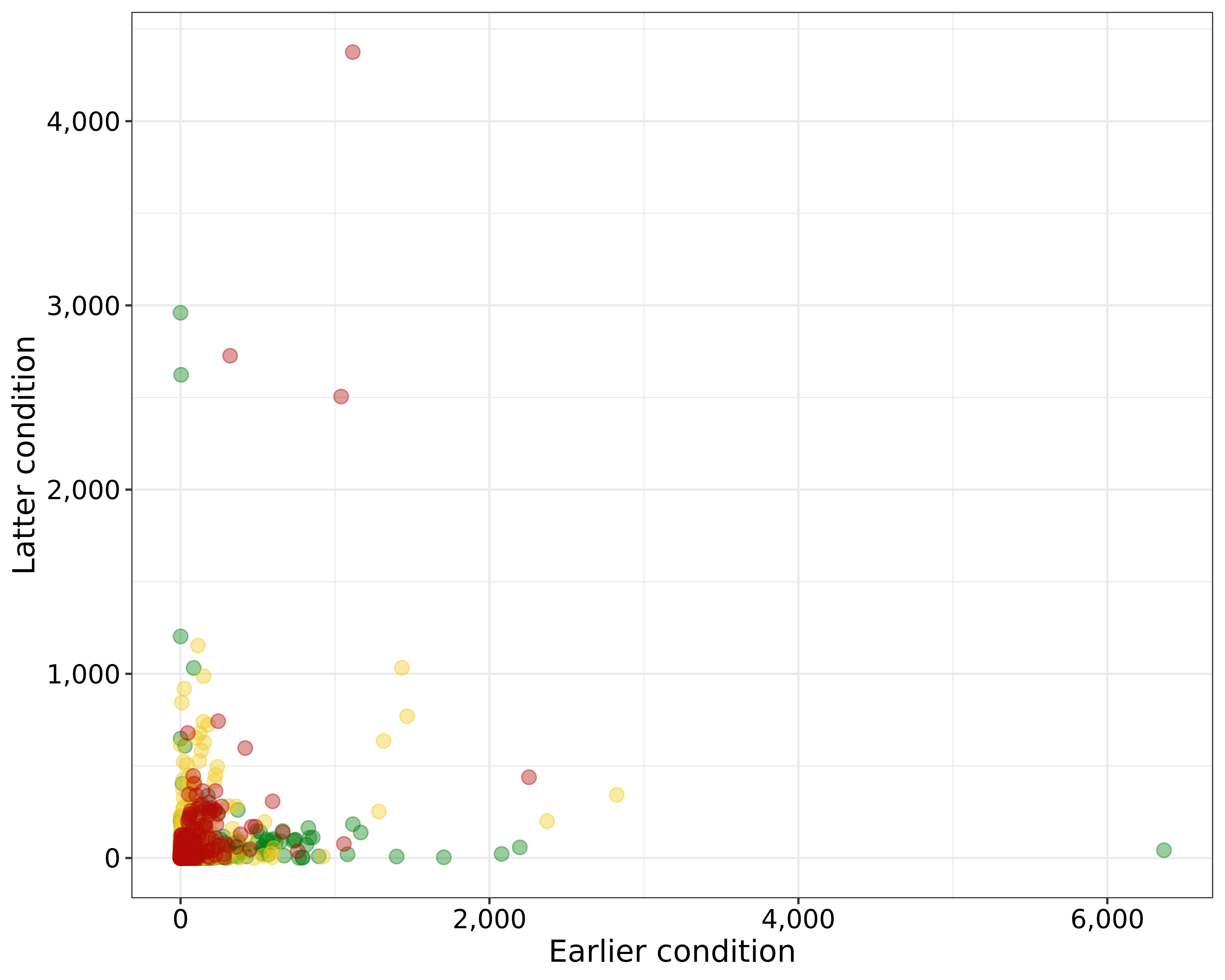

Supplement: Supplementary file 1 [file cells-09-00779-s001.zip › Supplementary materials/FigS11/13.tif]

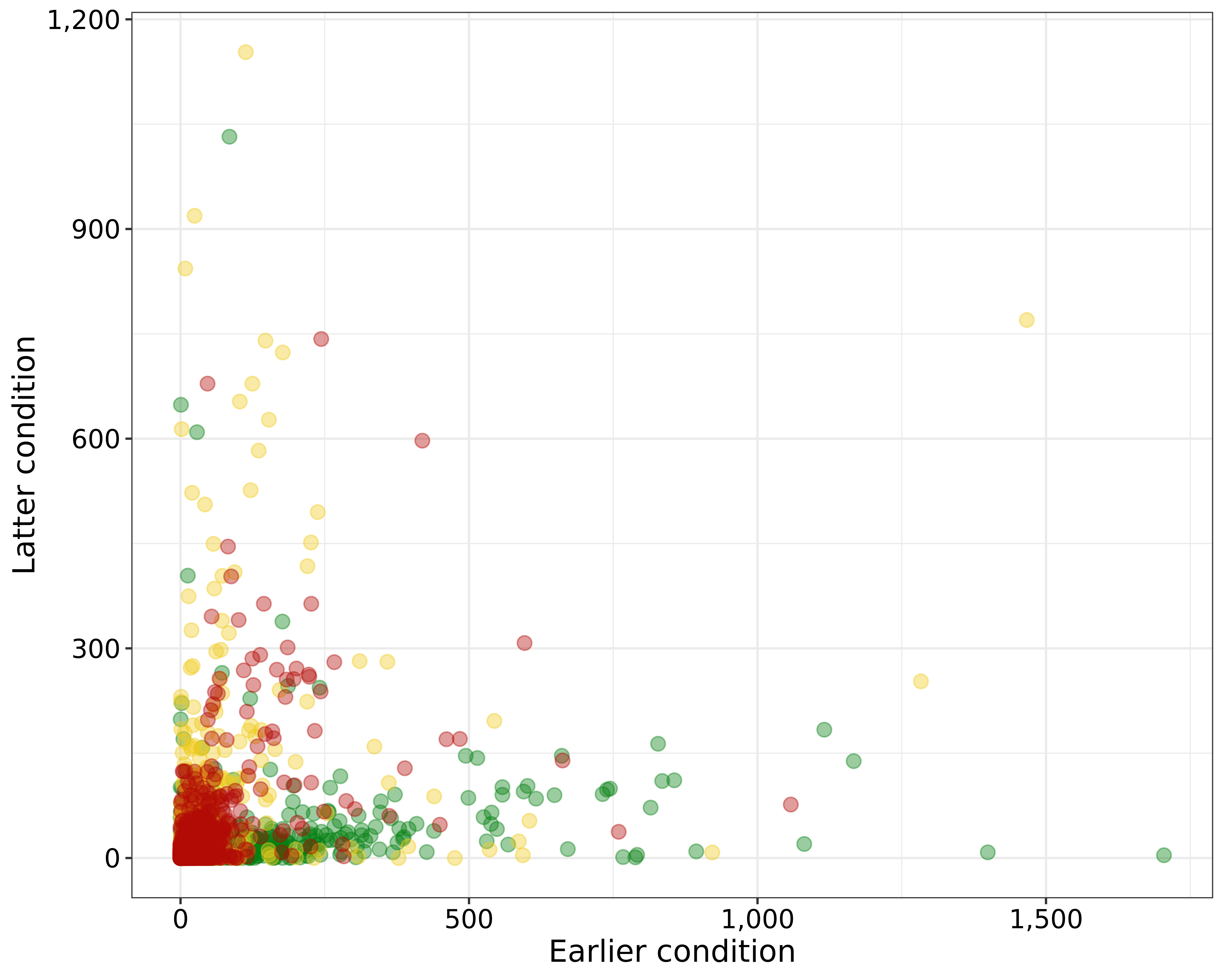

Supplement: Supplementary file 1 [file cells-09-00779-s001.zip › Supplementary materials/FigS11/14.tif]

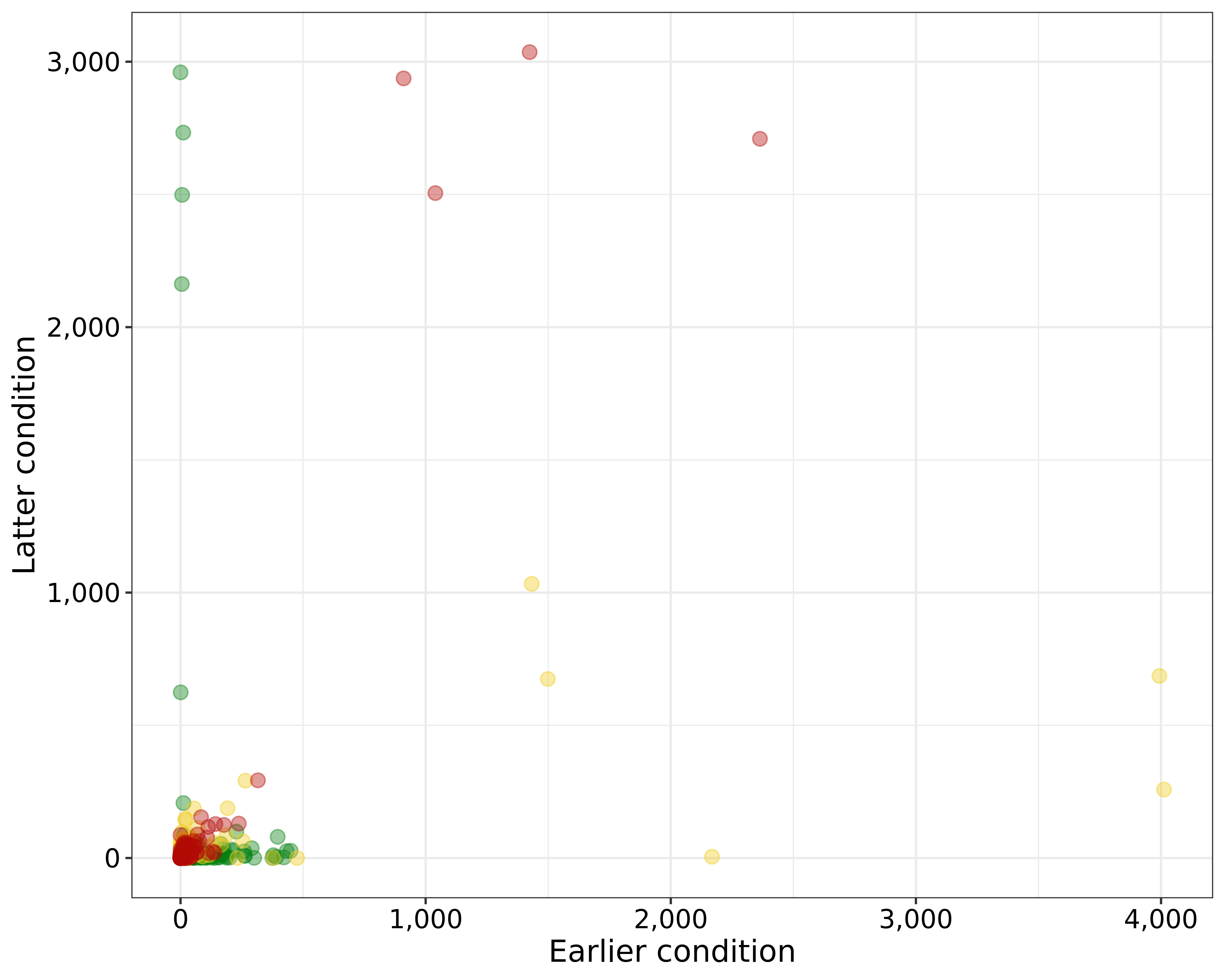

Supplement: Supplementary file 1 [file cells-09-00779-s001.zip › Supplementary materials/FigS11/15.tif]

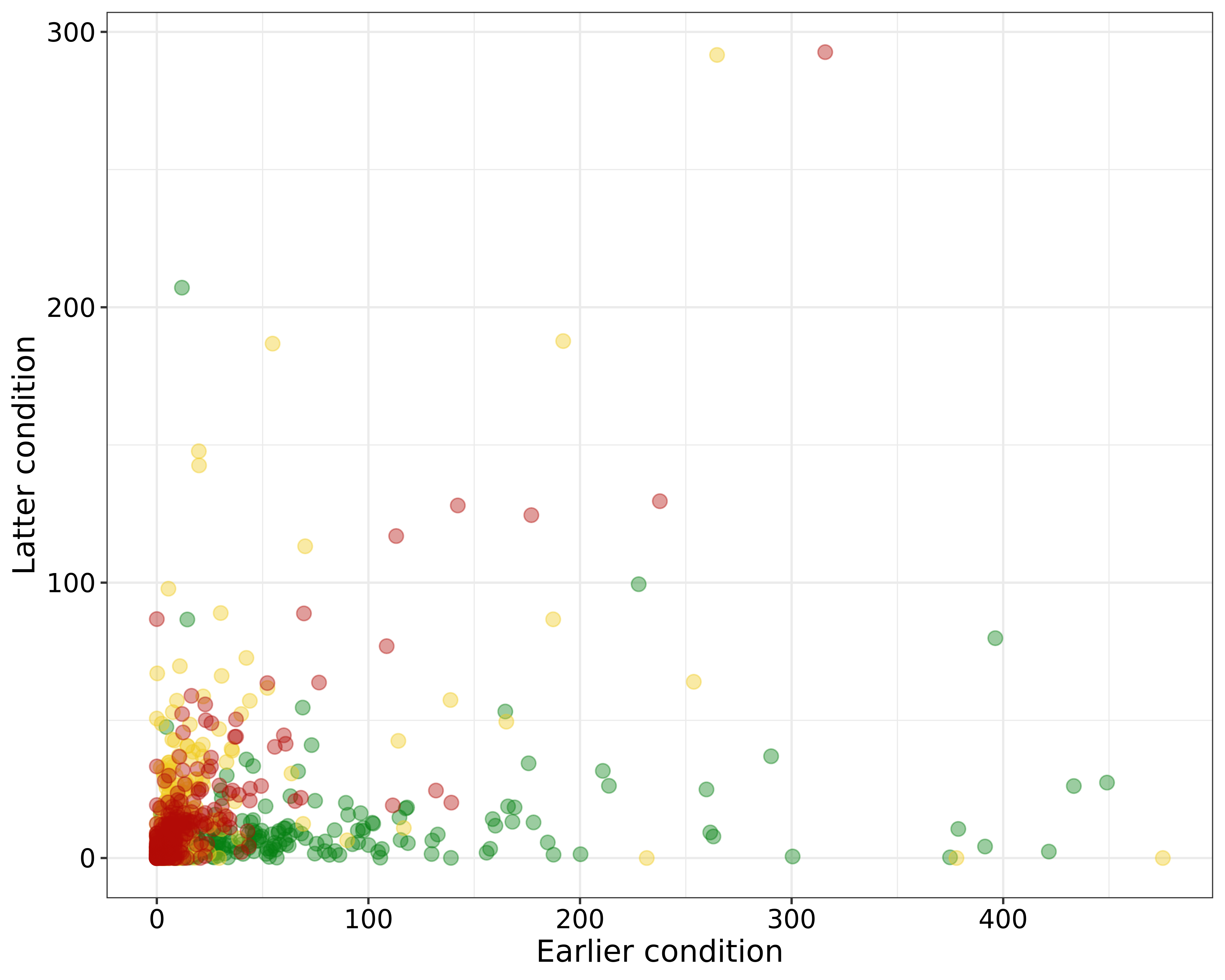

Supplement: Supplementary file 1 [file cells-09-00779-s001.zip › Supplementary materials/FigS11/16.tif]

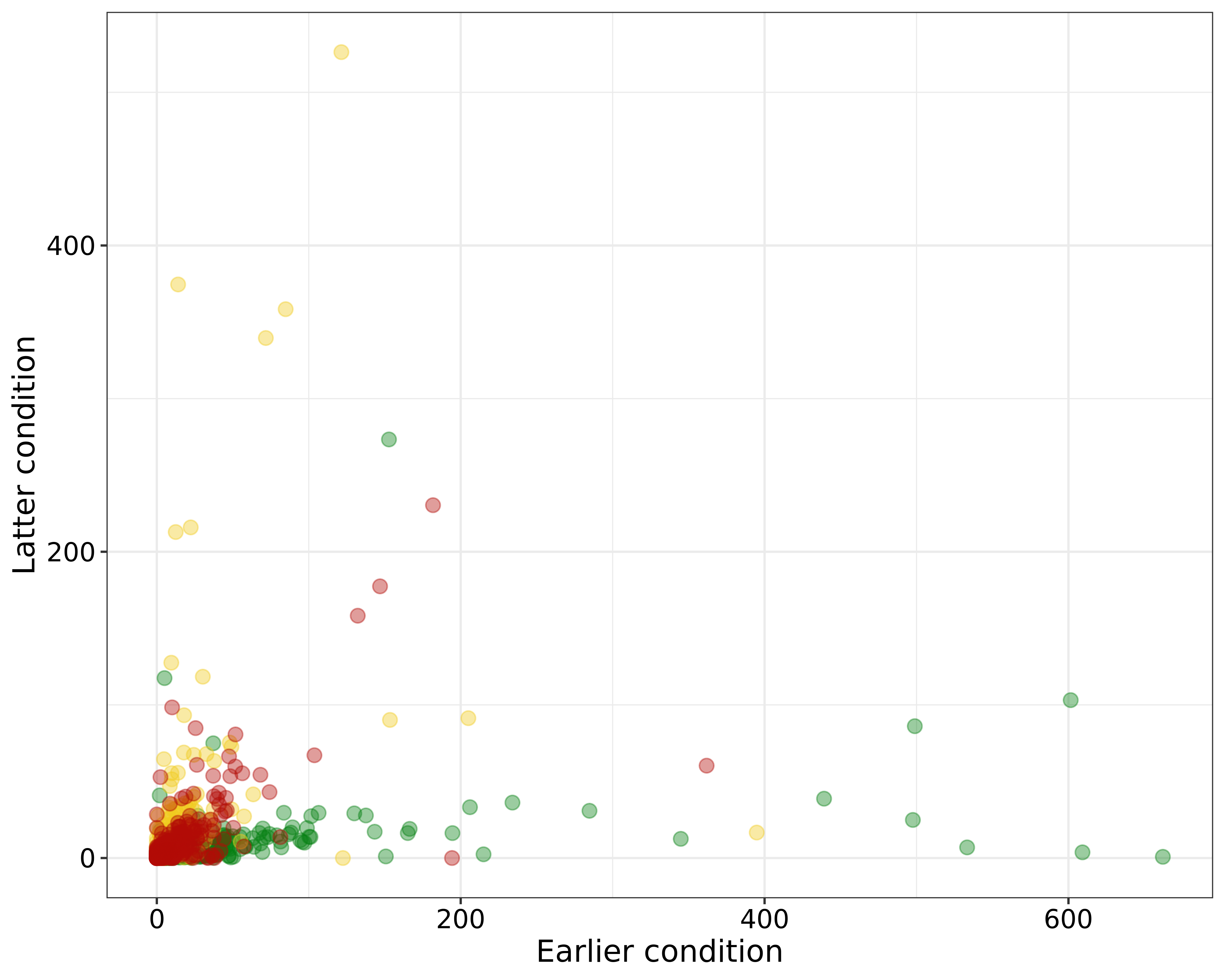

Supplement: Supplementary file 1 [file cells-09-00779-s001.zip › Supplementary materials/FigS11/17.tif]

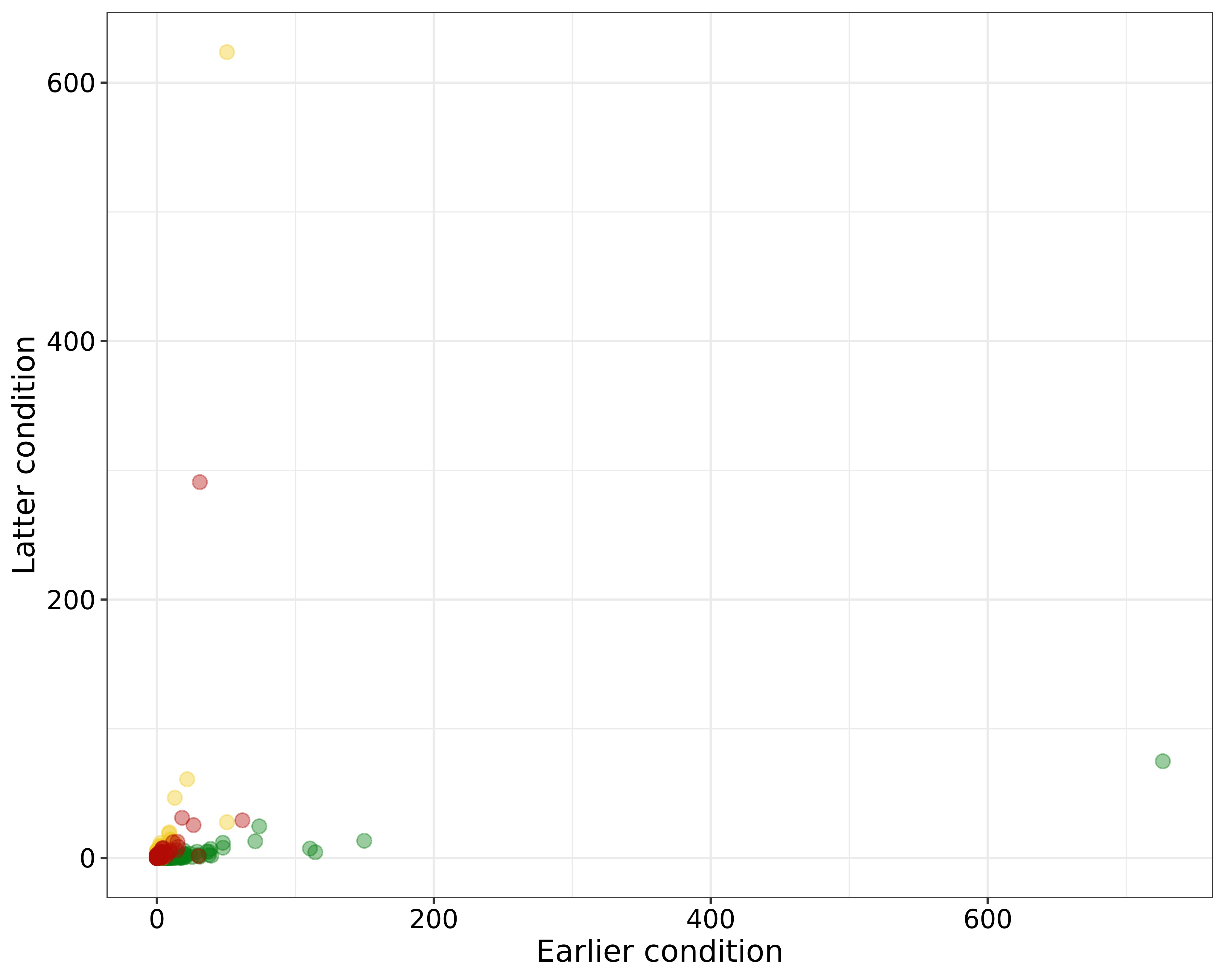

Supplement: Supplementary file 1 [file cells-09-00779-s001.zip › Supplementary materials/FigS11/18.tif]

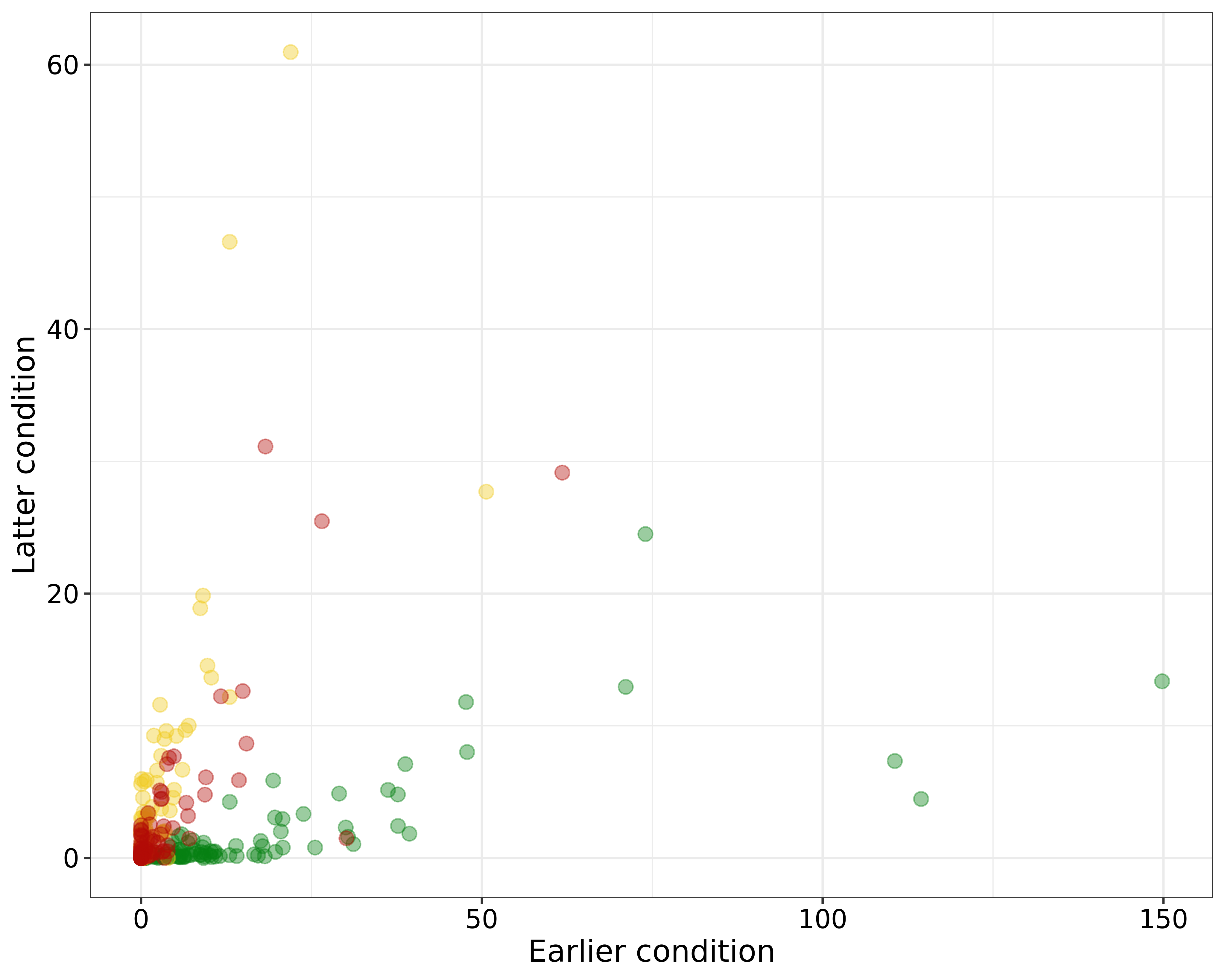

Supplement: Supplementary file 1 [file cells-09-00779-s001.zip › Supplementary materials/FigS11/19.tif]

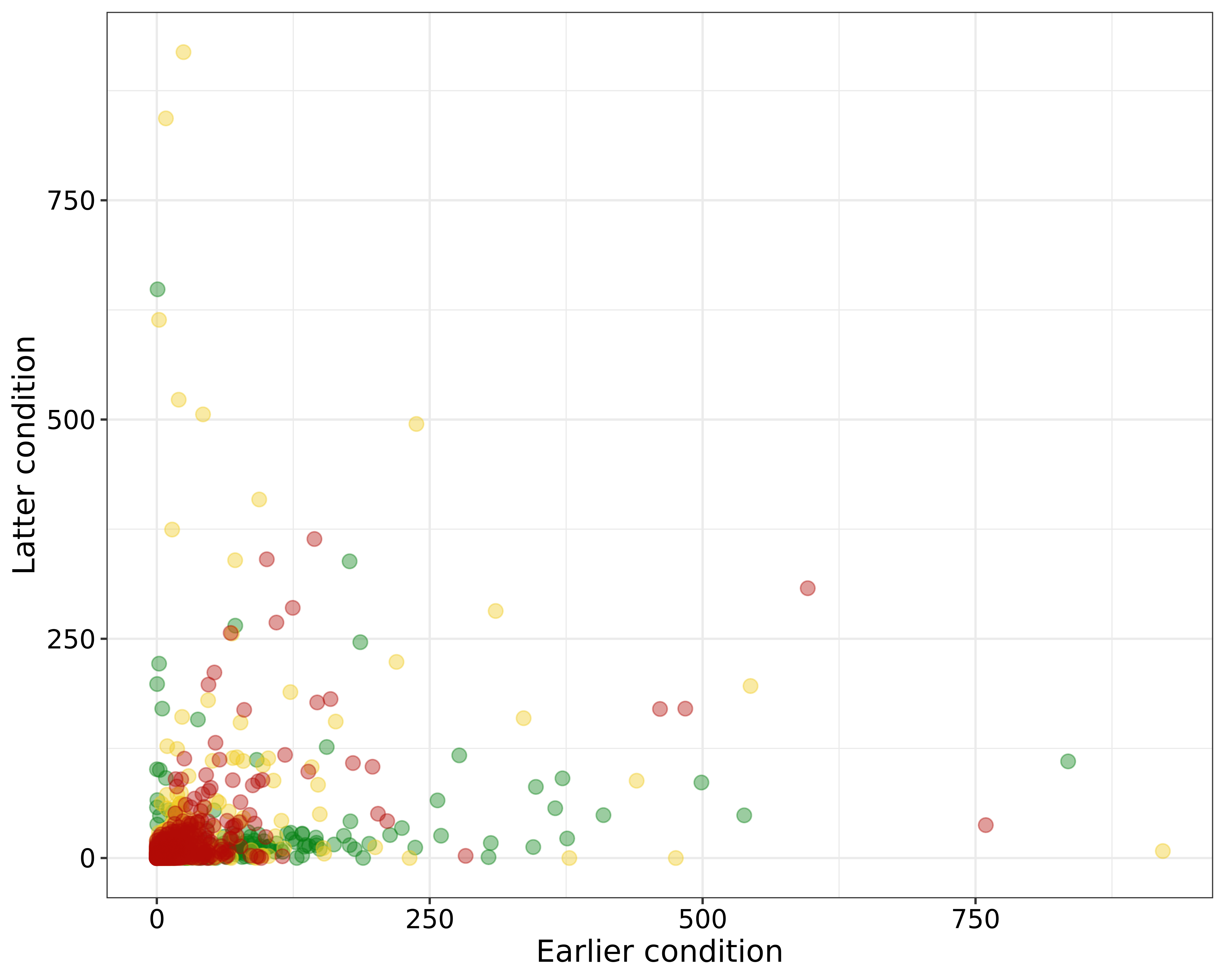

Supplement: Supplementary file 1 [file cells-09-00779-s001.zip › Supplementary materials/FigS11/2.tif]

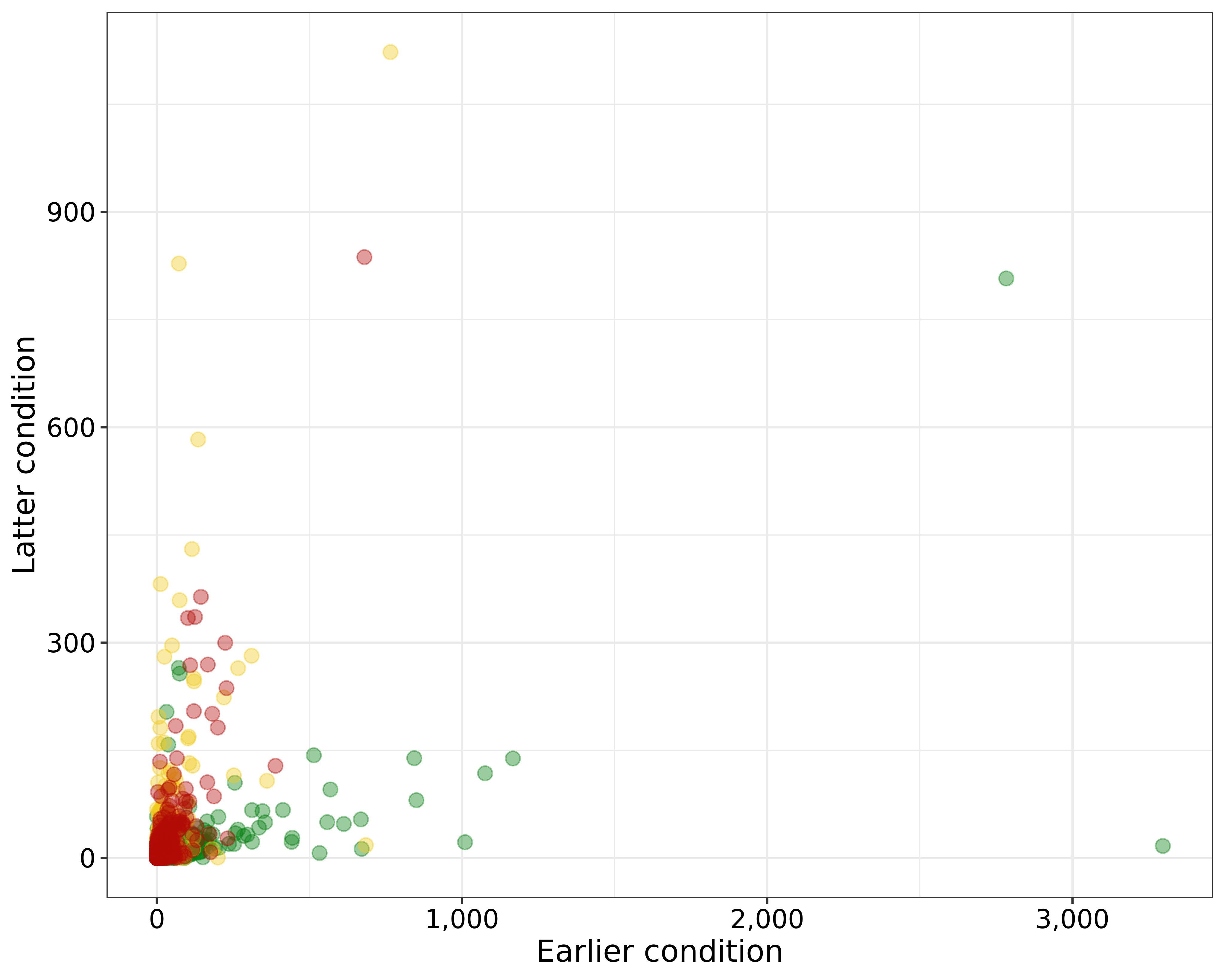

Supplement: Supplementary file 1 [file cells-09-00779-s001.zip › Supplementary materials/FigS11/20.tif]

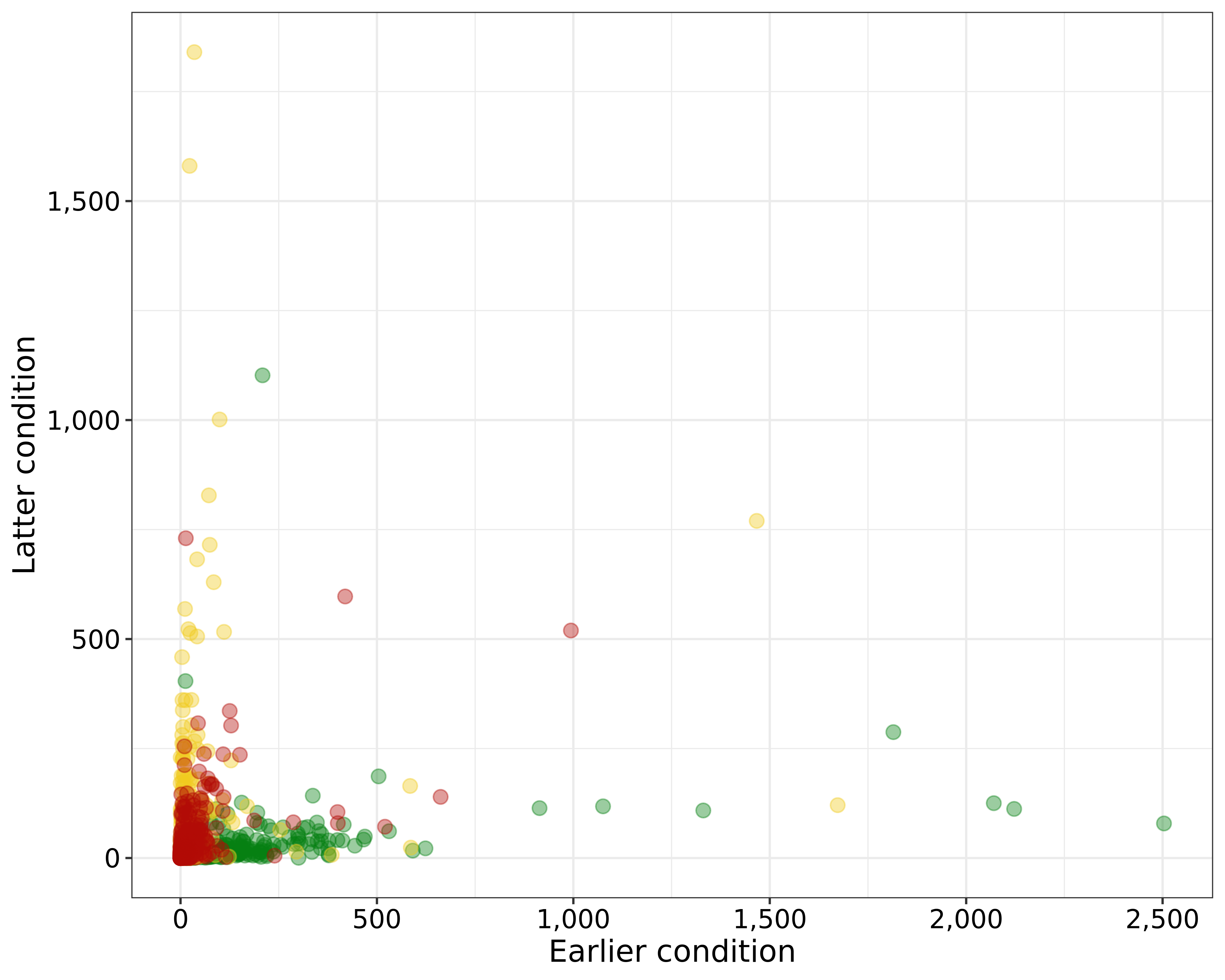

Supplement: Supplementary file 1 [file cells-09-00779-s001.zip › Supplementary materials/FigS11/21.tif]

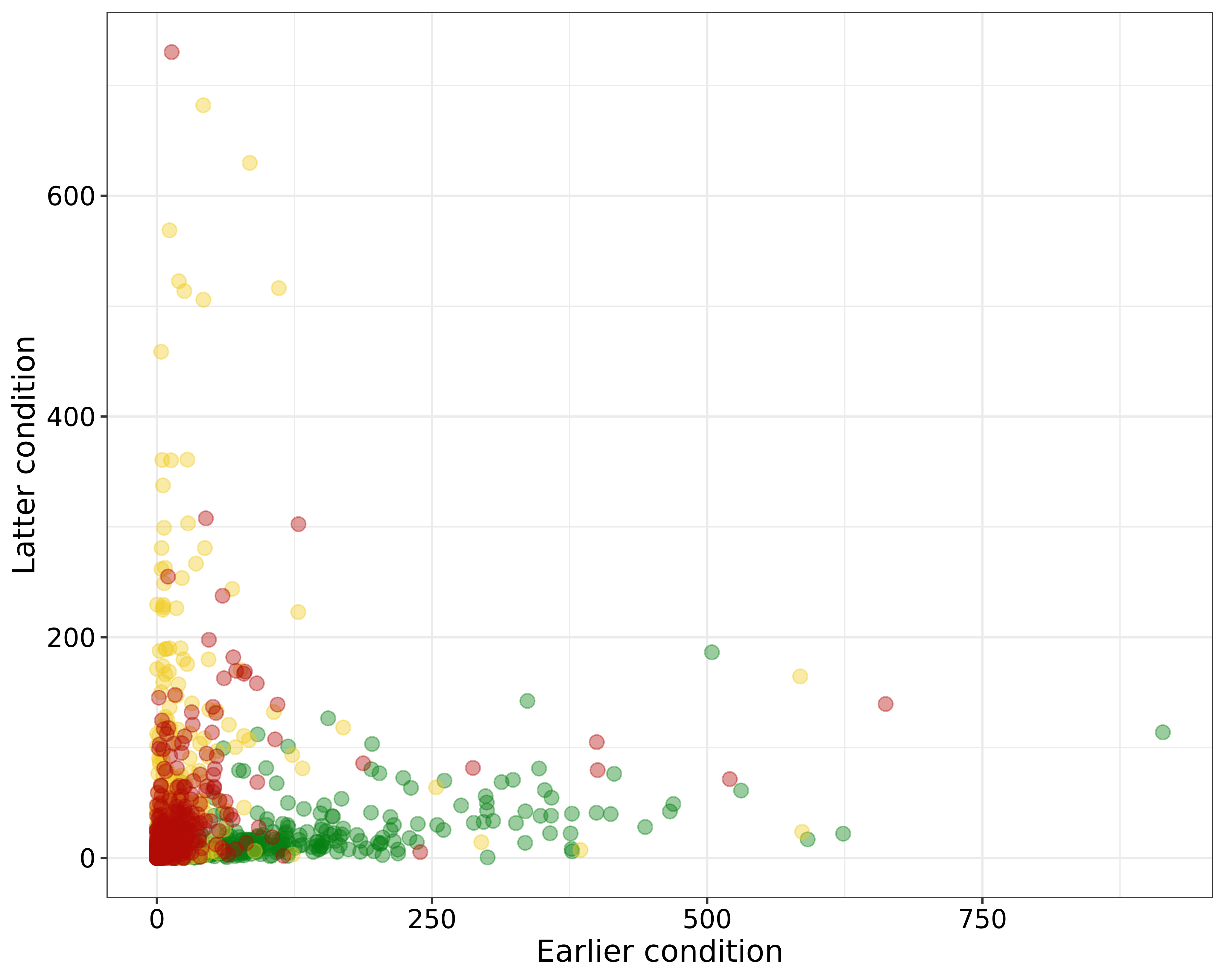

Supplement: Supplementary file 1 [file cells-09-00779-s001.zip › Supplementary materials/FigS11/22.tif]

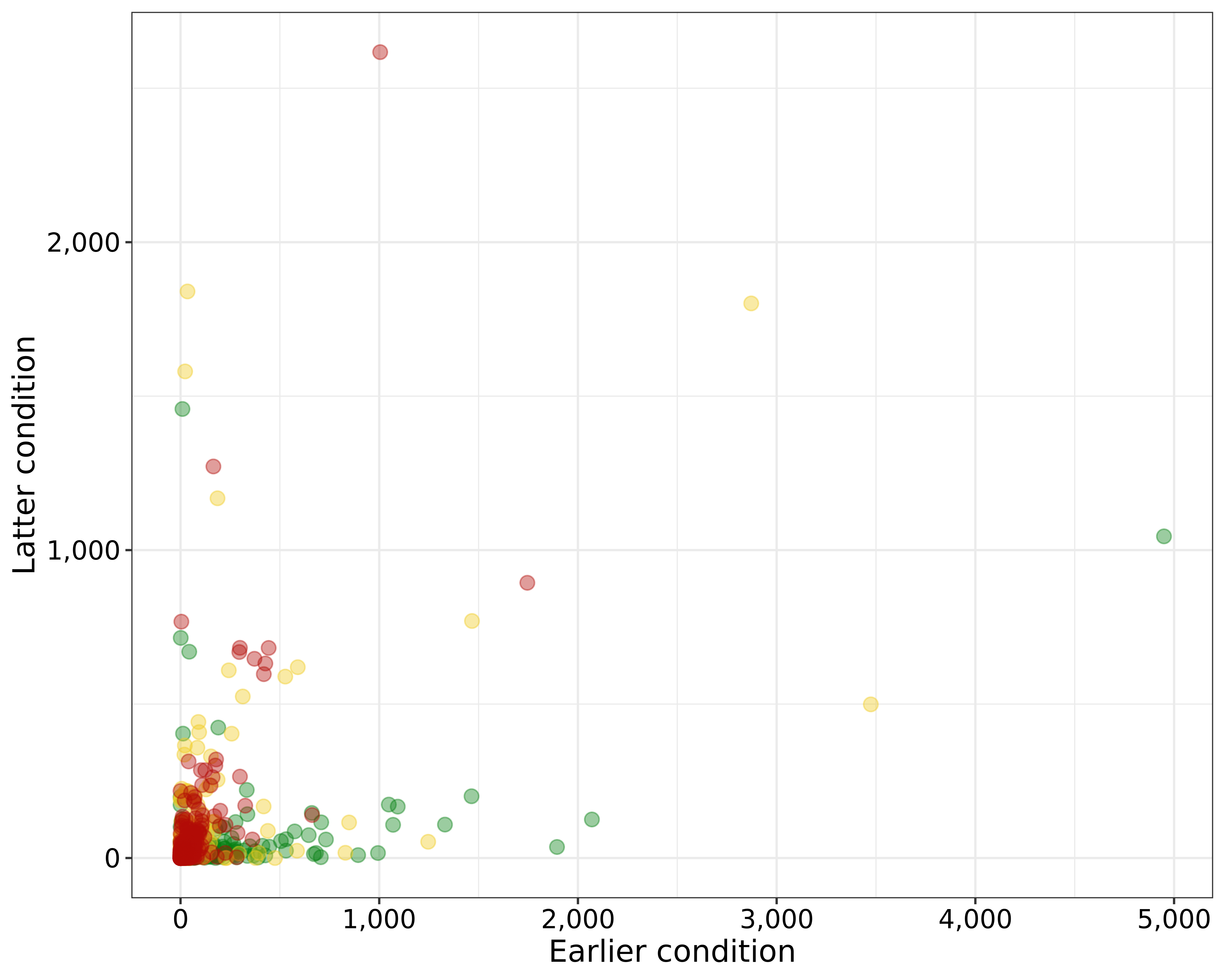

Supplement: Supplementary file 1 [file cells-09-00779-s001.zip › Supplementary materials/FigS11/23.tif]

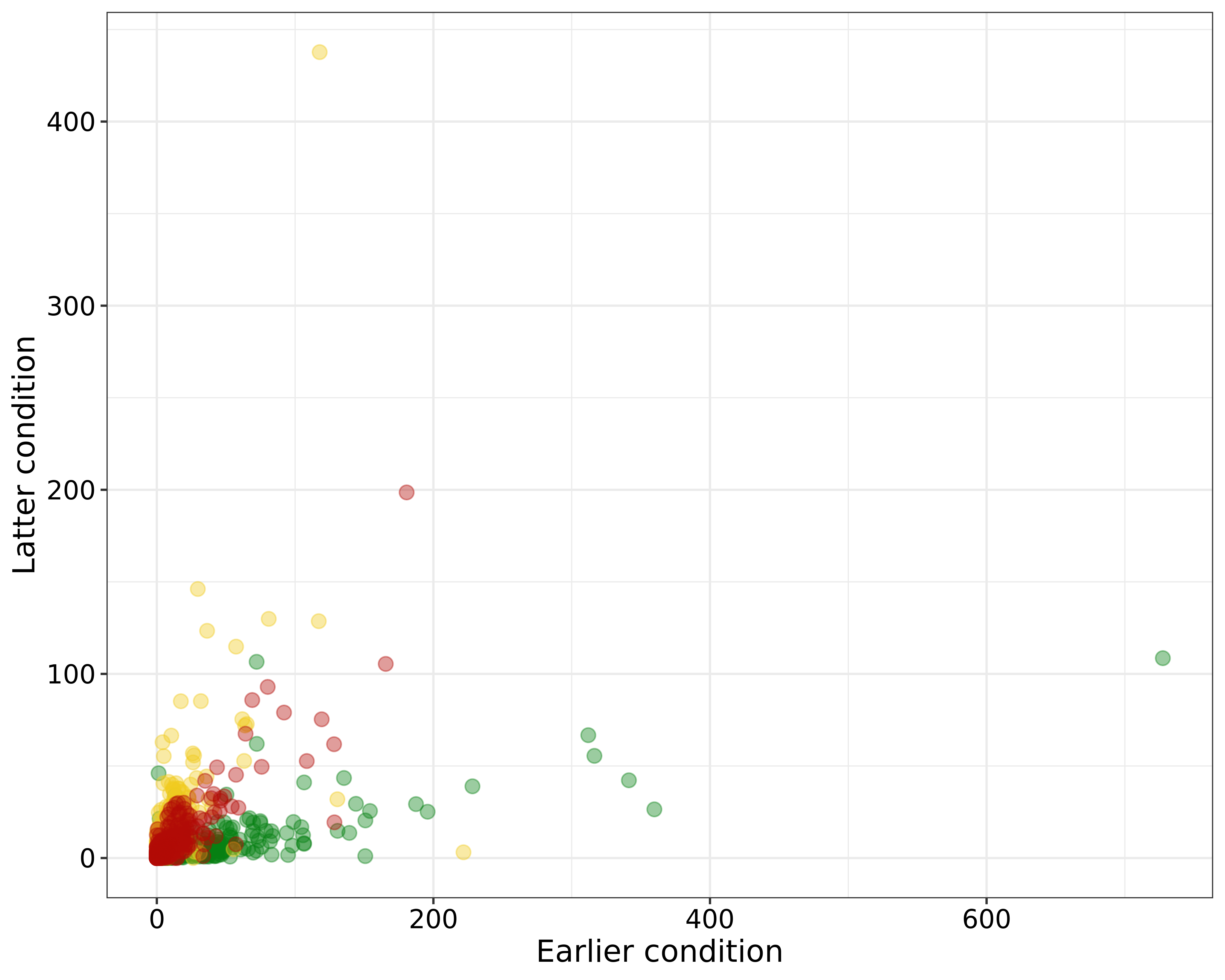

Supplement: Supplementary file 1 [file cells-09-00779-s001.zip › Supplementary materials/FigS11/24.tif]

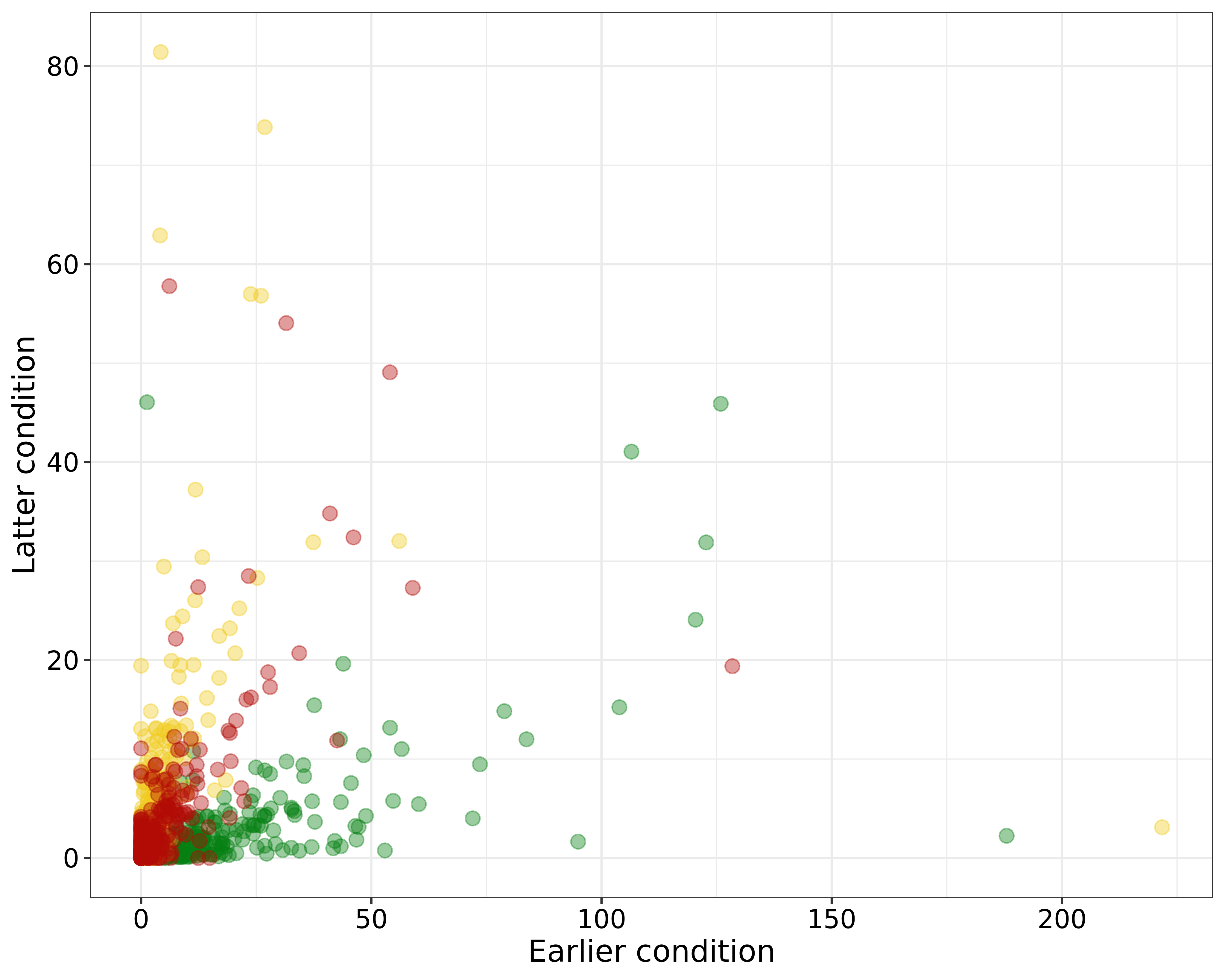

Supplement: Supplementary file 1 [file cells-09-00779-s001.zip › Supplementary materials/FigS11/25.tif]

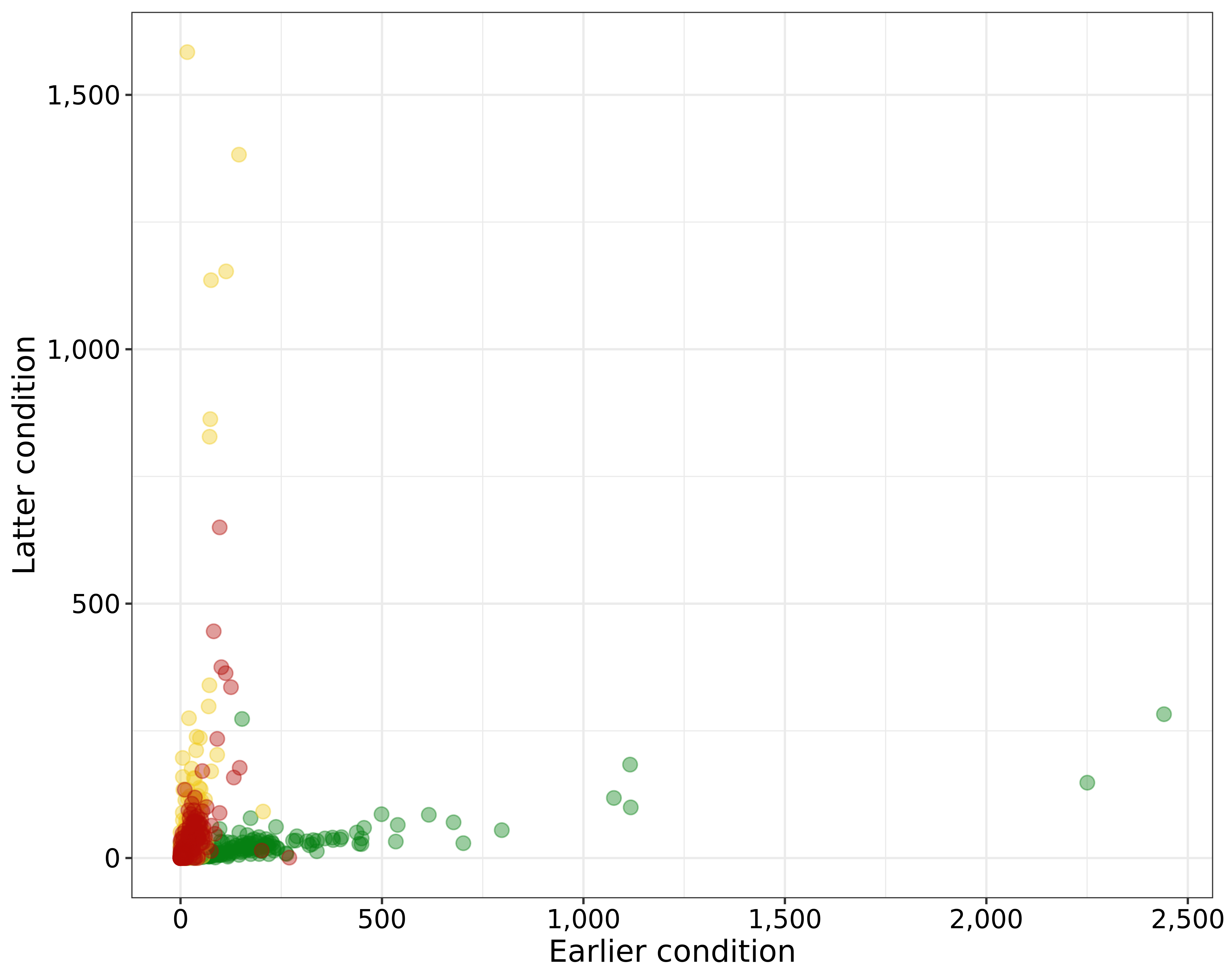

Supplement: Supplementary file 1 [file cells-09-00779-s001.zip › Supplementary materials/FigS11/26.tif]

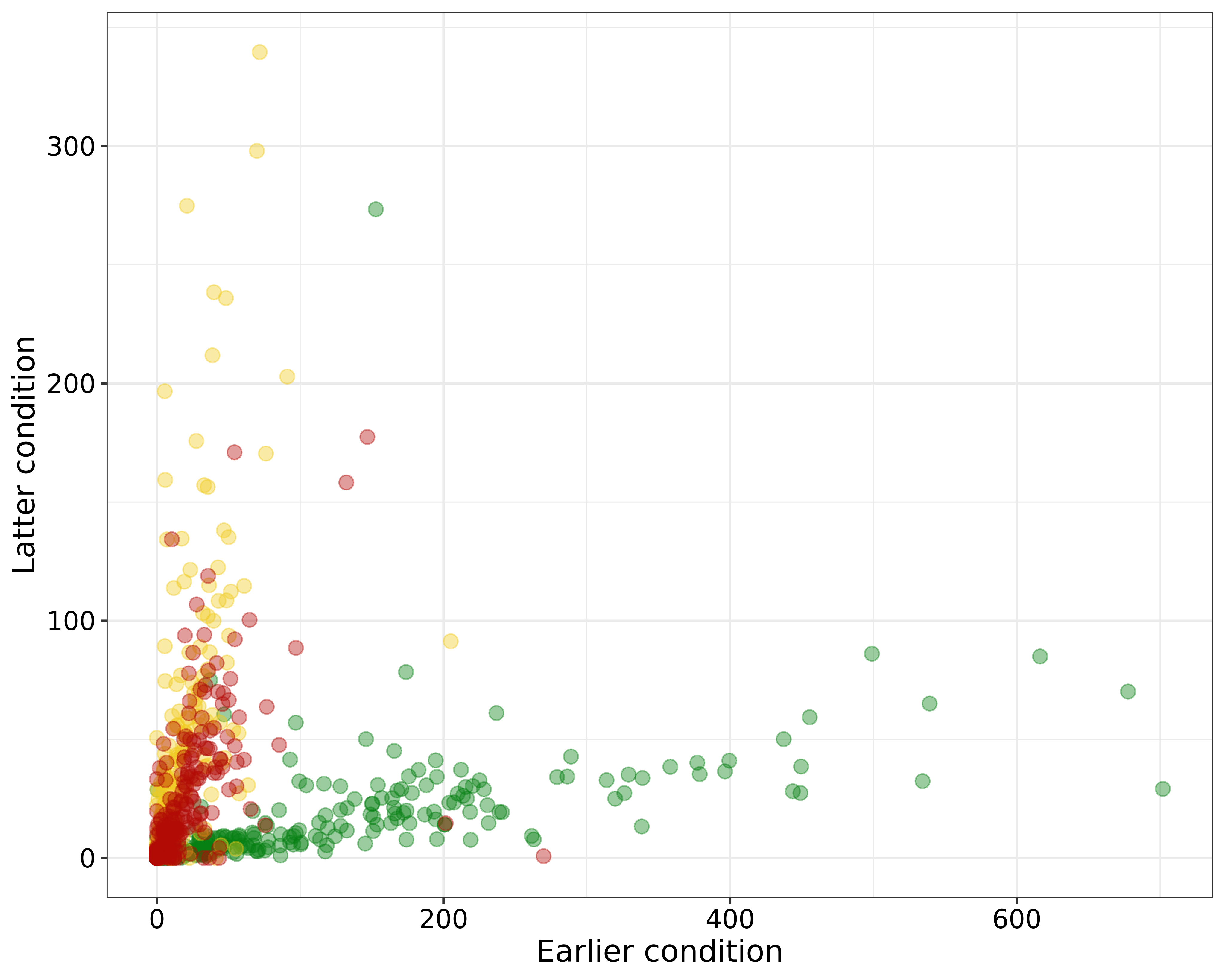

Supplement: Supplementary file 1 [file cells-09-00779-s001.zip › Supplementary materials/FigS11/27.tif]

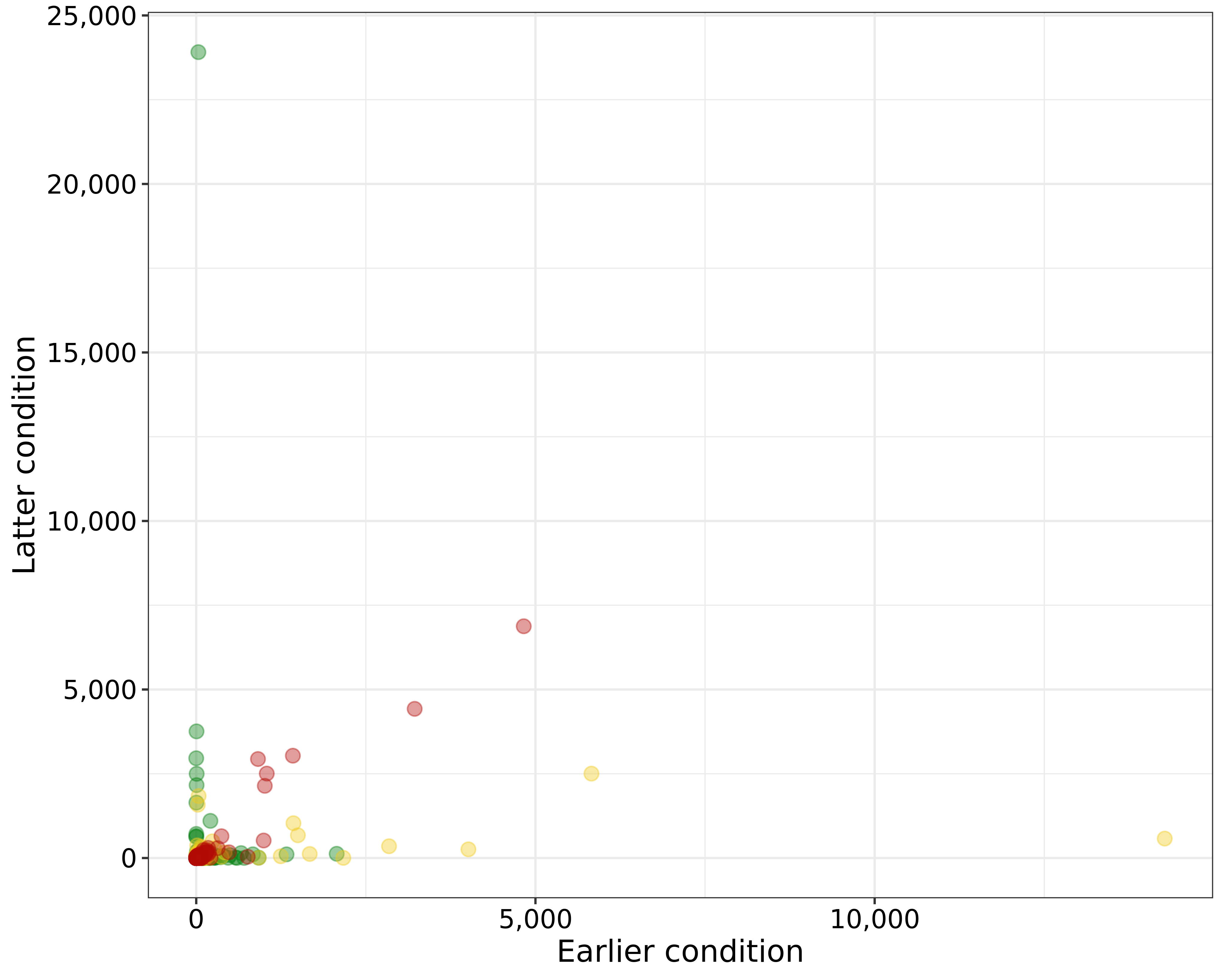

Supplement: Supplementary file 1 [file cells-09-00779-s001.zip › Supplementary materials/FigS11/28.tif]

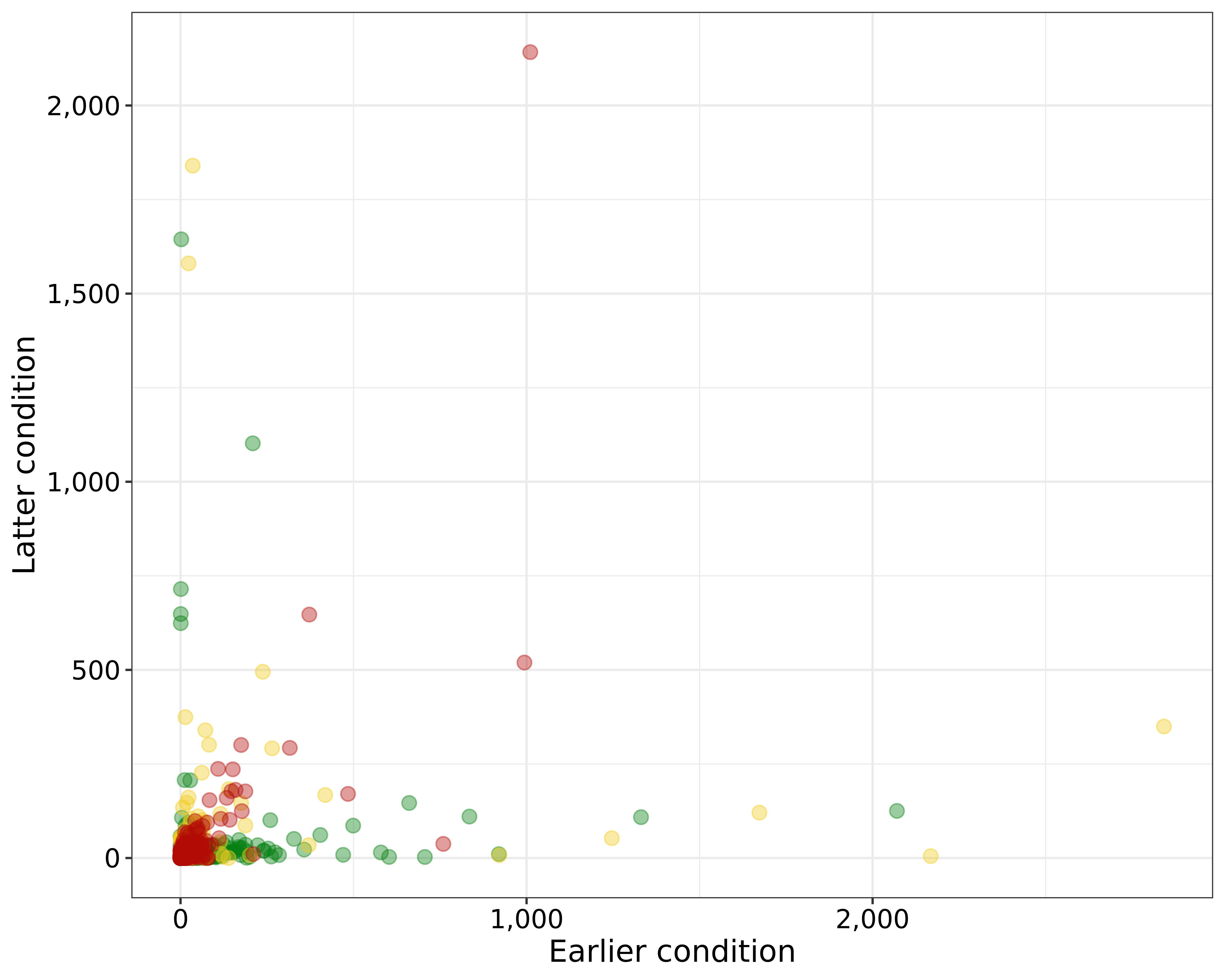

Supplement: Supplementary file 1 [file cells-09-00779-s001.zip › Supplementary materials/FigS11/29.tif]

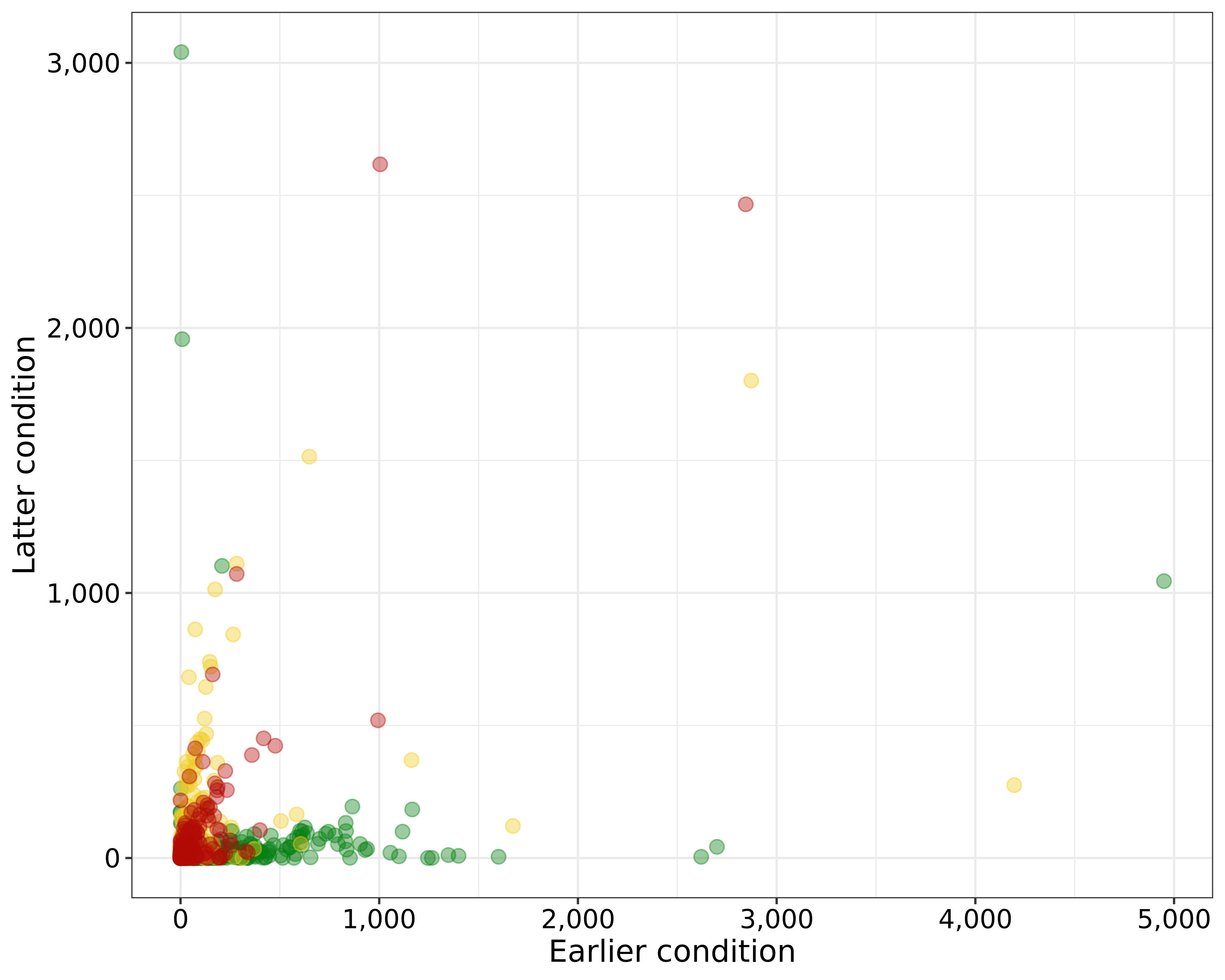

Supplement: Supplementary file 1 [file cells-09-00779-s001.zip › Supplementary materials/FigS11/3.tif]

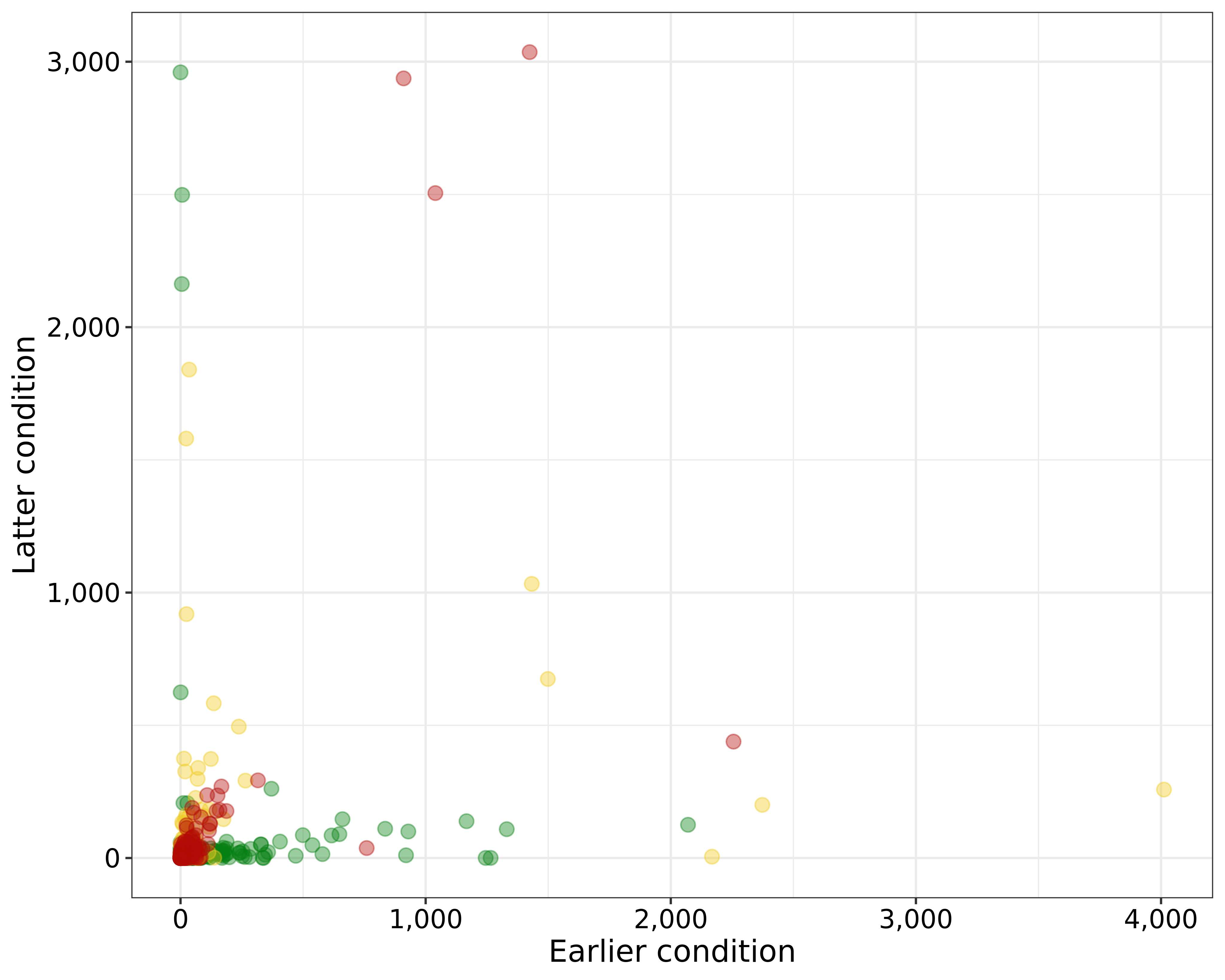

Supplement: Supplementary file 1 [file cells-09-00779-s001.zip › Supplementary materials/FigS11/30.tif]

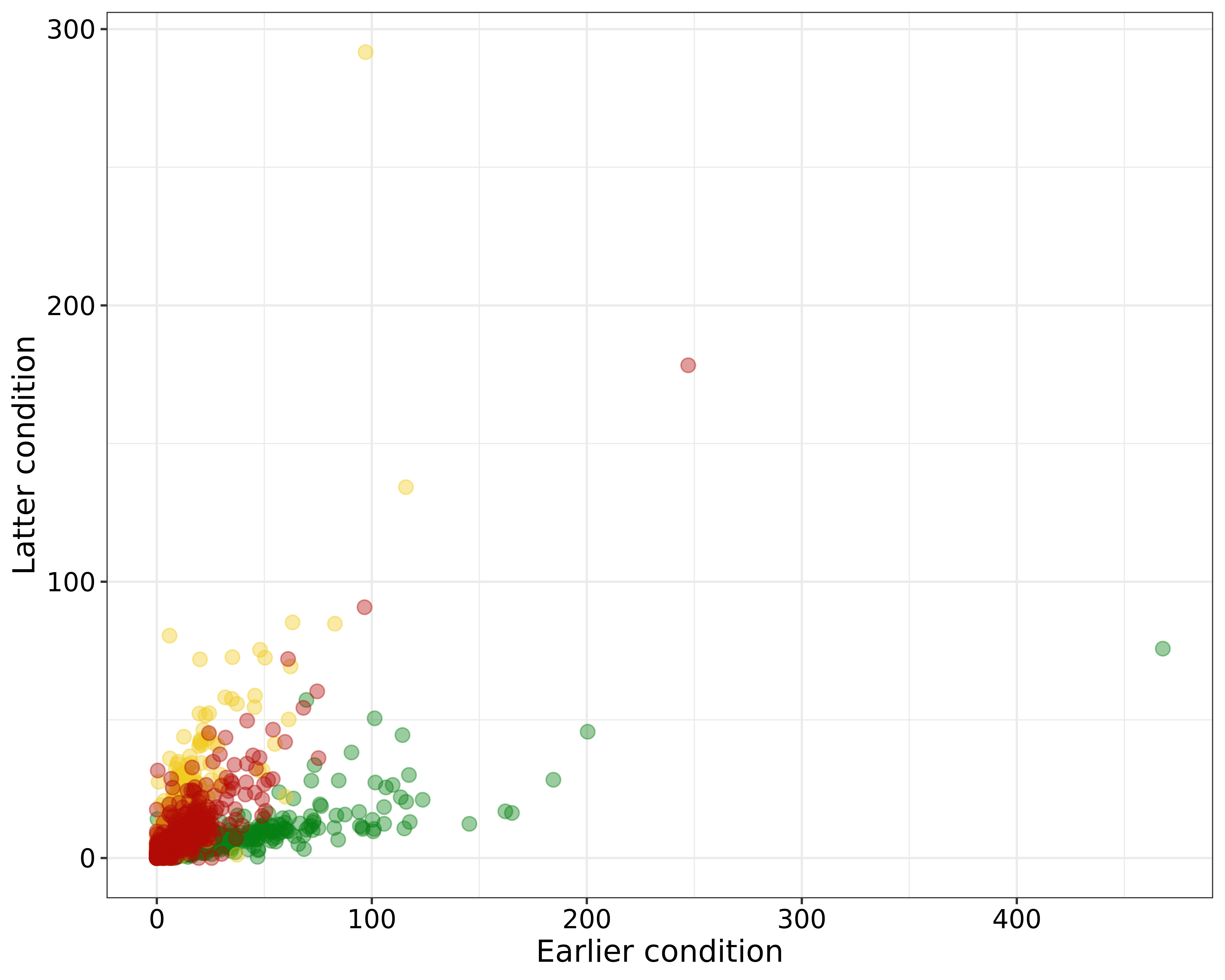

Supplement: Supplementary file 1 [file cells-09-00779-s001.zip › Supplementary materials/FigS11/31.tif]

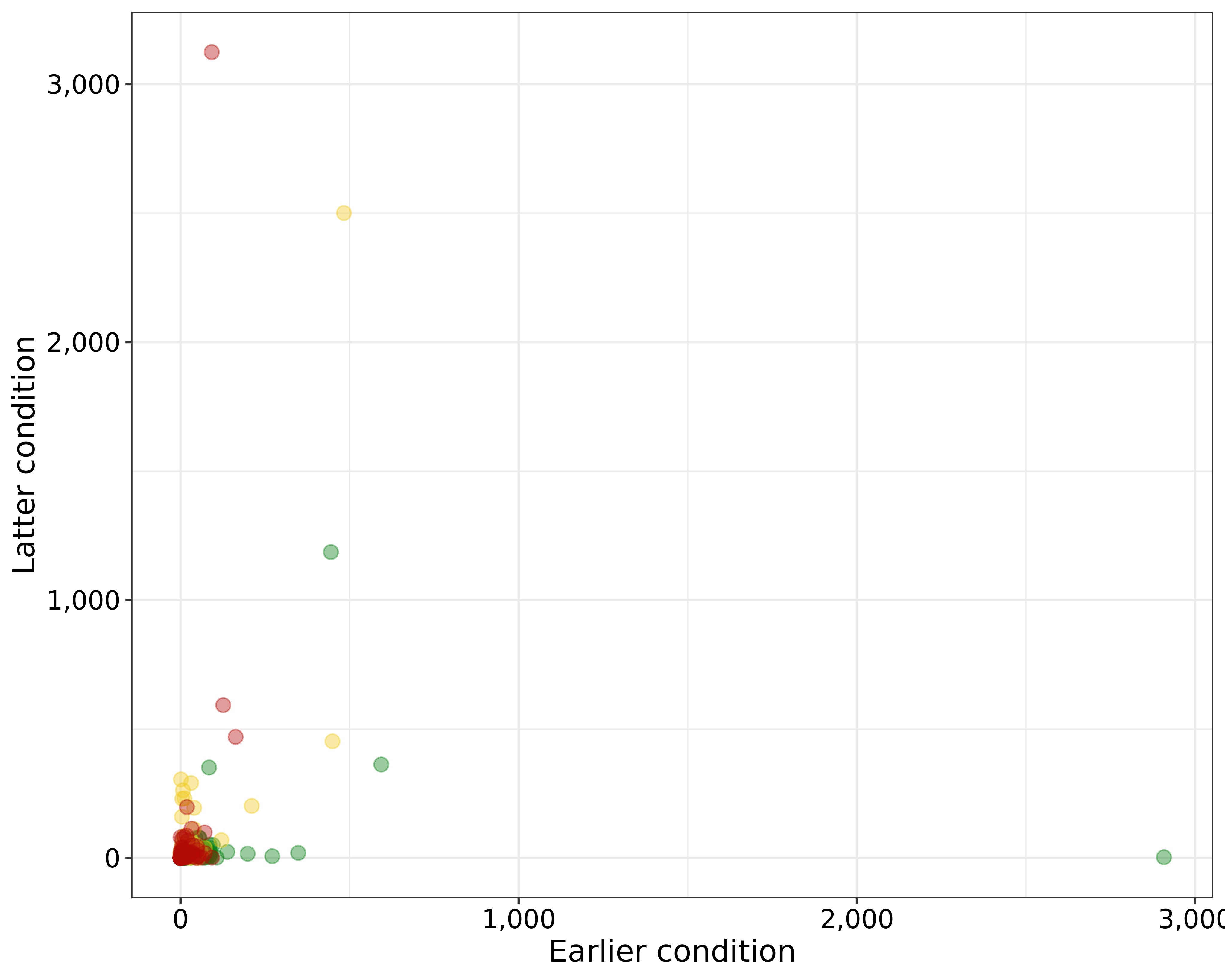

Supplement: Supplementary file 1 [file cells-09-00779-s001.zip › Supplementary materials/FigS11/32.tif]

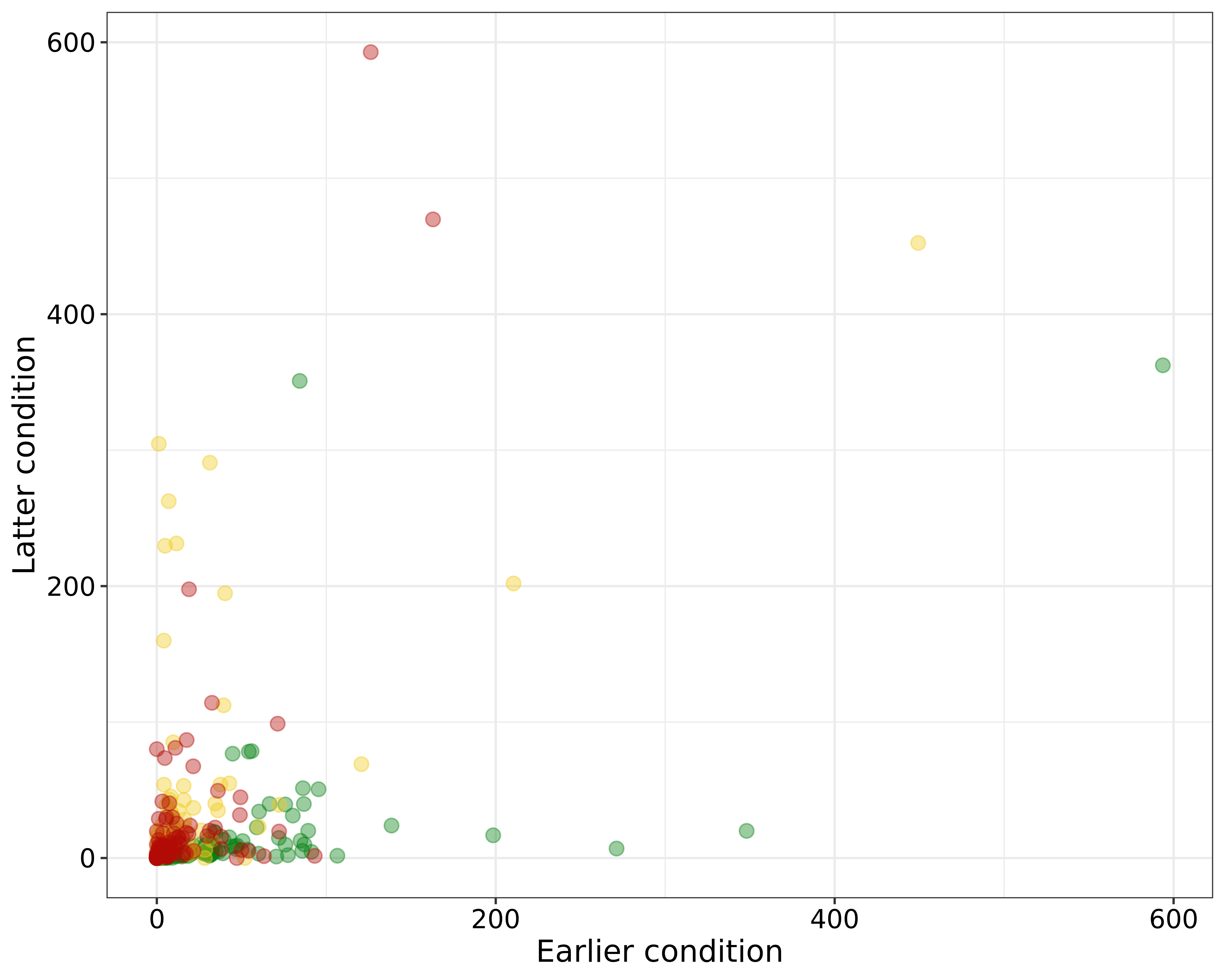

Supplement: Supplementary file 1 [file cells-09-00779-s001.zip › Supplementary materials/FigS11/33.tif]

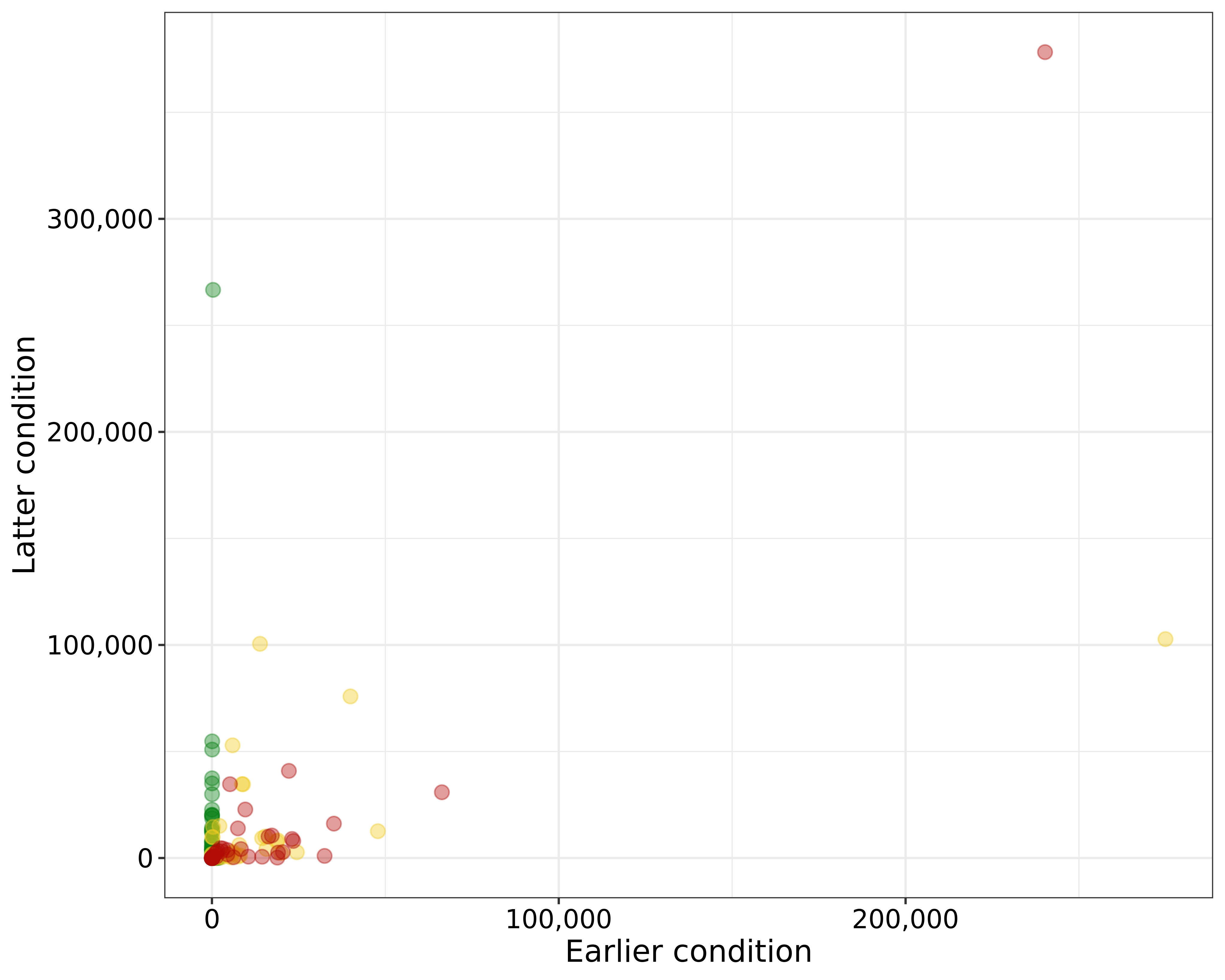

Supplement: Supplementary file 1 [file cells-09-00779-s001.zip › Supplementary materials/FigS11/34.tif]

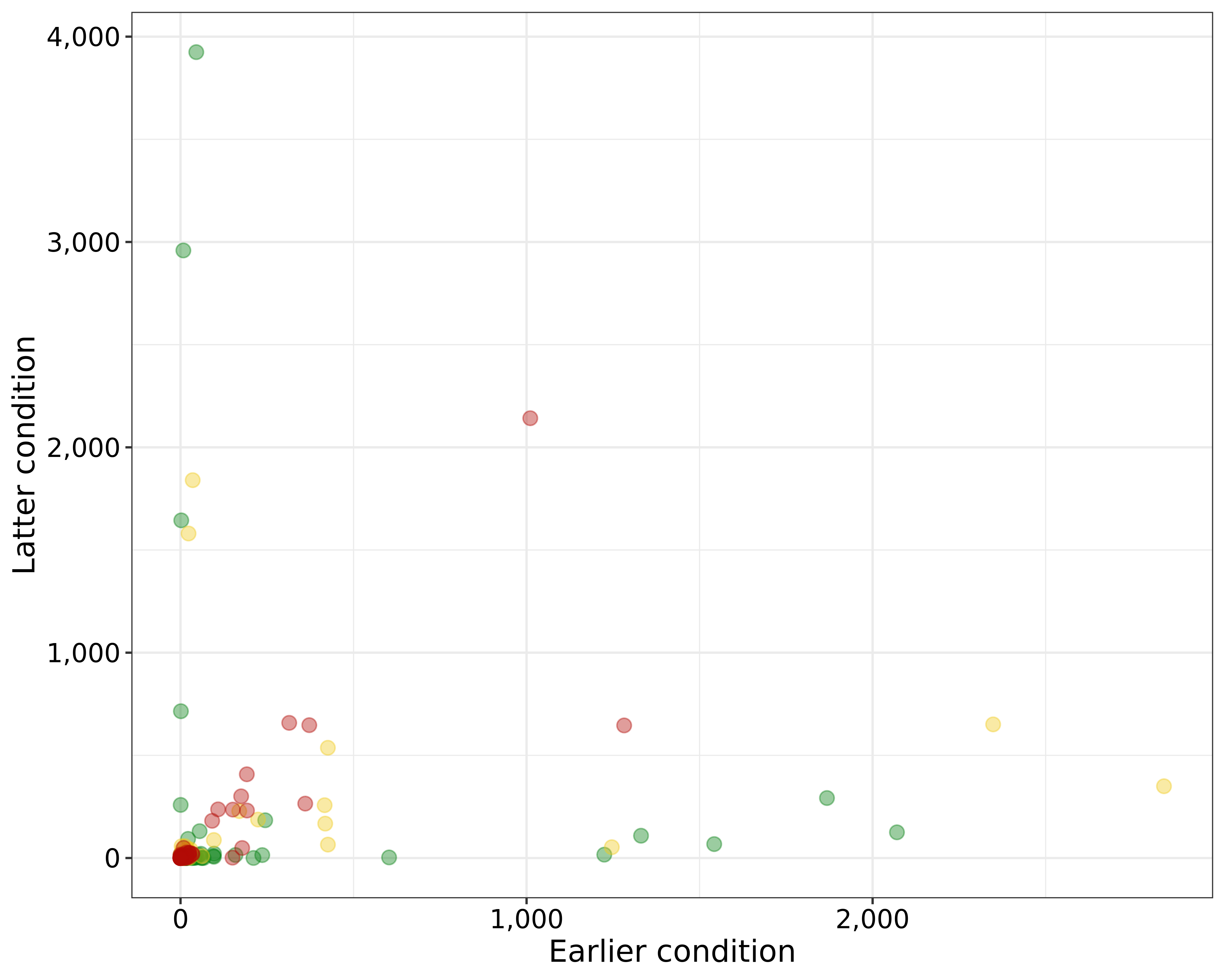

Supplement: Supplementary file 1 [file cells-09-00779-s001.zip › Supplementary materials/FigS11/35.tif]

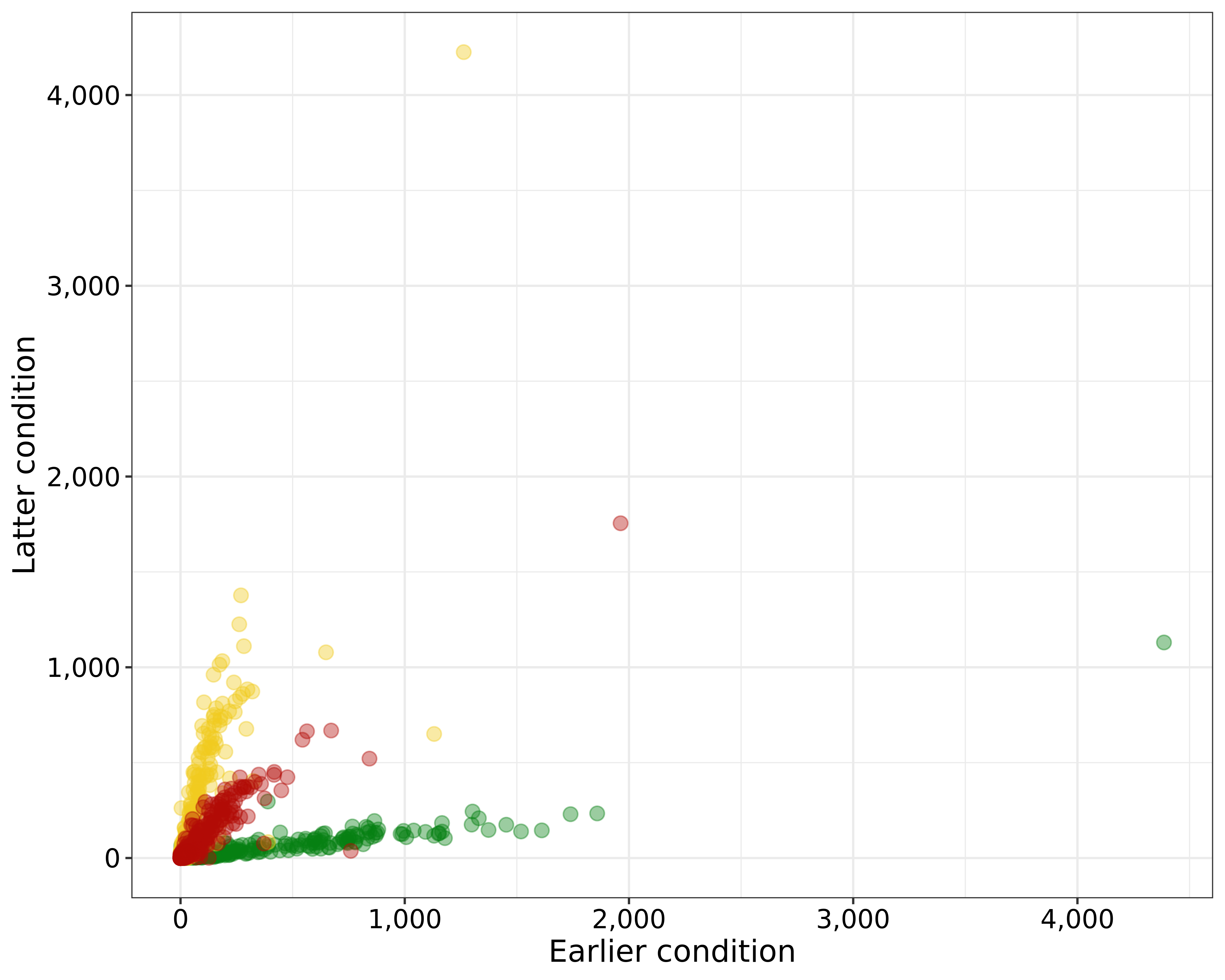

Supplement: Supplementary file 1 [file cells-09-00779-s001.zip › Supplementary materials/FigS11/36.tif]

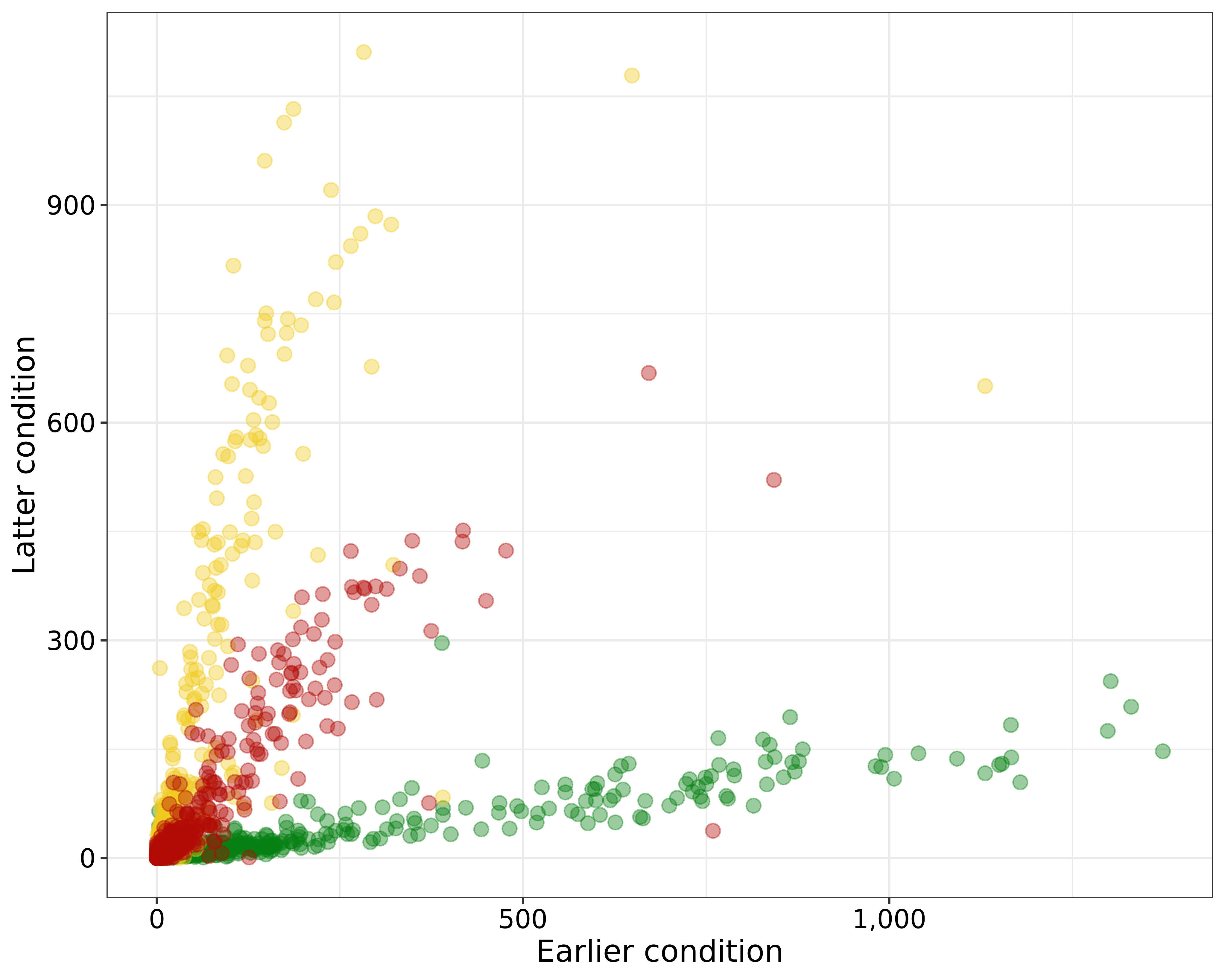

Supplement: Supplementary file 1 [file cells-09-00779-s001.zip › Supplementary materials/FigS11/37.tif]

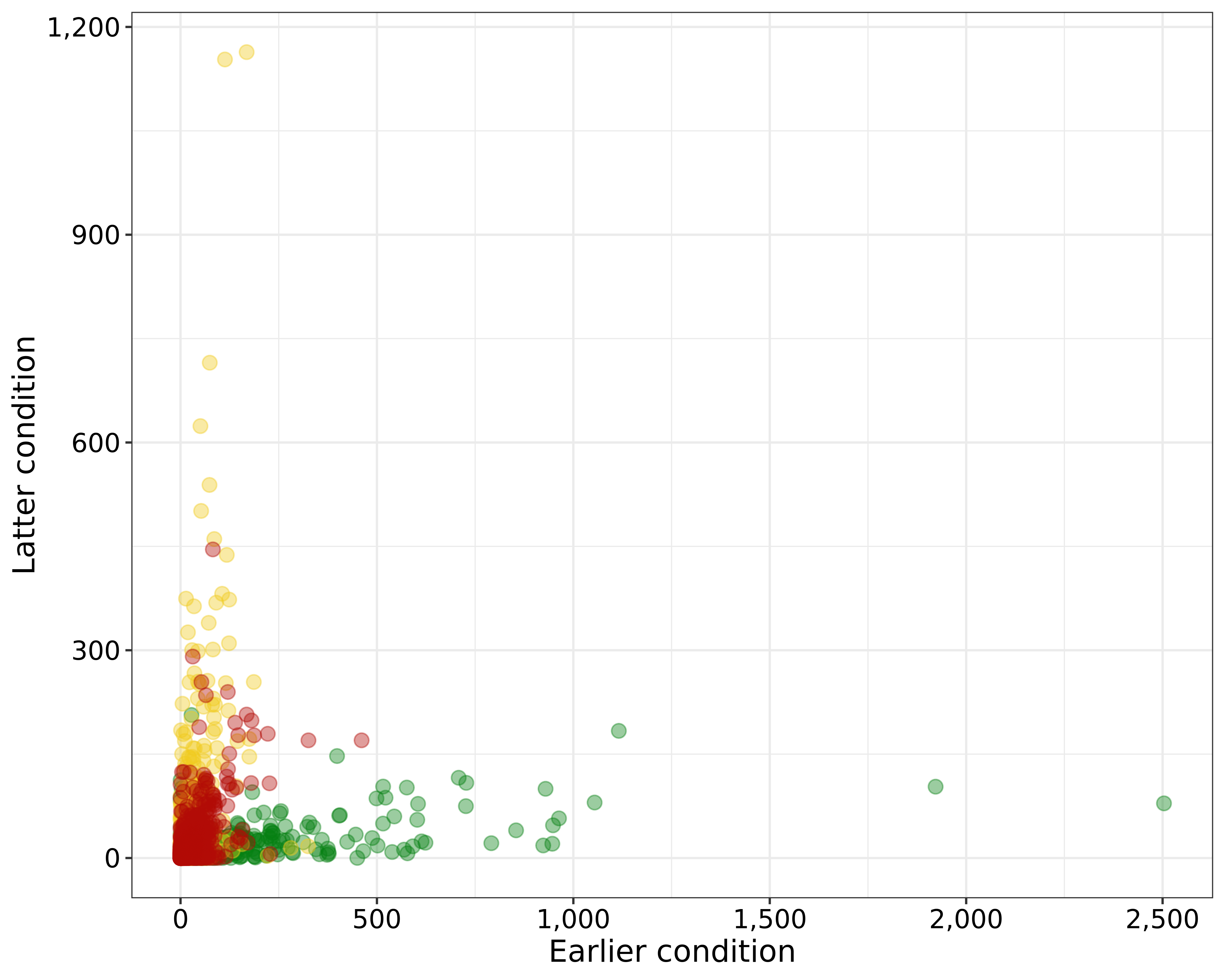

Supplement: Supplementary file 1 [file cells-09-00779-s001.zip › Supplementary materials/FigS11/38.tif]

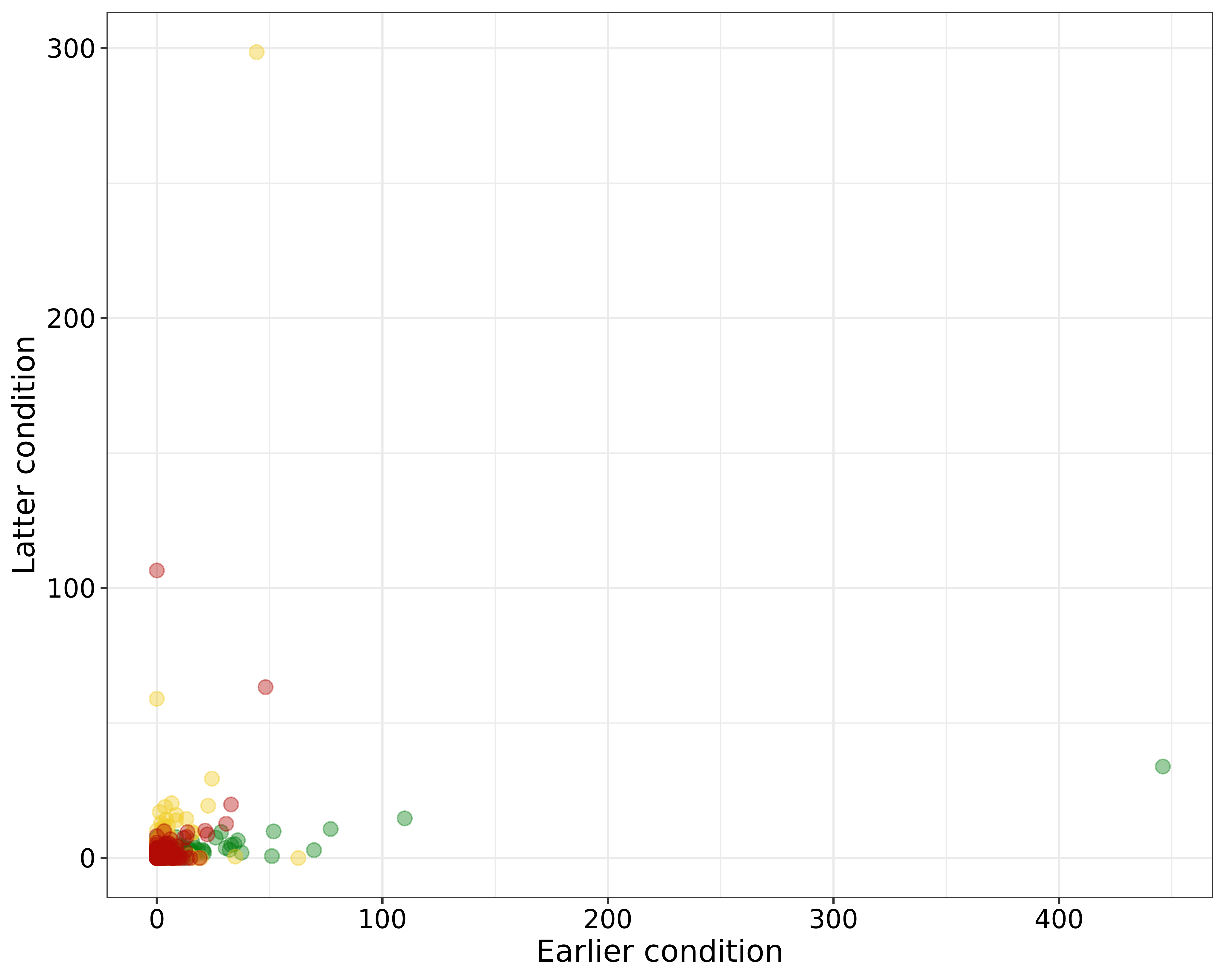

Supplement: Supplementary file 1 [file cells-09-00779-s001.zip › Supplementary materials/FigS11/39.tif]

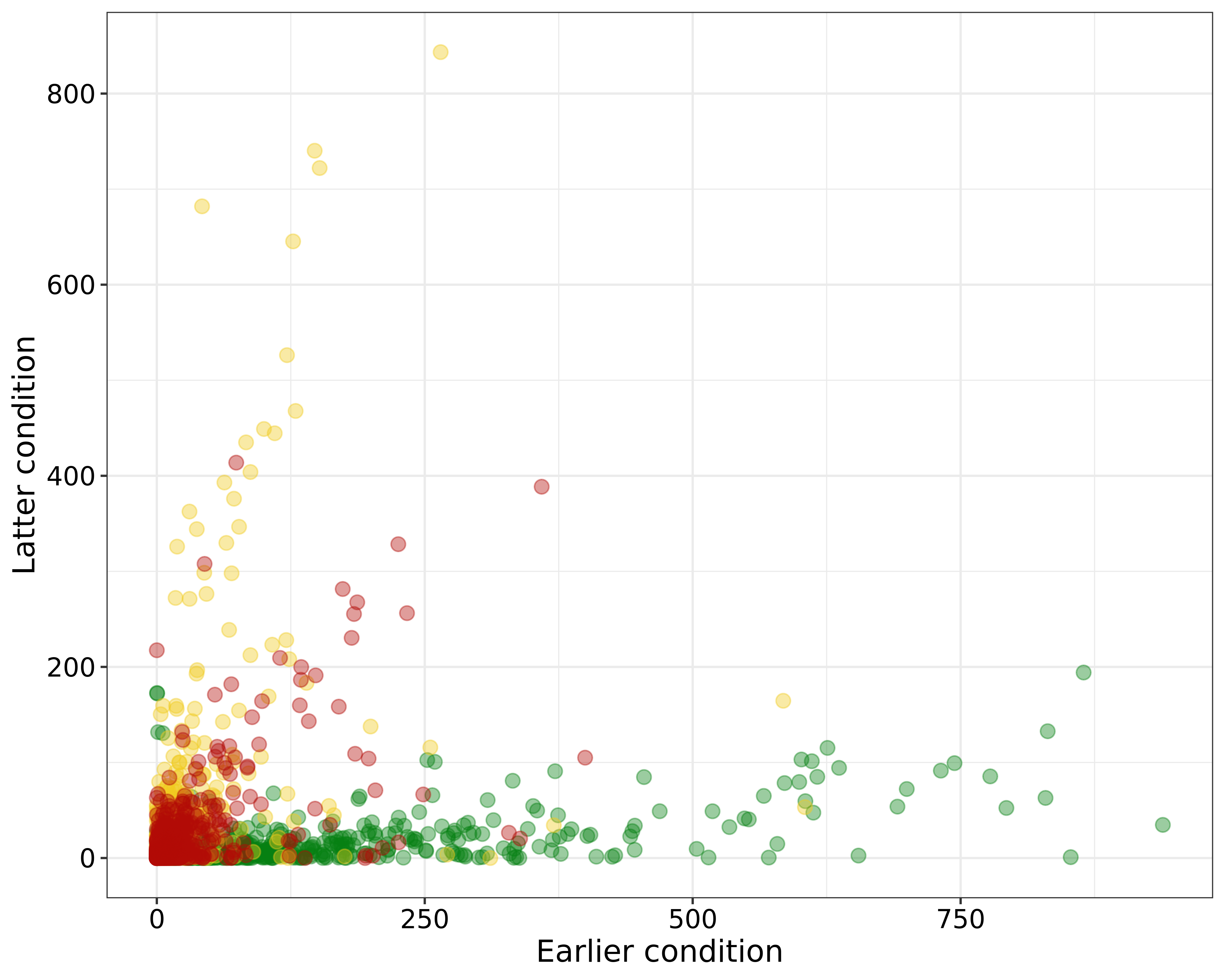

Supplement: Supplementary file 1 [file cells-09-00779-s001.zip › Supplementary materials/FigS11/4.tif]

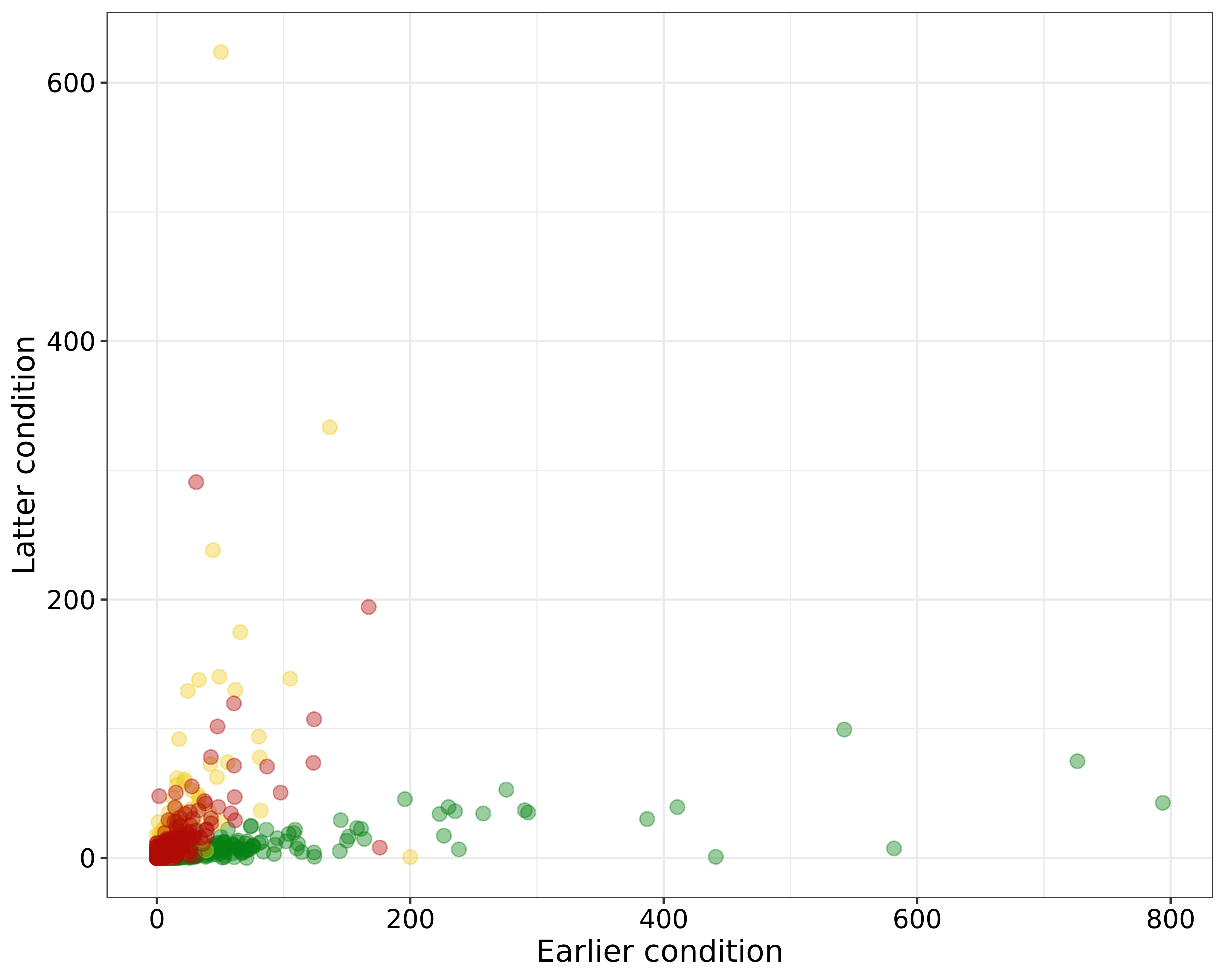

Supplement: Supplementary file 1 [file cells-09-00779-s001.zip › Supplementary materials/FigS11/40.tif]

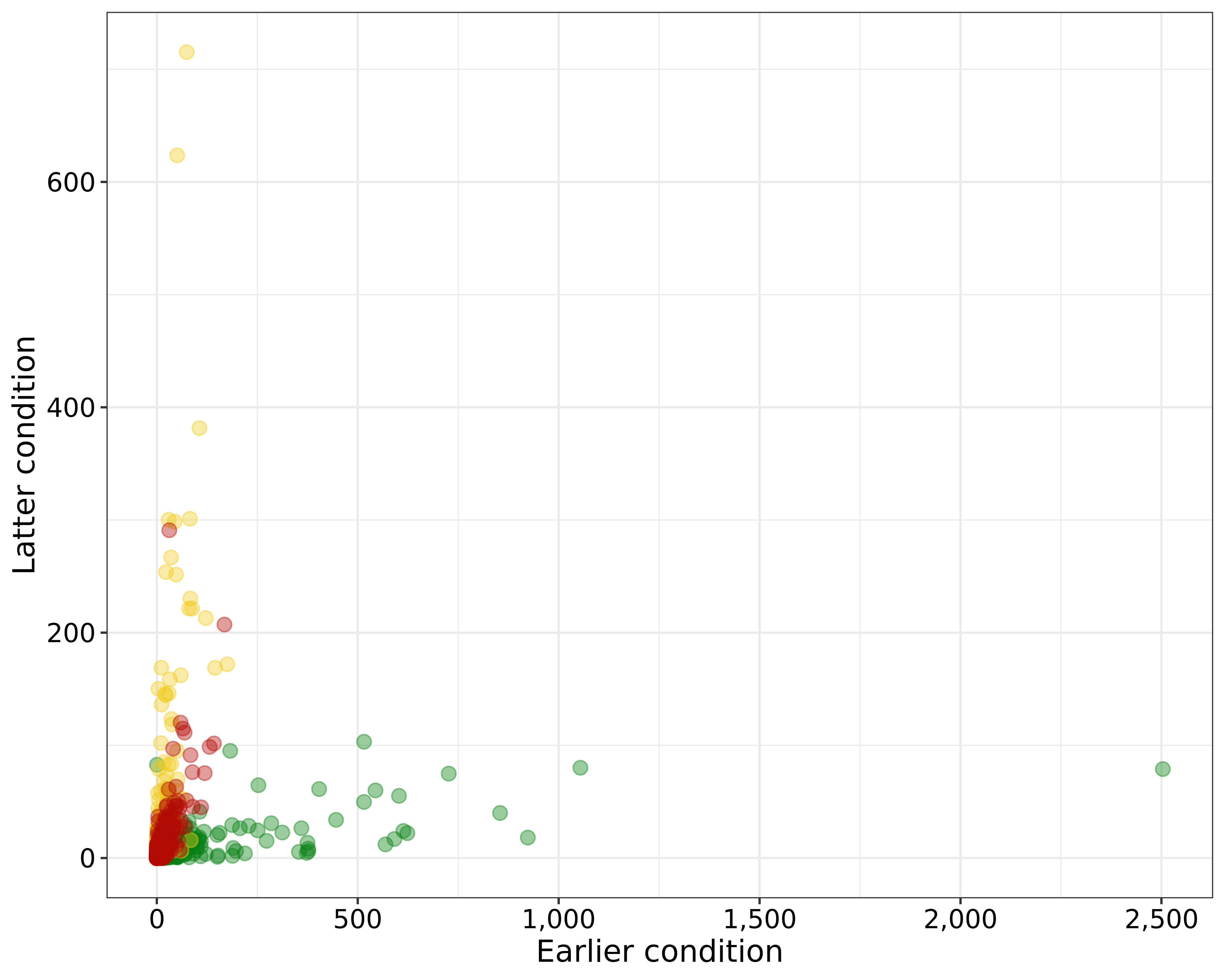

Supplement: Supplementary file 1 [file cells-09-00779-s001.zip › Supplementary materials/FigS11/5.tif]

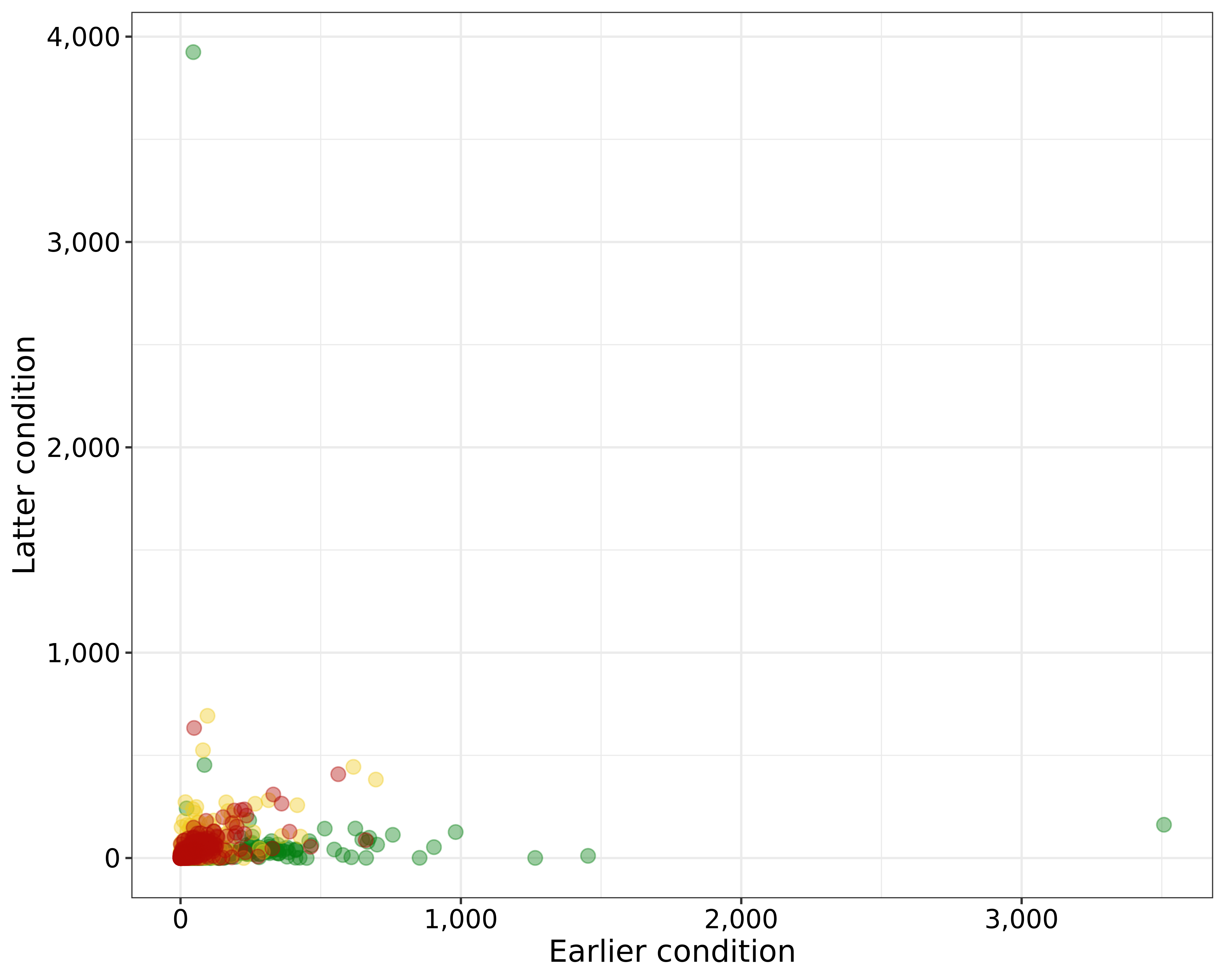

Supplement: Supplementary file 1 [file cells-09-00779-s001.zip › Supplementary materials/FigS11/6.tif]

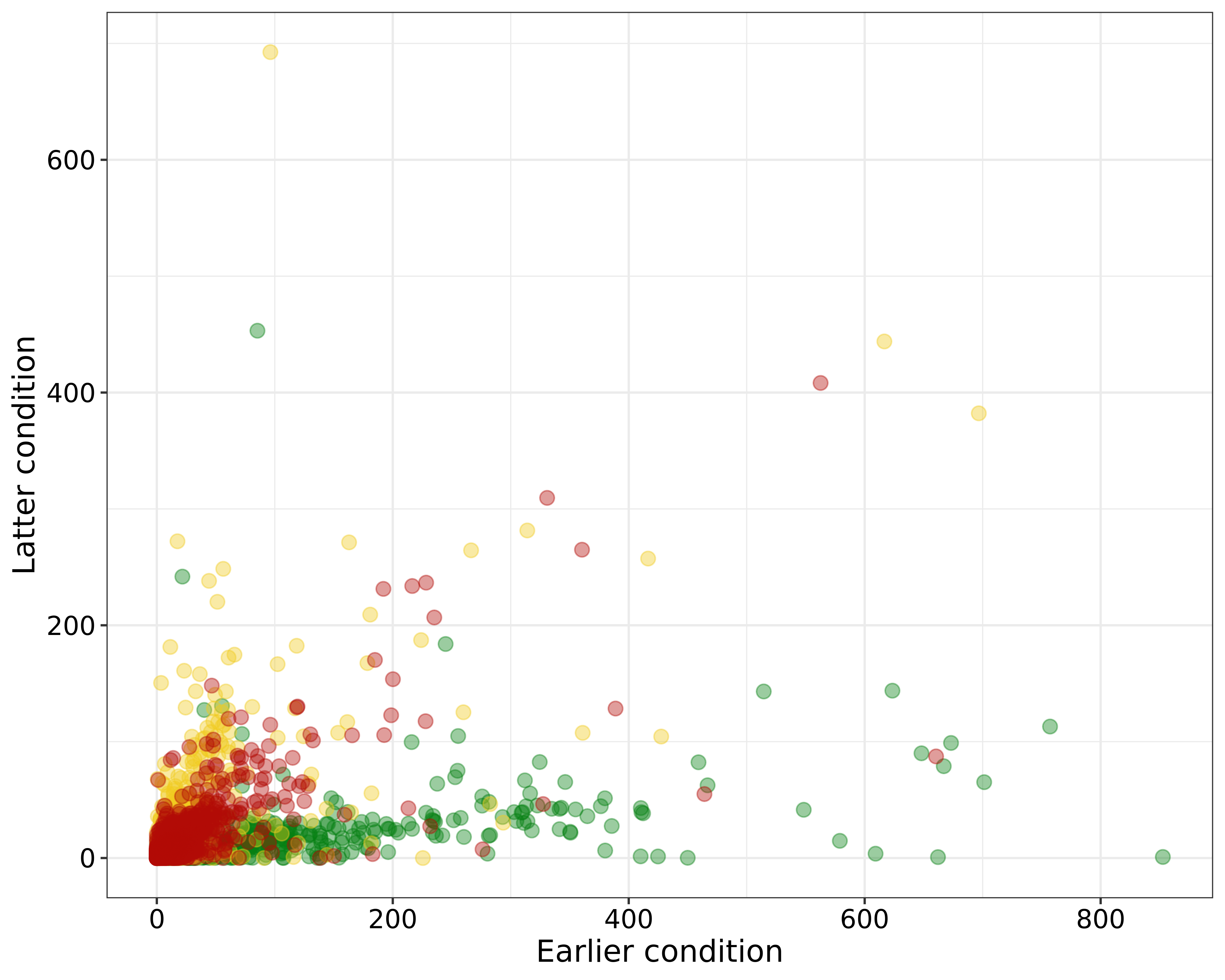

Supplement: Supplementary file 1 [file cells-09-00779-s001.zip › Supplementary materials/FigS11/7.tif]

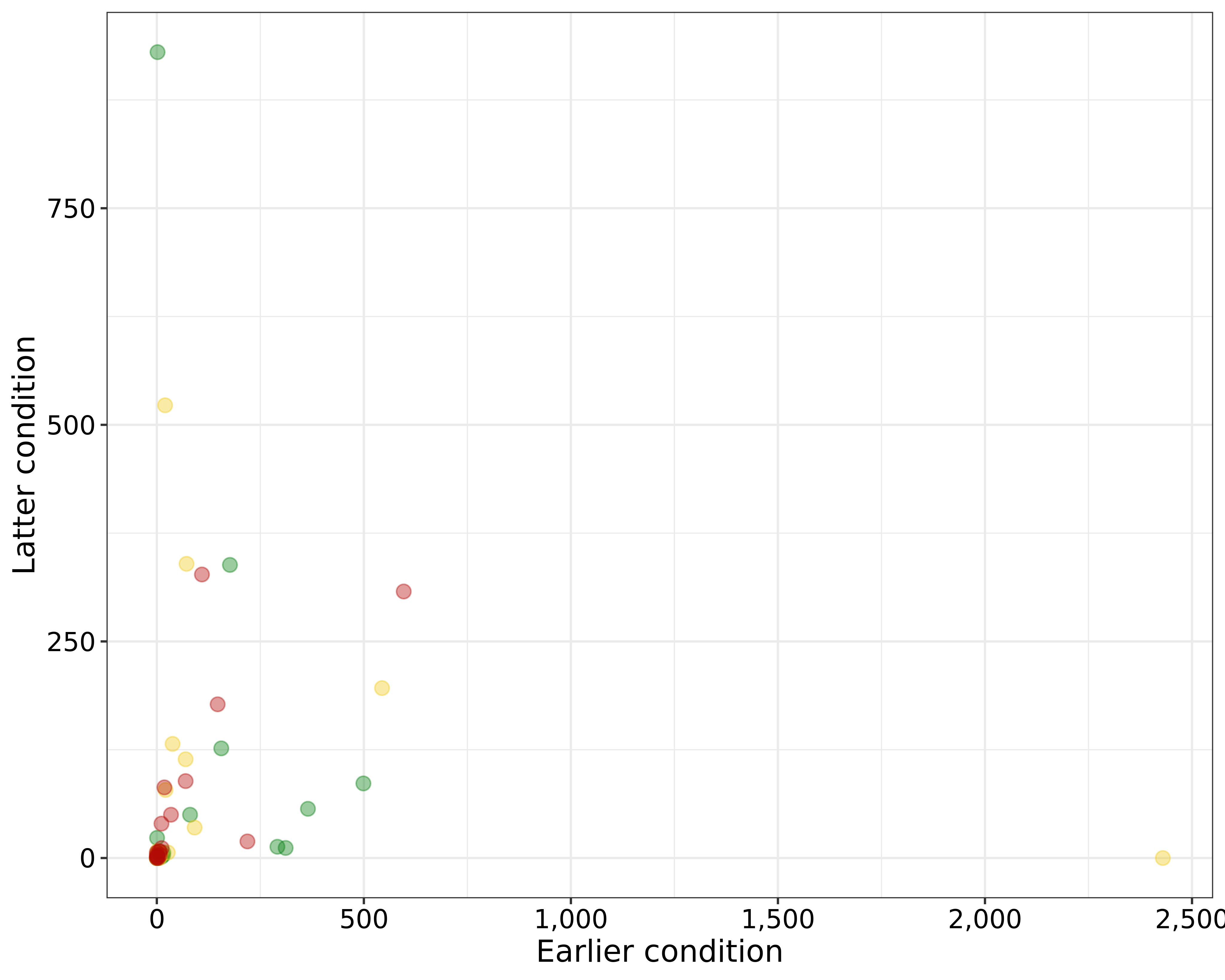

Supplement: Supplementary file 1 [file cells-09-00779-s001.zip › Supplementary materials/FigS11/8.tif]

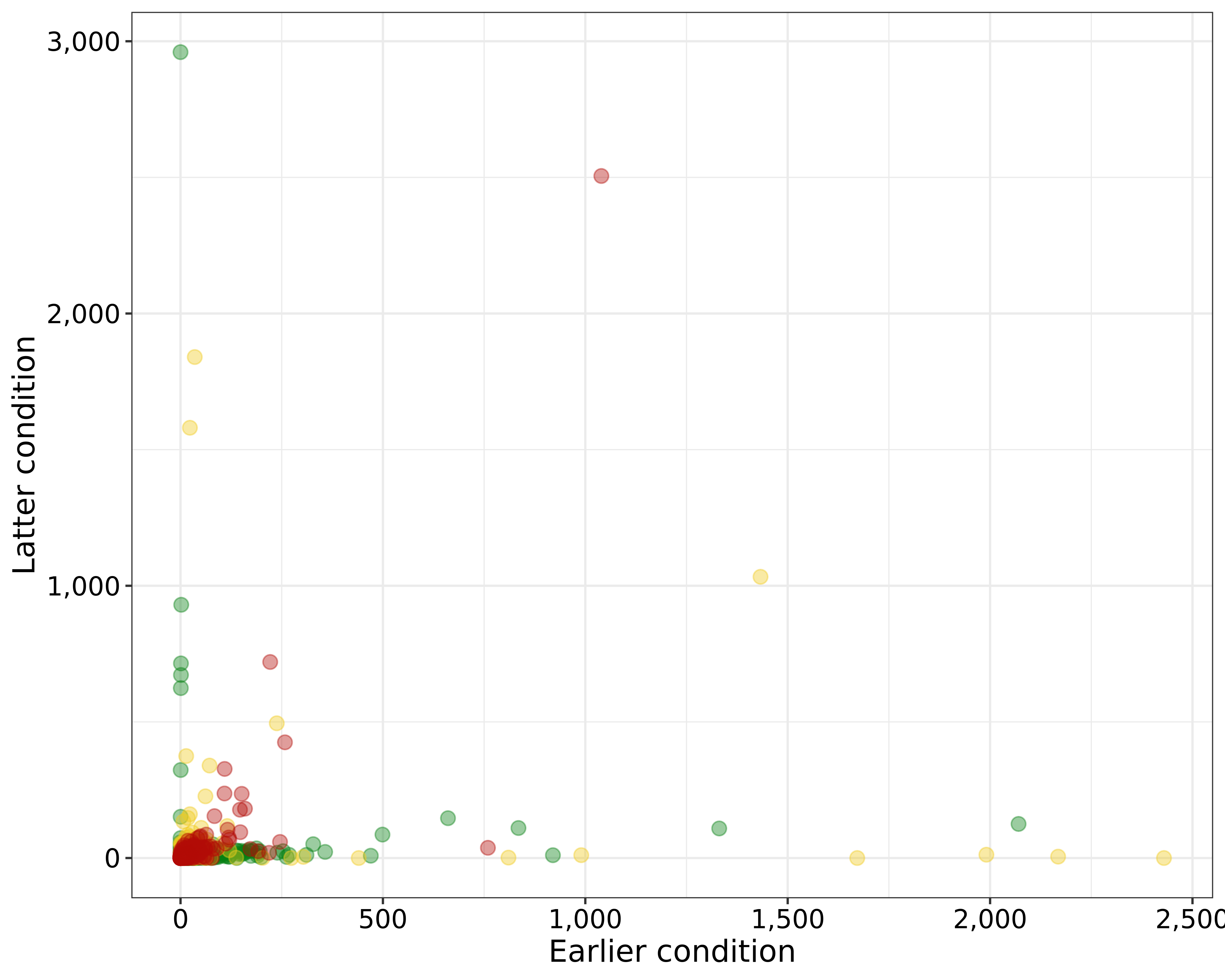

Supplement: Supplementary file 1 [file cells-09-00779-s001.zip › Supplementary materials/FigS11/9.tif]

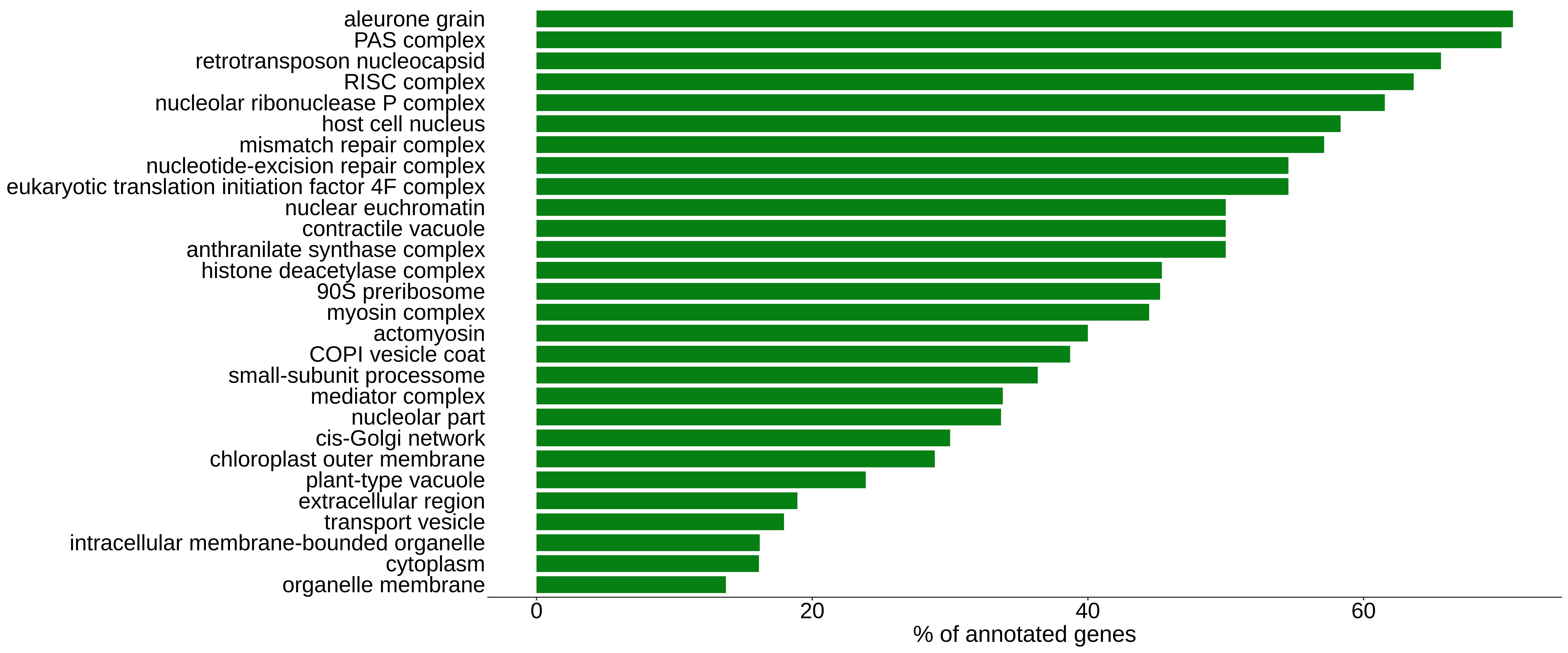

Supplement: Supplementary file 1 [file cells-09-00779-s001.zip › Supplementary materials/FigS12/B.tiff]

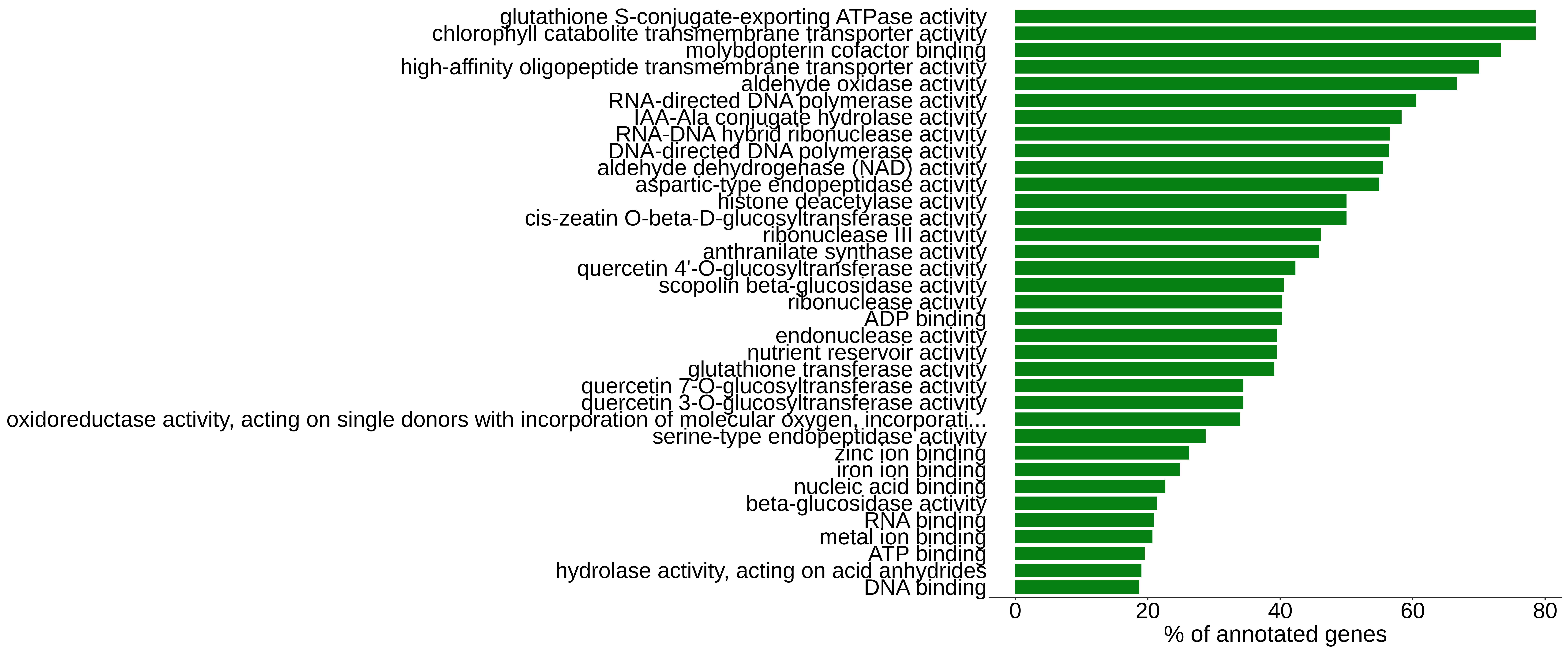

Supplement: Supplementary file 1 [file cells-09-00779-s001.zip › Supplementary materials/FigS12/C.tiff]

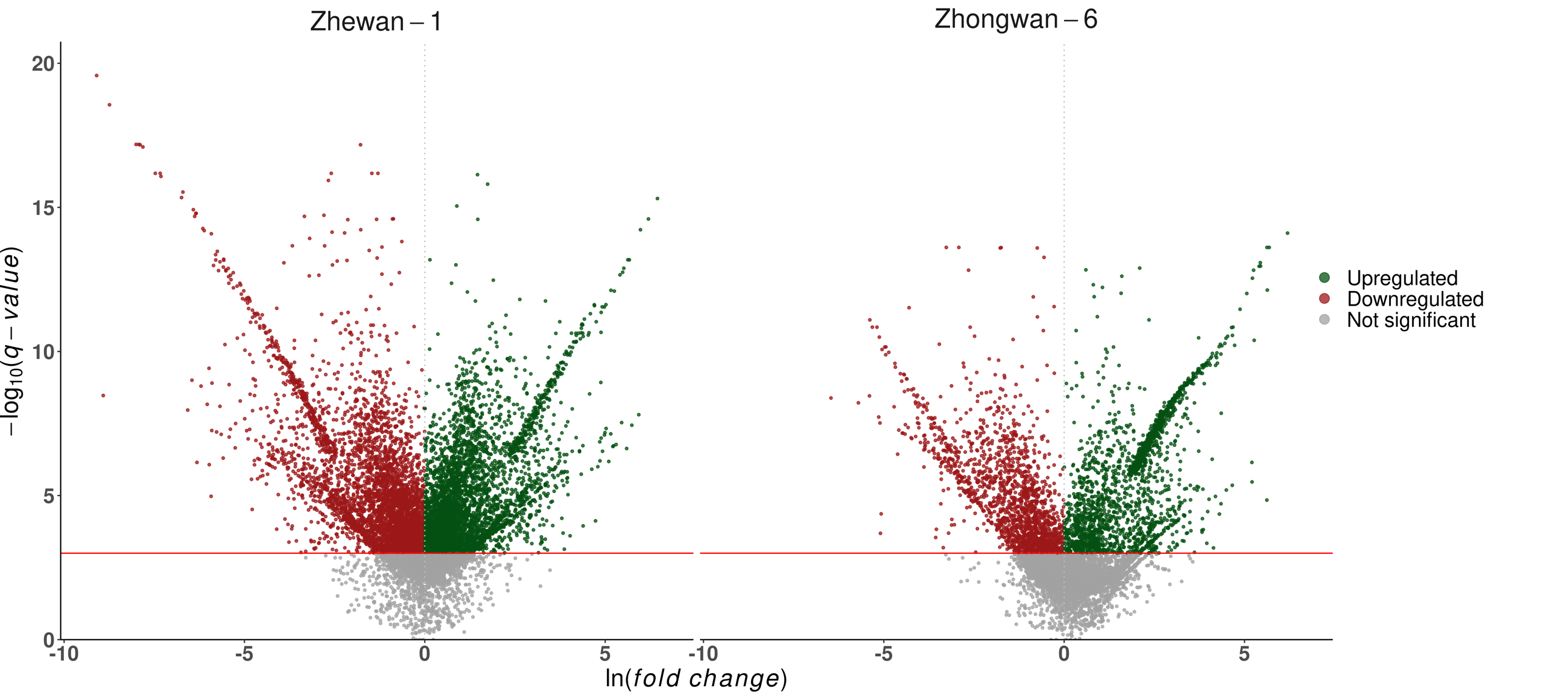

Supplement: Supplementary file 1 [file cells-09-00779-s001.zip › Supplementary materials/FigS2/A.tif]

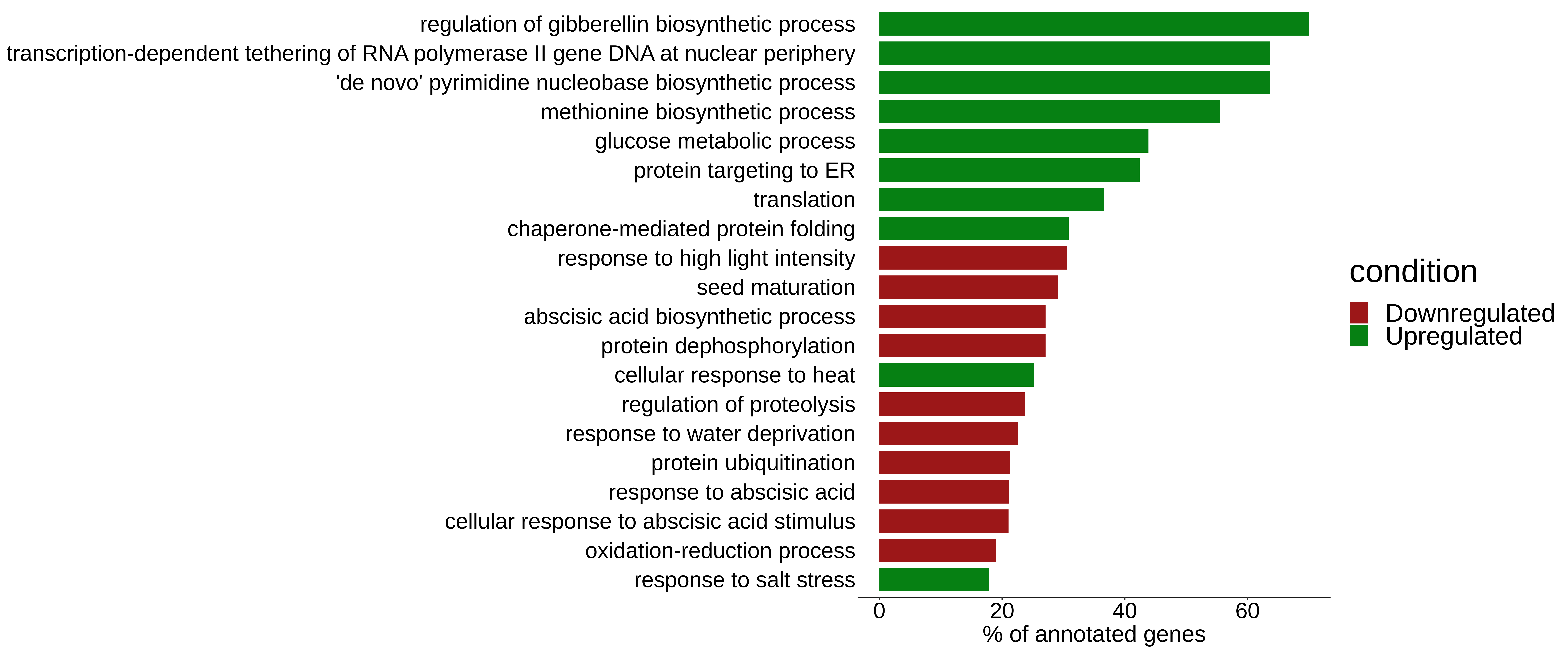

Supplement: Supplementary file 1 [file cells-09-00779-s001.zip › Supplementary materials/FigS2/B.tiff]

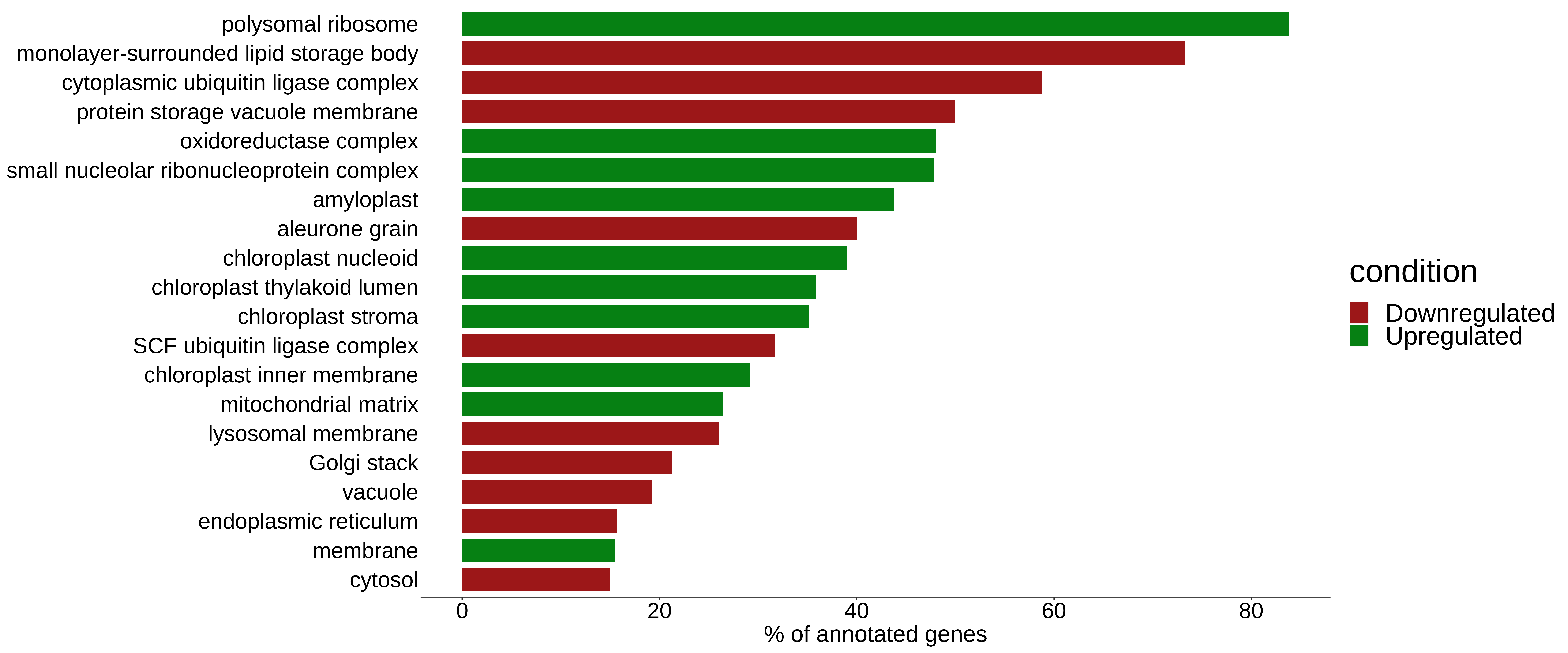

Supplement: Supplementary file 1 [file cells-09-00779-s001.zip › Supplementary materials/FigS2/C.tiff]

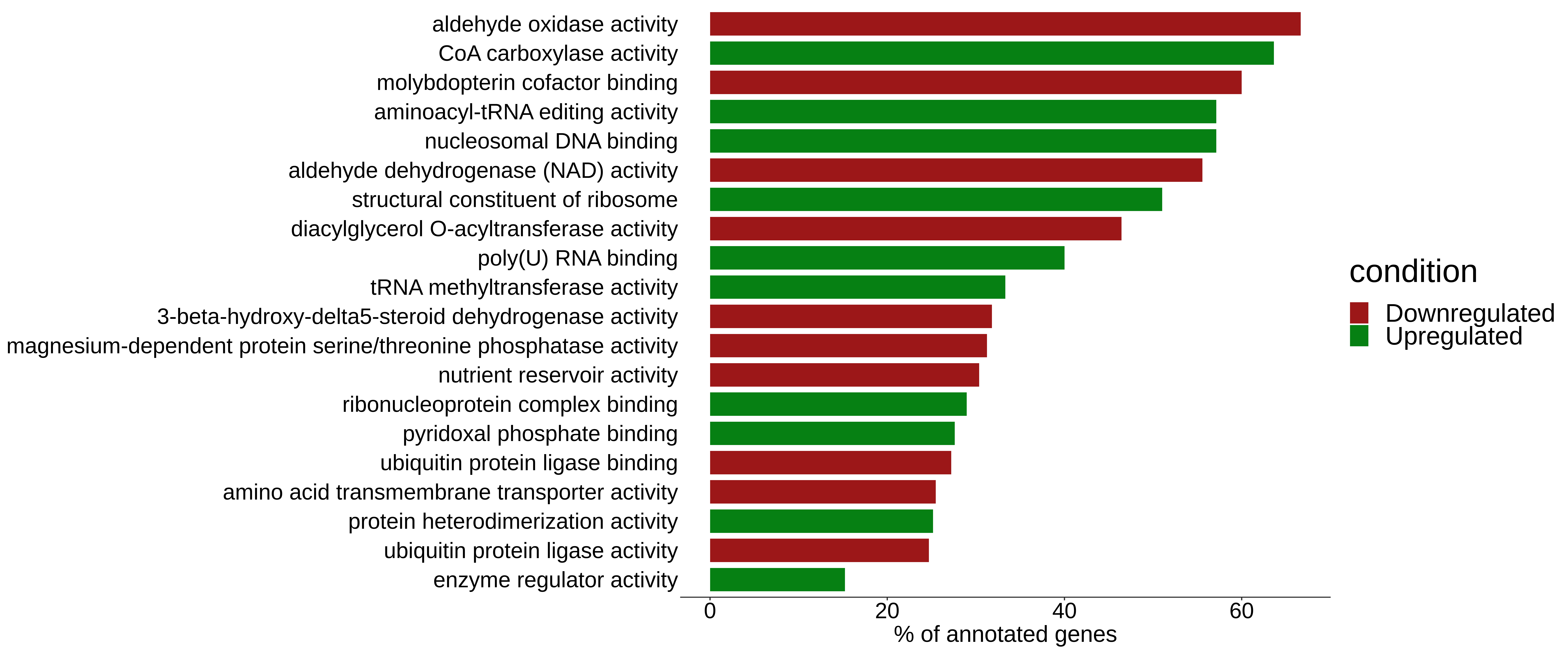

Supplement: Supplementary file 1 [file cells-09-00779-s001.zip › Supplementary materials/FigS2/D.tiff]

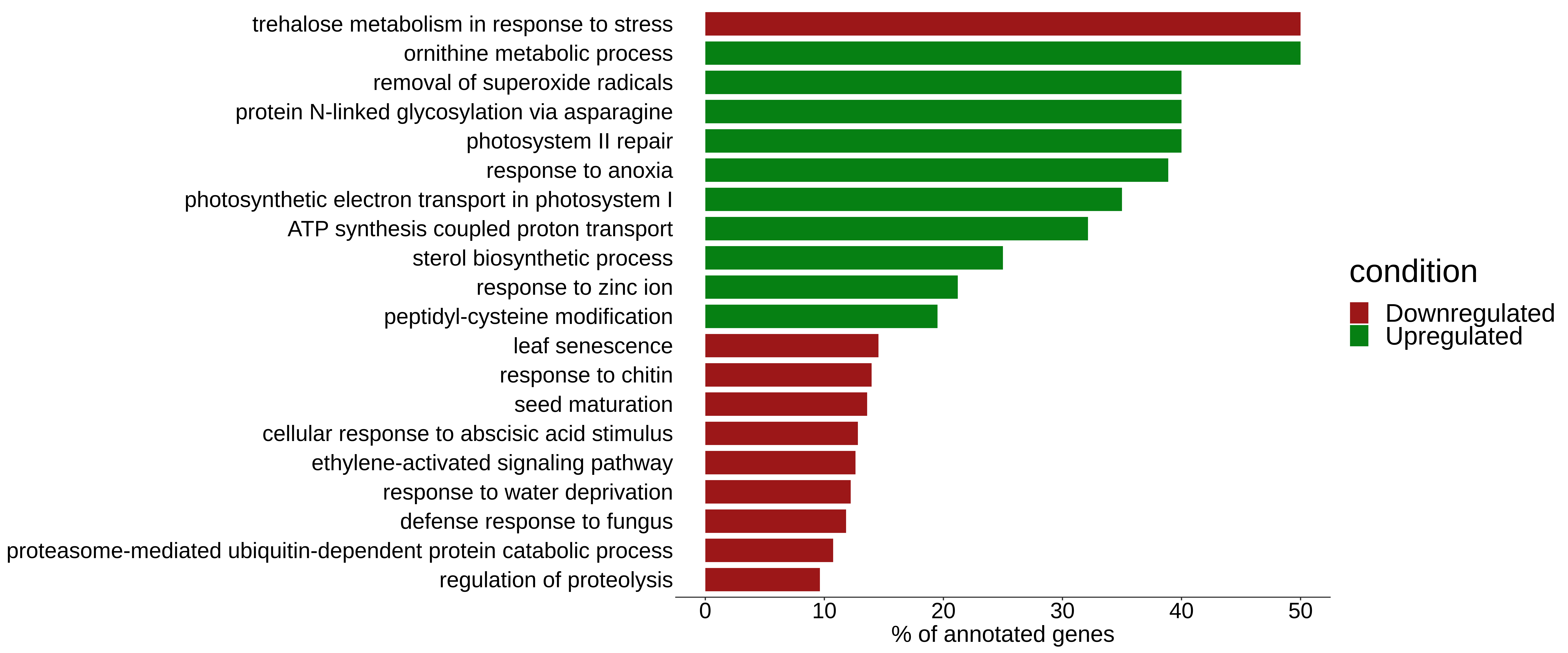

Supplement: Supplementary file 1 [file cells-09-00779-s001.zip › Supplementary materials/FigS2/E.tiff]

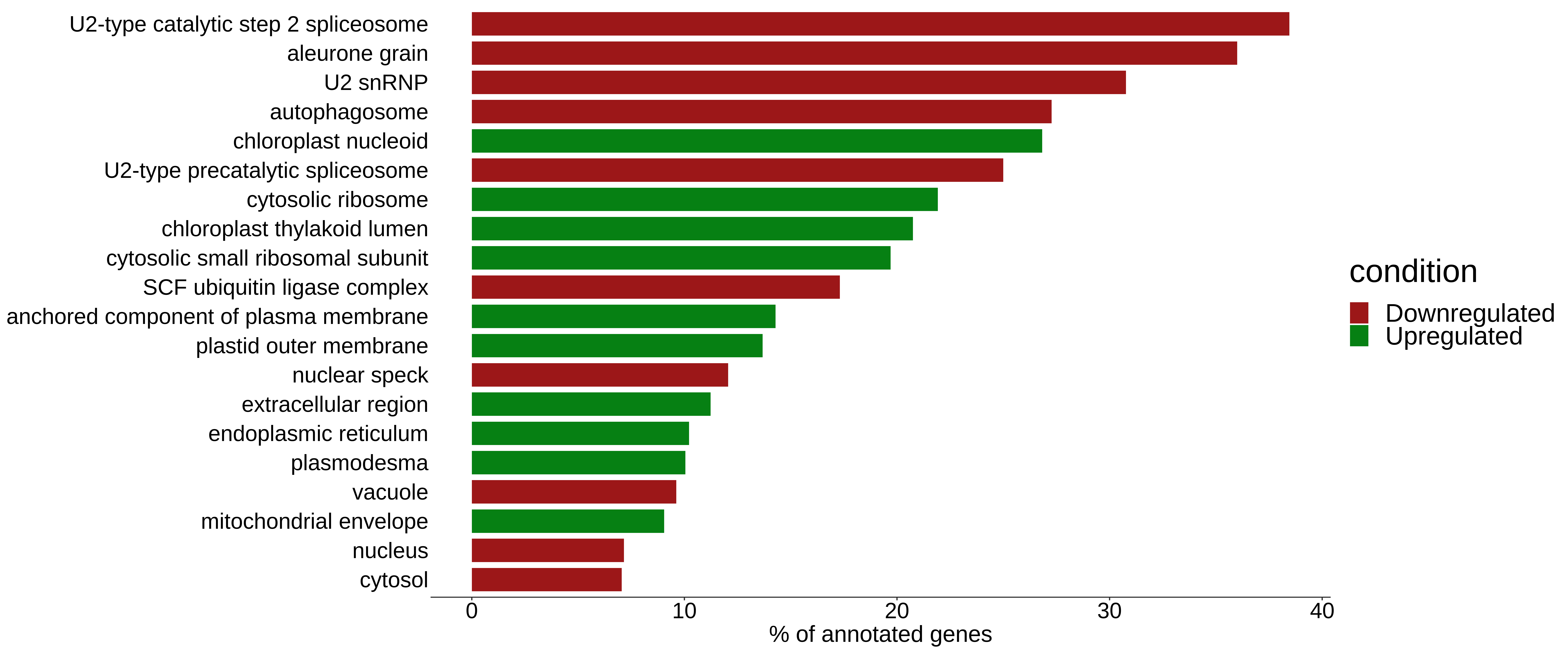

Supplement: Supplementary file 1 [file cells-09-00779-s001.zip › Supplementary materials/FigS2/F.tiff]

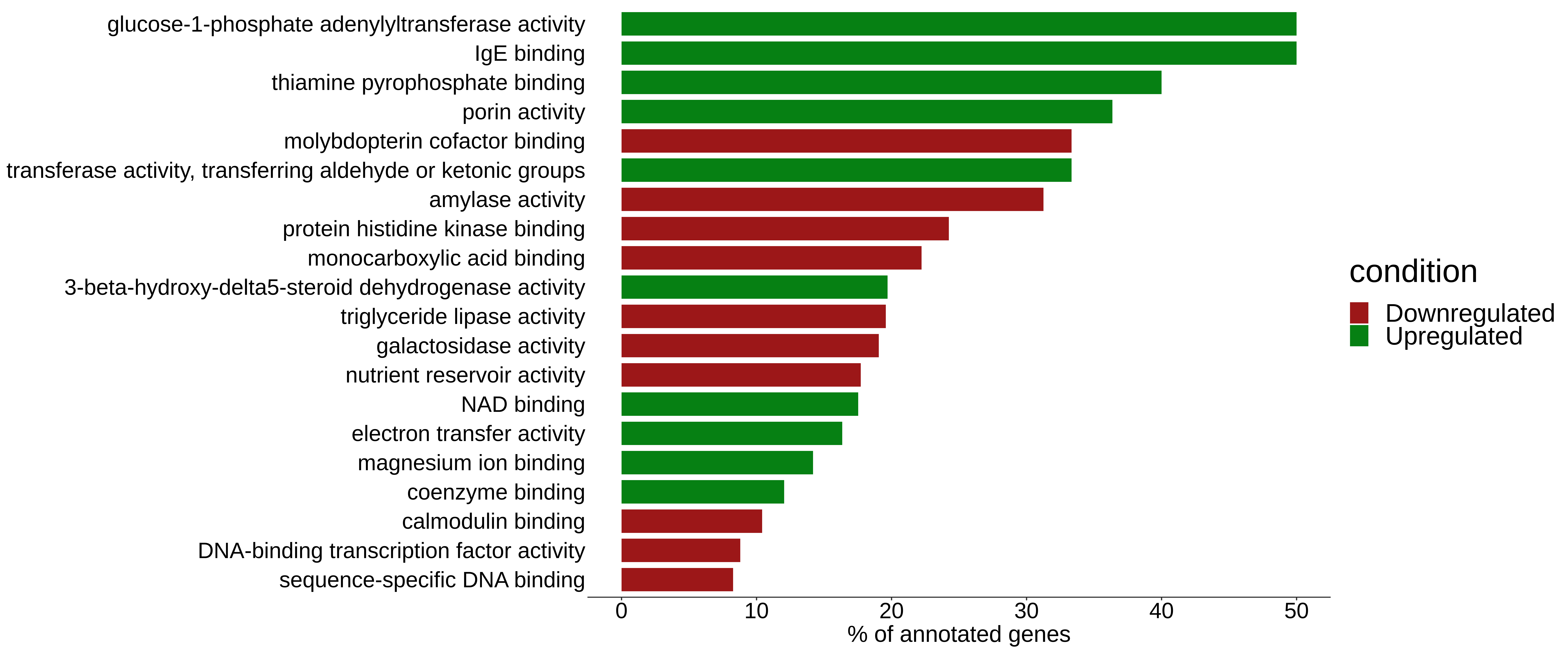

Supplement: Supplementary file 1 [file cells-09-00779-s001.zip › Supplementary materials/FigS2/G.tiff]

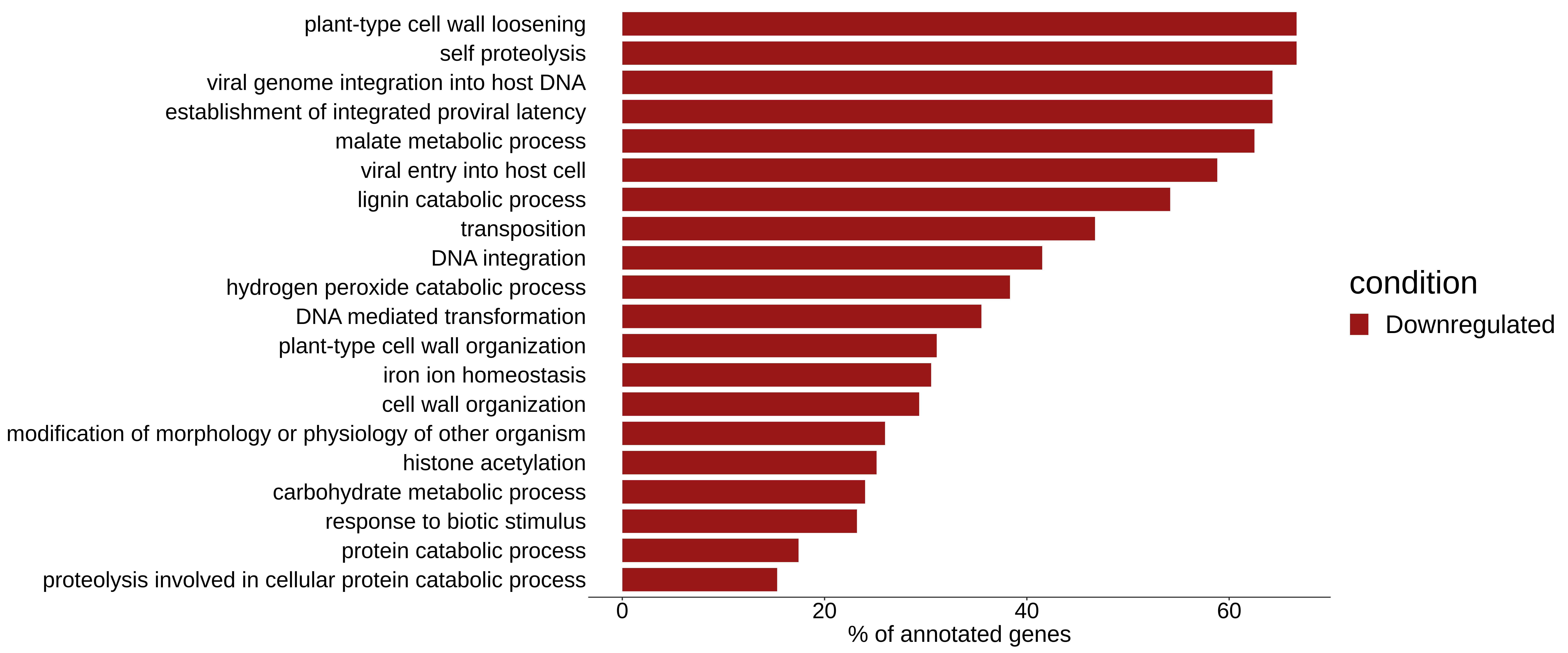

Supplement: Supplementary file 1 [file cells-09-00779-s001.zip › Supplementary materials/FigS4/A.tiff]

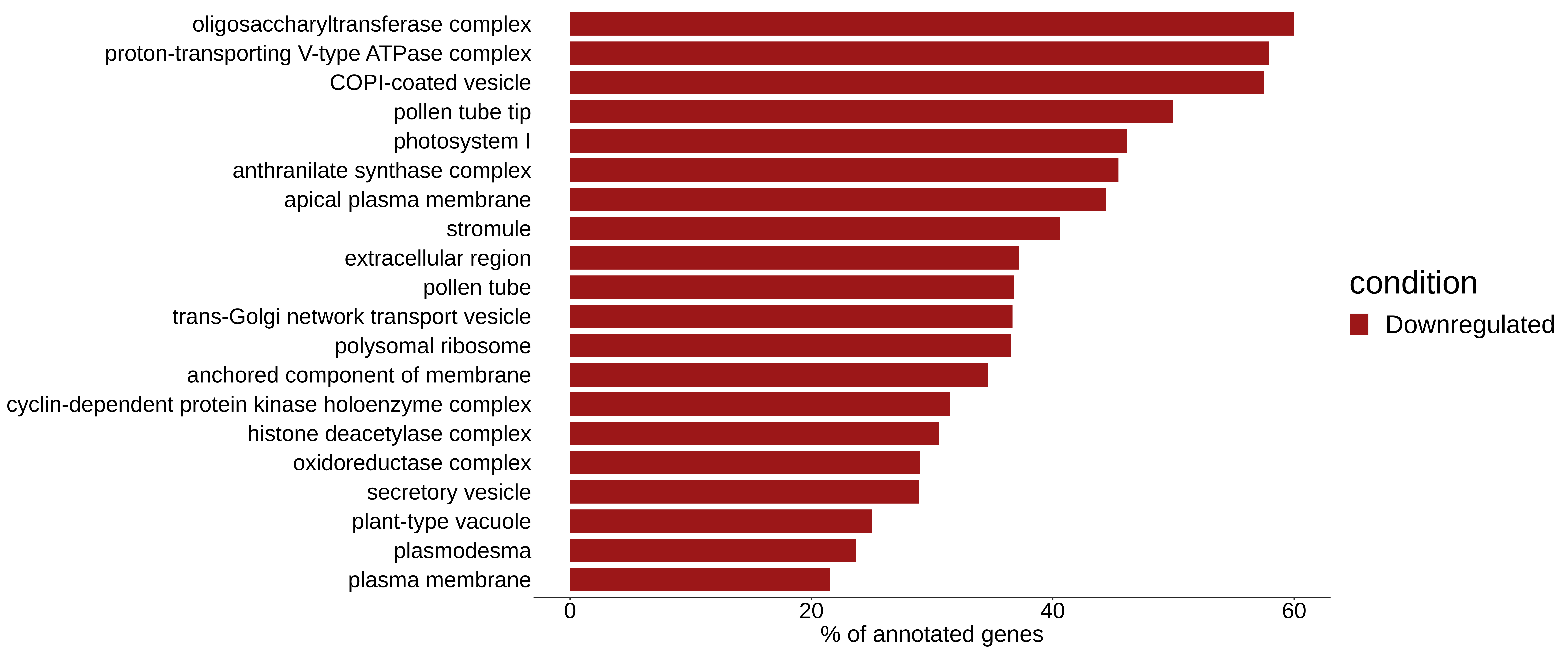

Supplement: Supplementary file 1 [file cells-09-00779-s001.zip › Supplementary materials/FigS4/B.tiff]

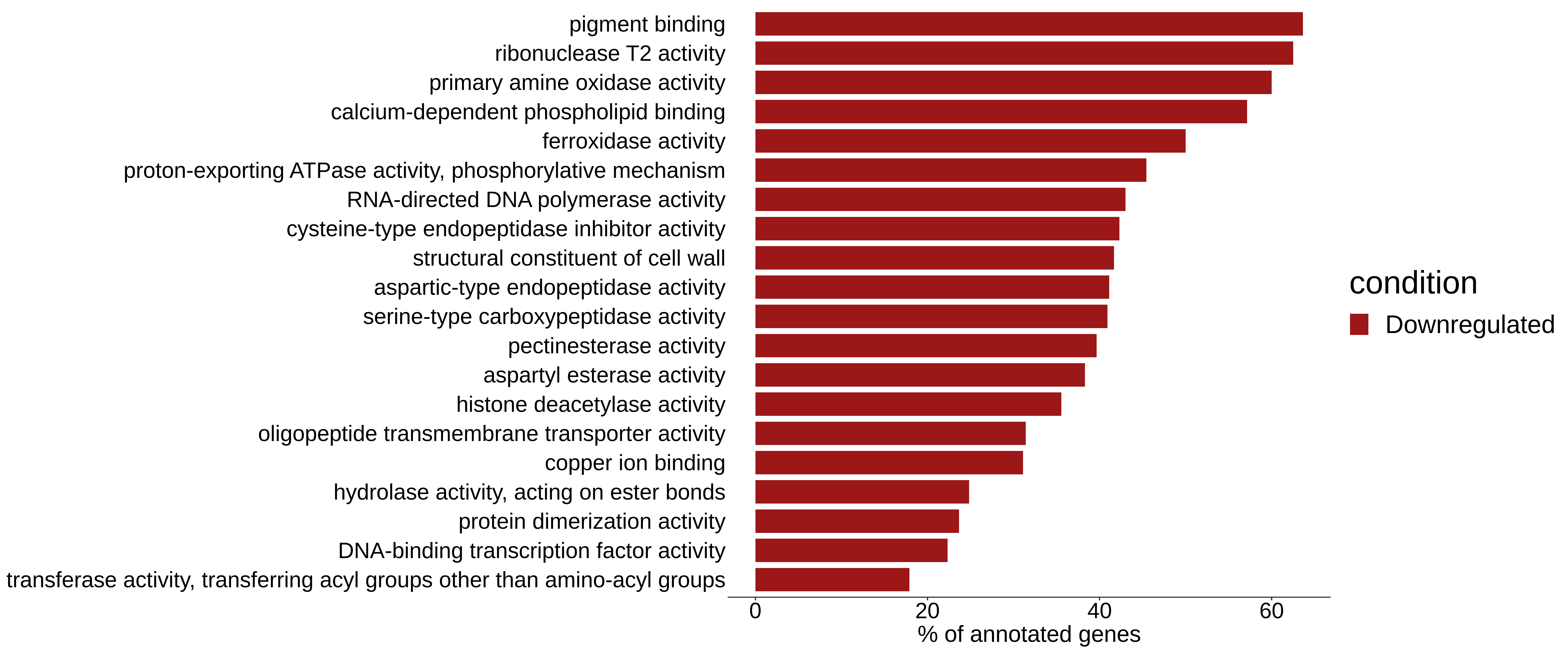

Supplement: Supplementary file 1 [file cells-09-00779-s001.zip › Supplementary materials/FigS4/C.tiff]

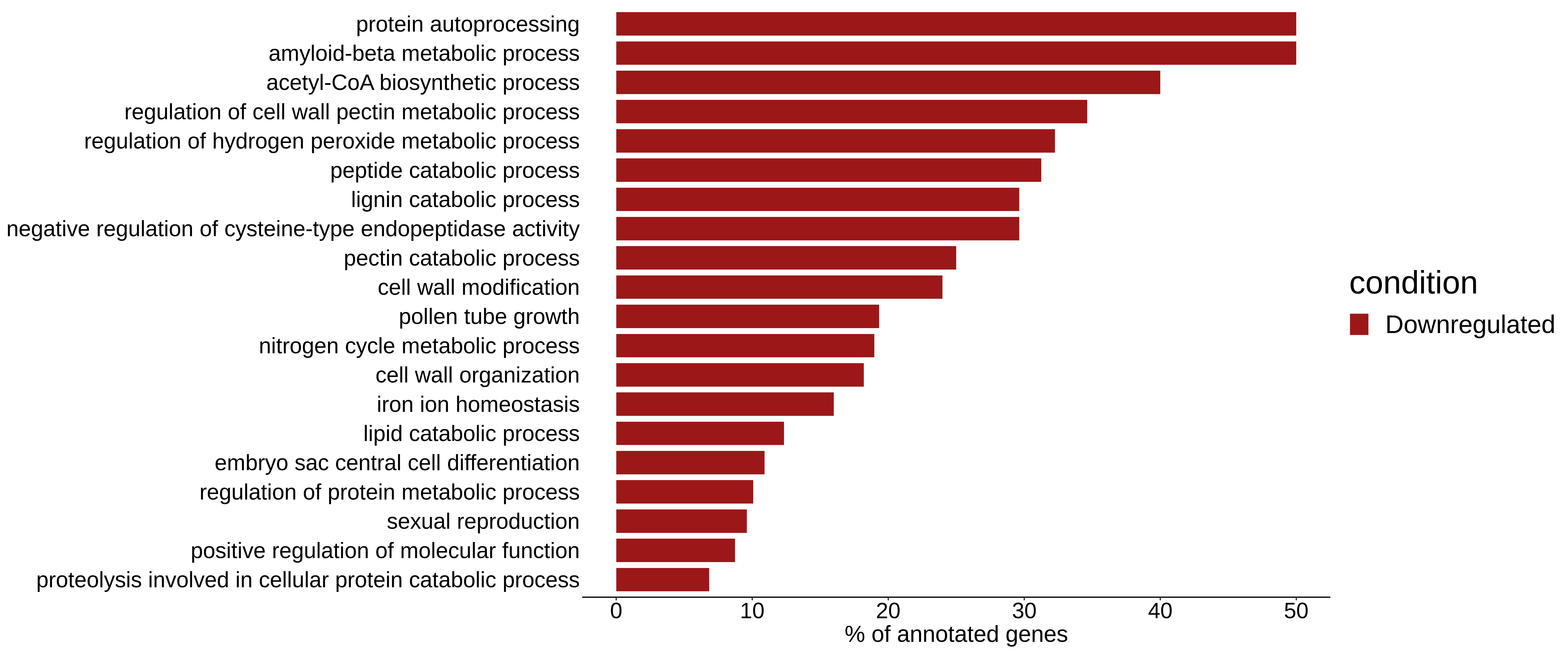

Supplement: Supplementary file 1 [file cells-09-00779-s001.zip › Supplementary materials/FigS5/A.tiff]

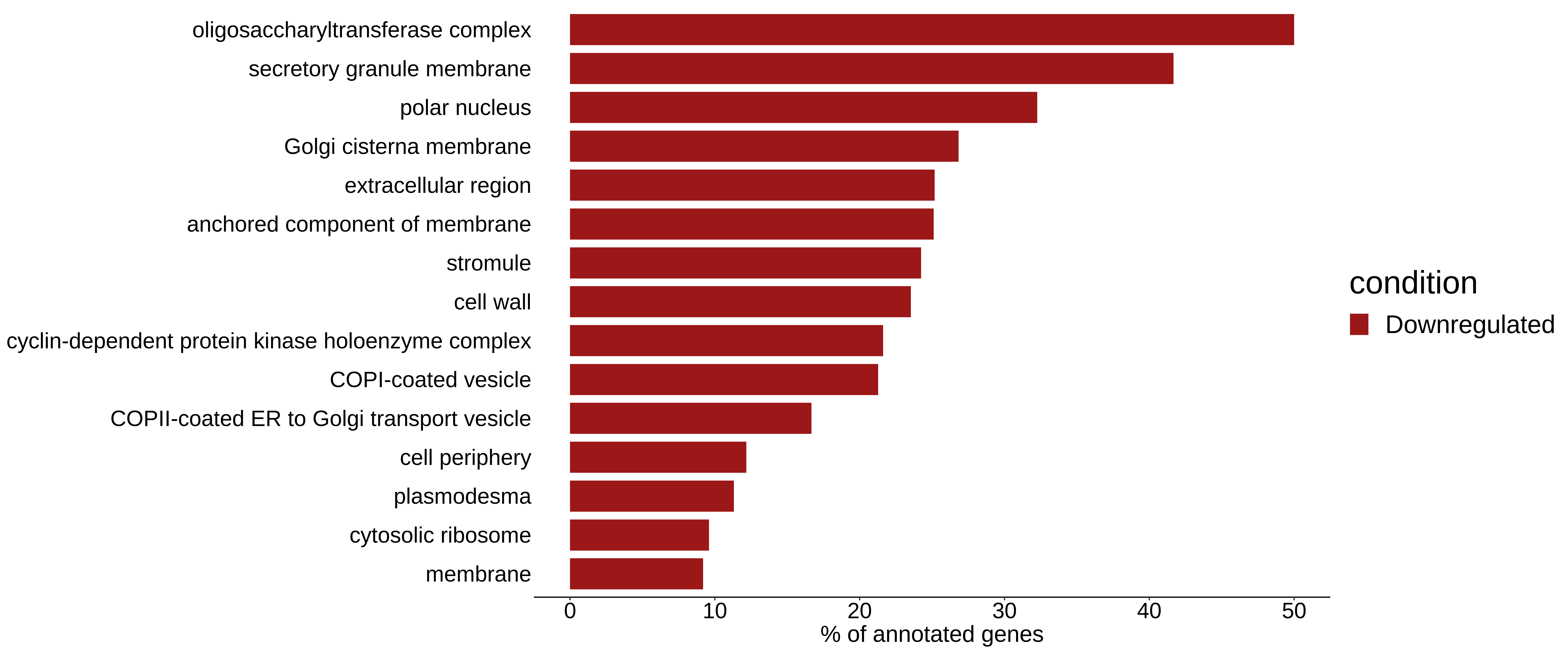

Supplement: Supplementary file 1 [file cells-09-00779-s001.zip › Supplementary materials/FigS5/B.tiff]

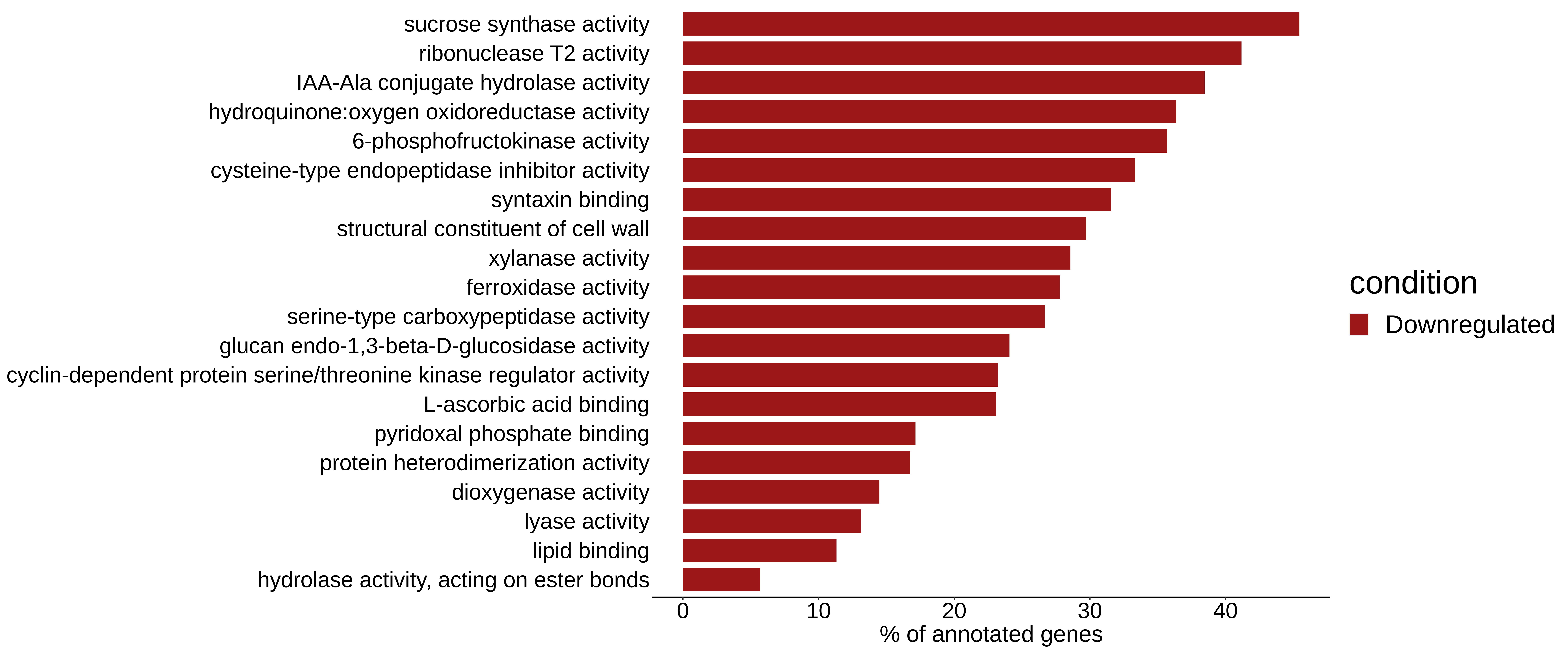

Supplement: Supplementary file 1 [file cells-09-00779-s001.zip › Supplementary materials/FigS5/C.tiff]

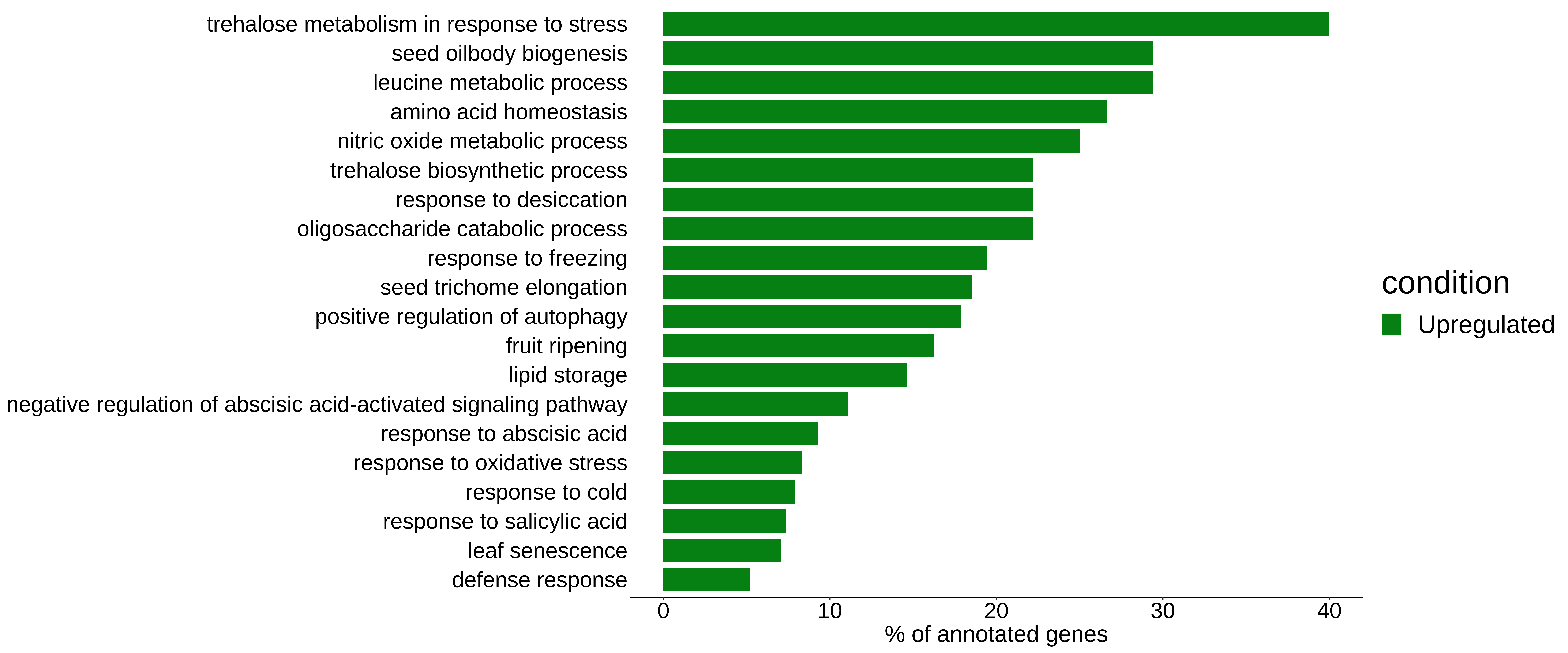

Supplement: Supplementary file 1 [file cells-09-00779-s001.zip › Supplementary materials/FigS6/A.tiff]

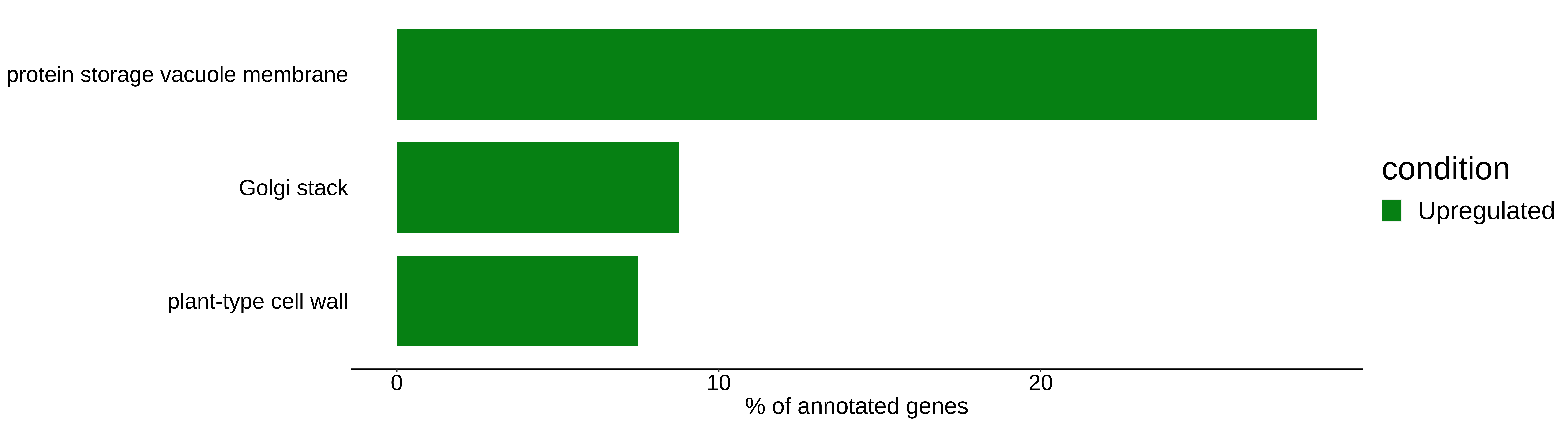

Supplement: Supplementary file 1 [file cells-09-00779-s001.zip › Supplementary materials/FigS6/B.tiff]

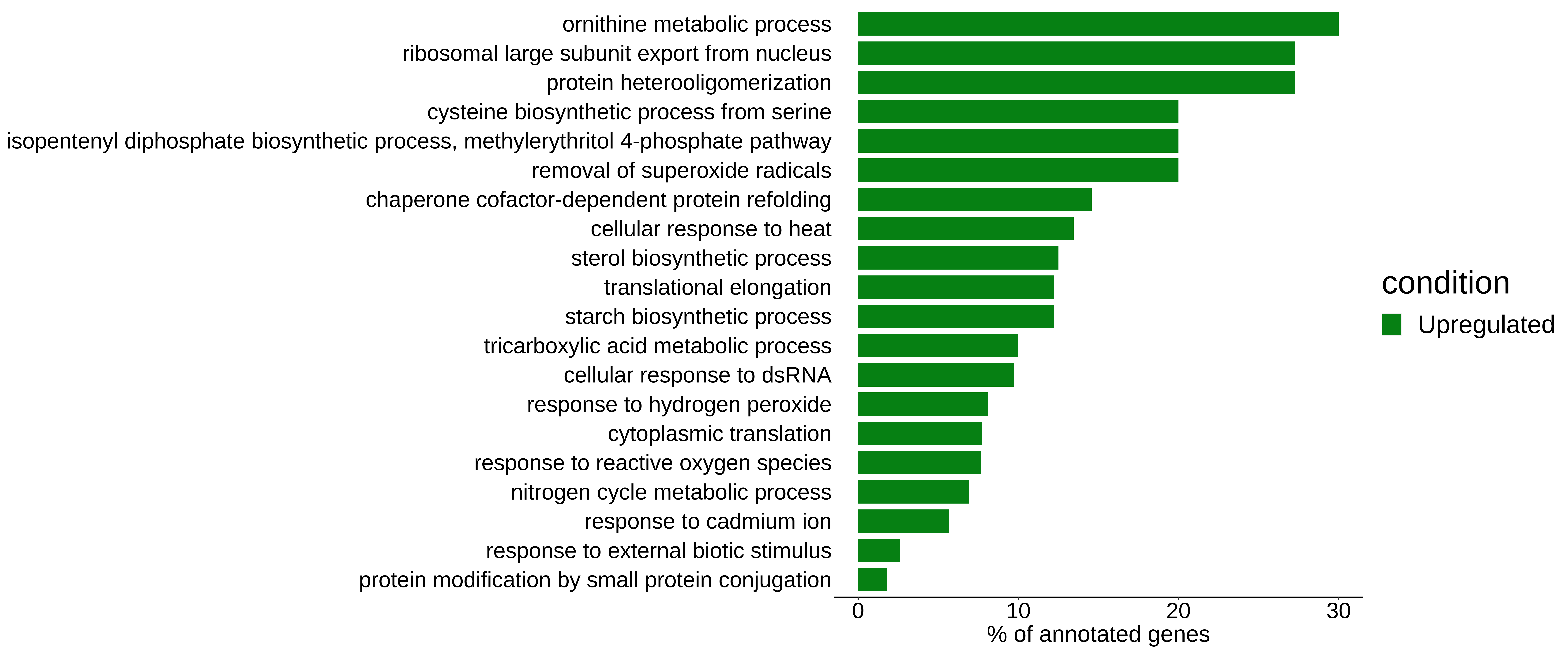

Supplement: Supplementary file 1 [file cells-09-00779-s001.zip › Supplementary materials/FigS7/A.tiff]

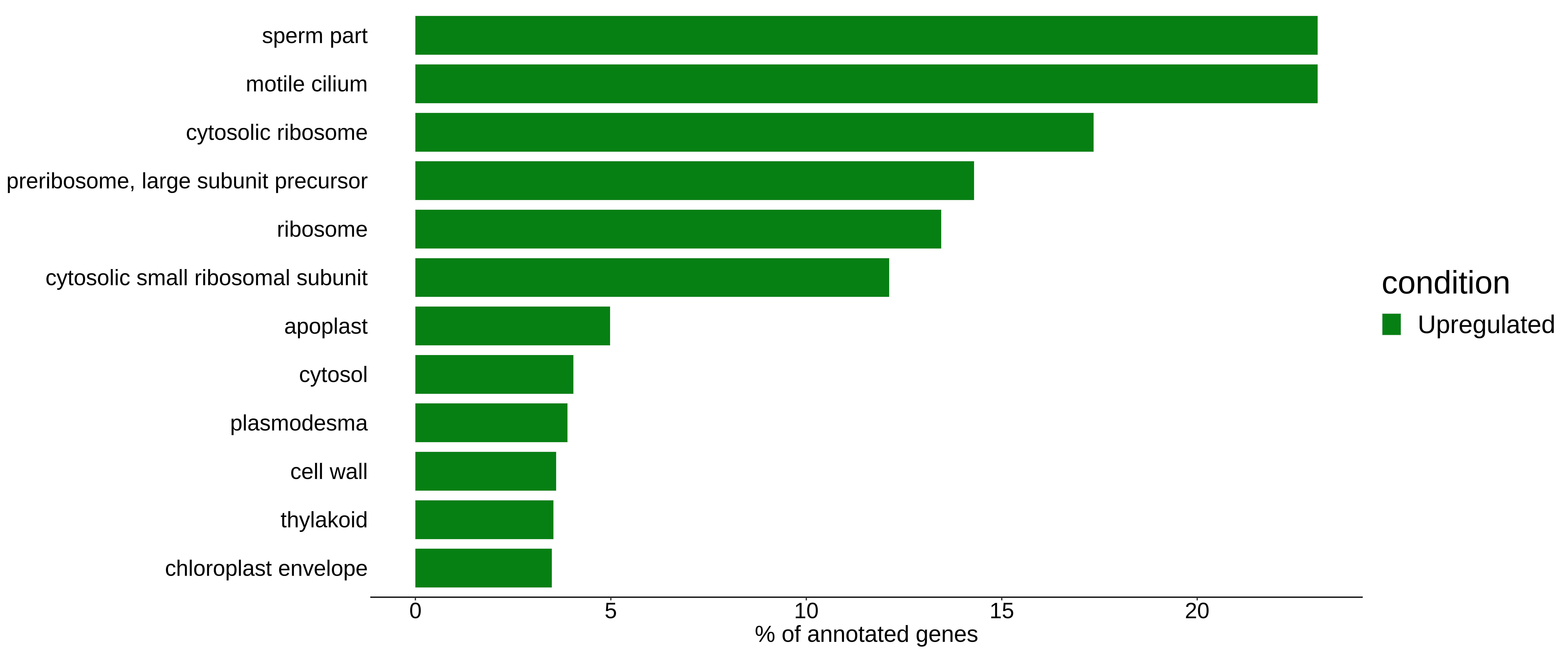

Supplement: Supplementary file 1 [file cells-09-00779-s001.zip › Supplementary materials/FigS7/B.tiff]

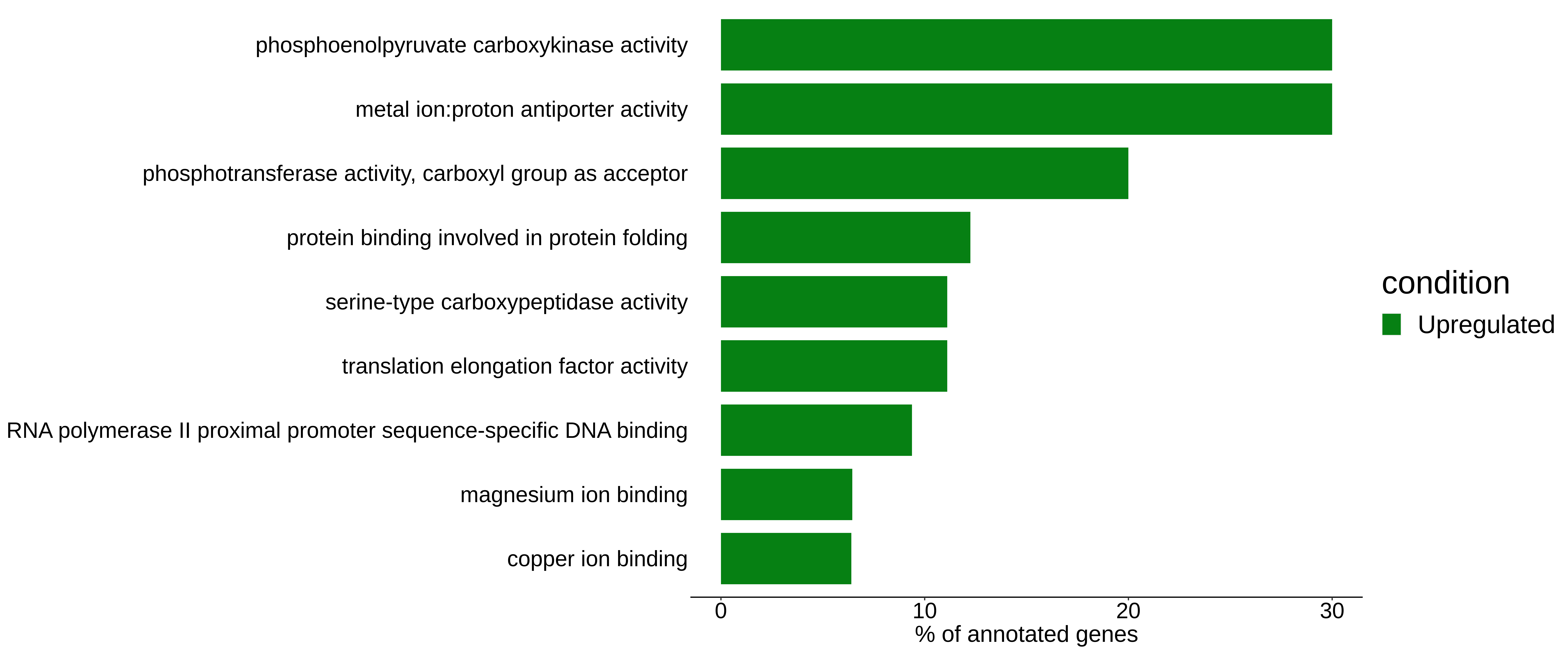

Supplement: Supplementary file 1 [file cells-09-00779-s001.zip › Supplementary materials/FigS7/C.tiff]

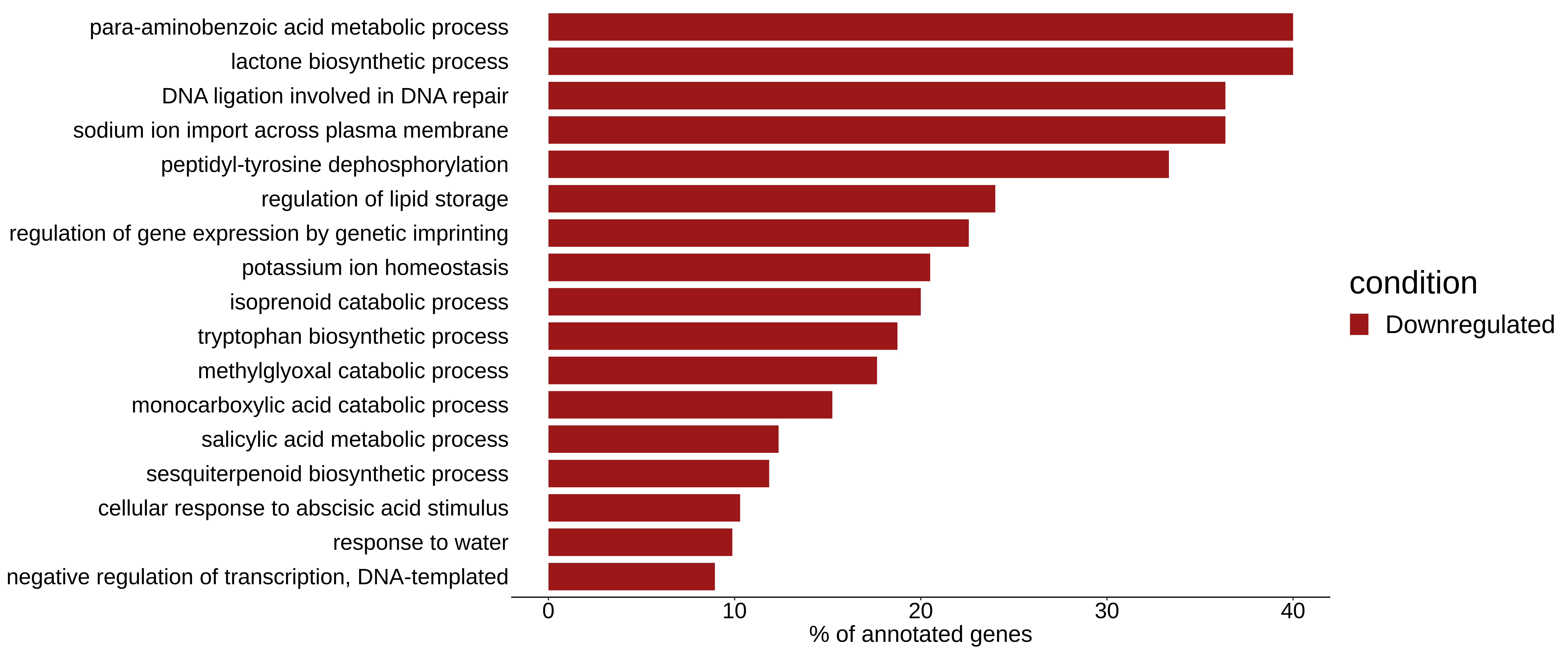

Supplement: Supplementary file 1 [file cells-09-00779-s001.zip › Supplementary materials/FigS8/A.tiff]

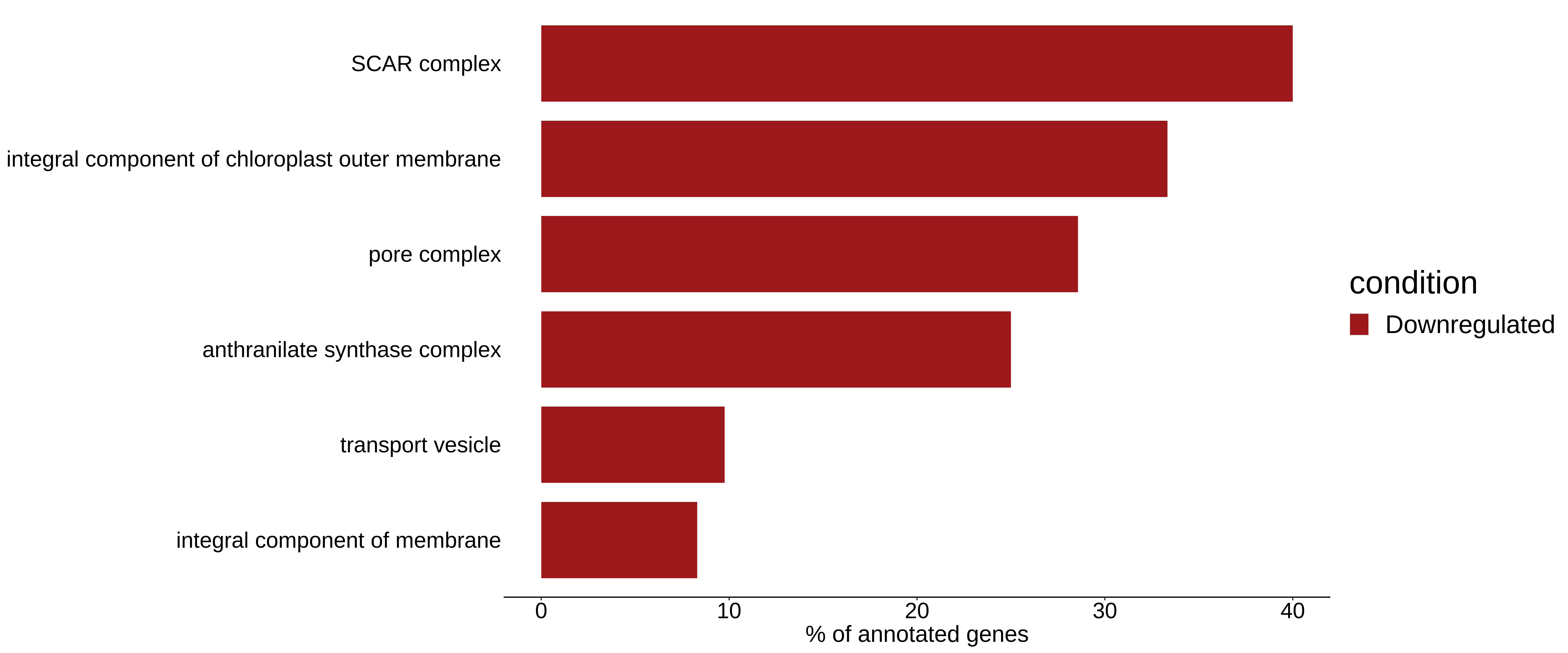

Supplement: Supplementary file 1 [file cells-09-00779-s001.zip › Supplementary materials/FigS8/B.tiff]

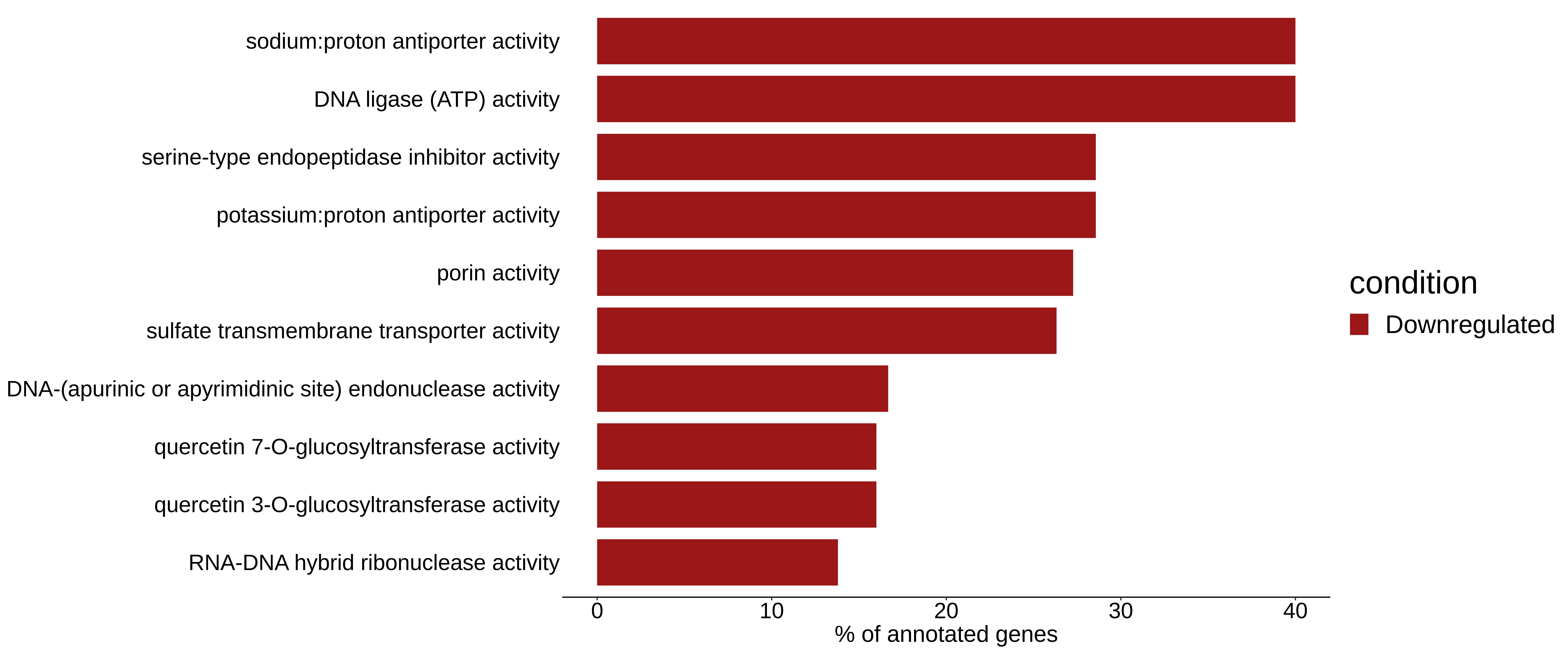

Supplement: Supplementary file 1 [file cells-09-00779-s001.zip › Supplementary materials/FigS8/C.tiff]

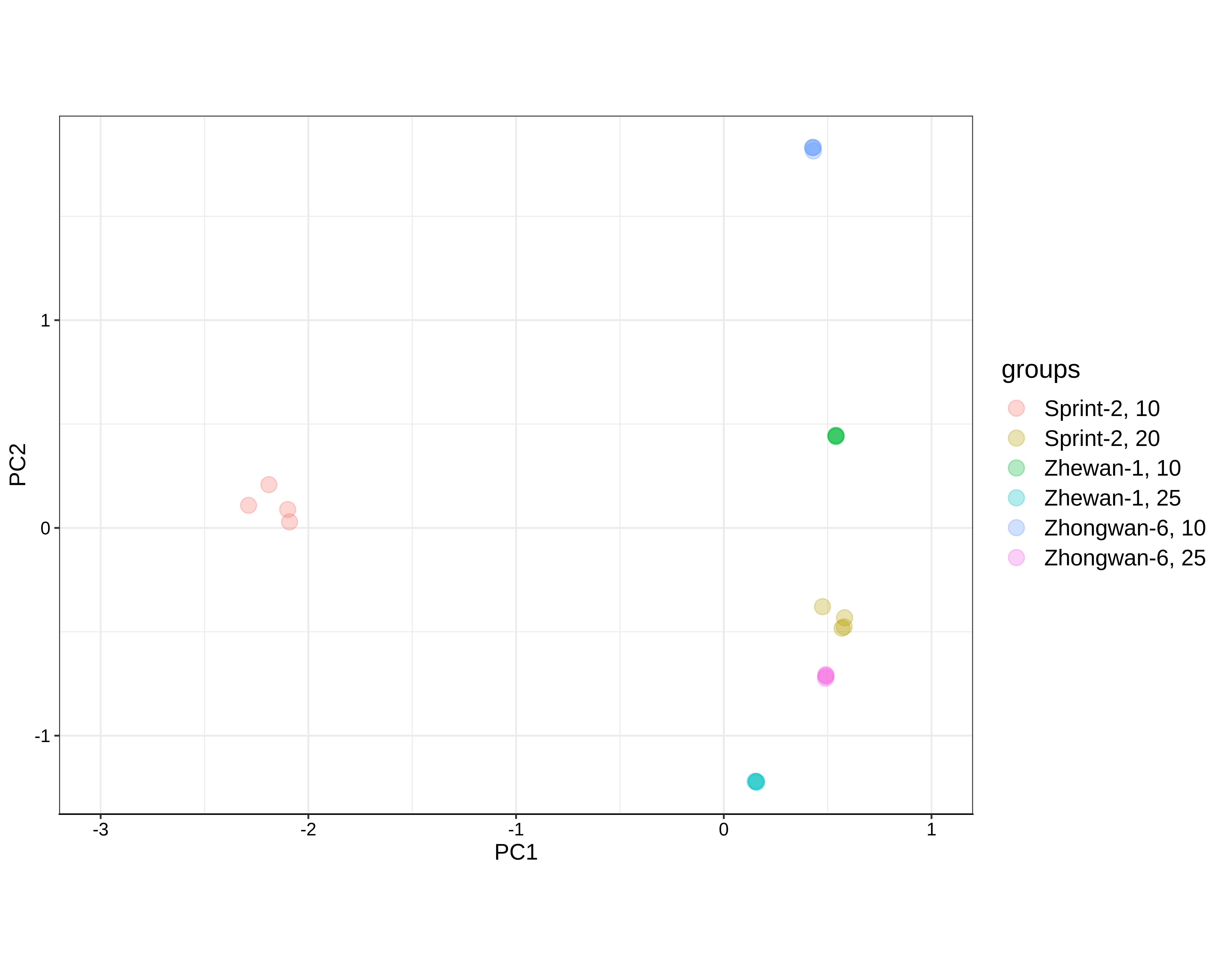

Supplement: Supplementary file 1 [file cells-09-00779-s001.zip › Supplementary materials/FigS9/1.tiff]

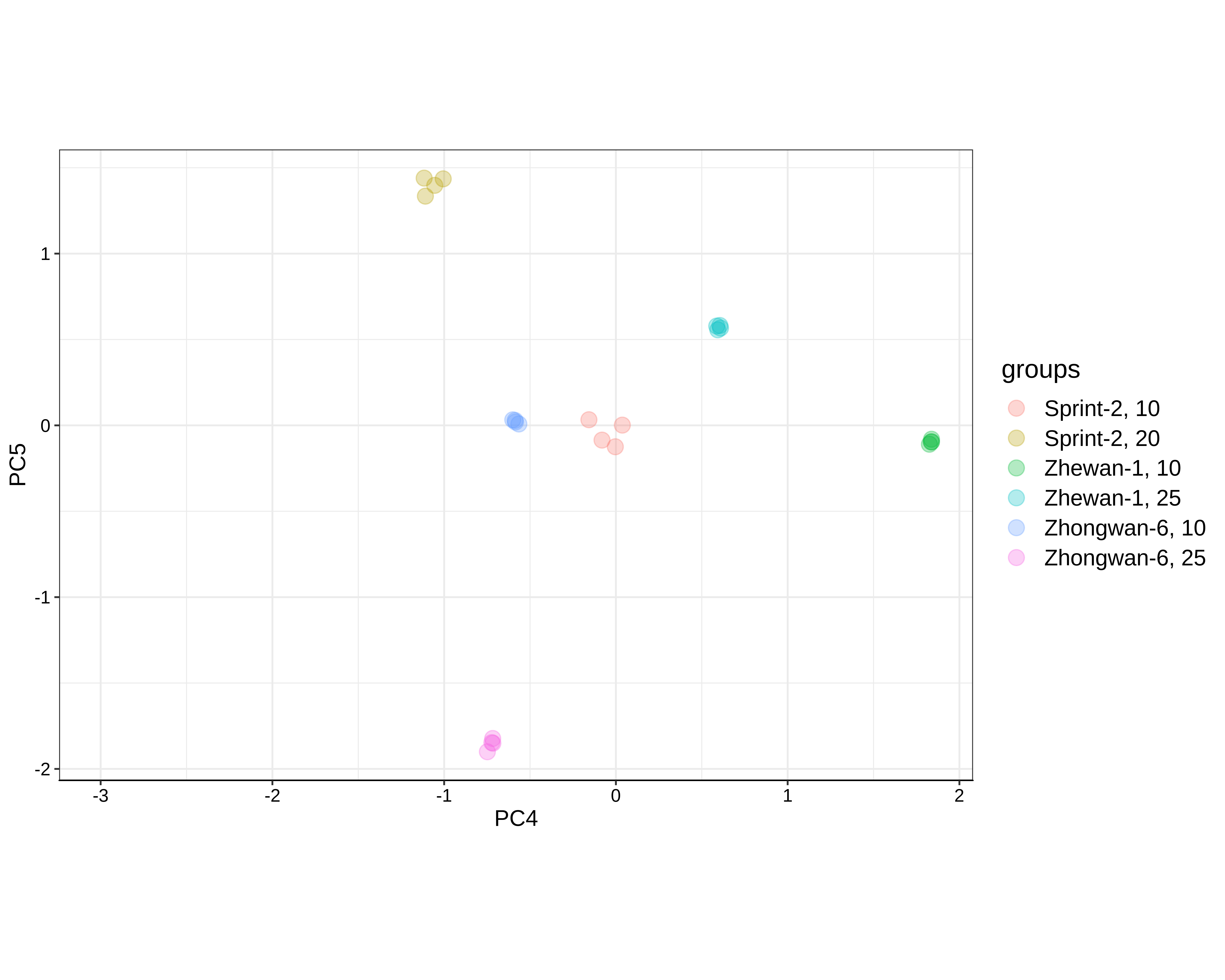

Supplement: Supplementary file 1 [file cells-09-00779-s001.zip › Supplementary materials/FigS9/10.tiff]

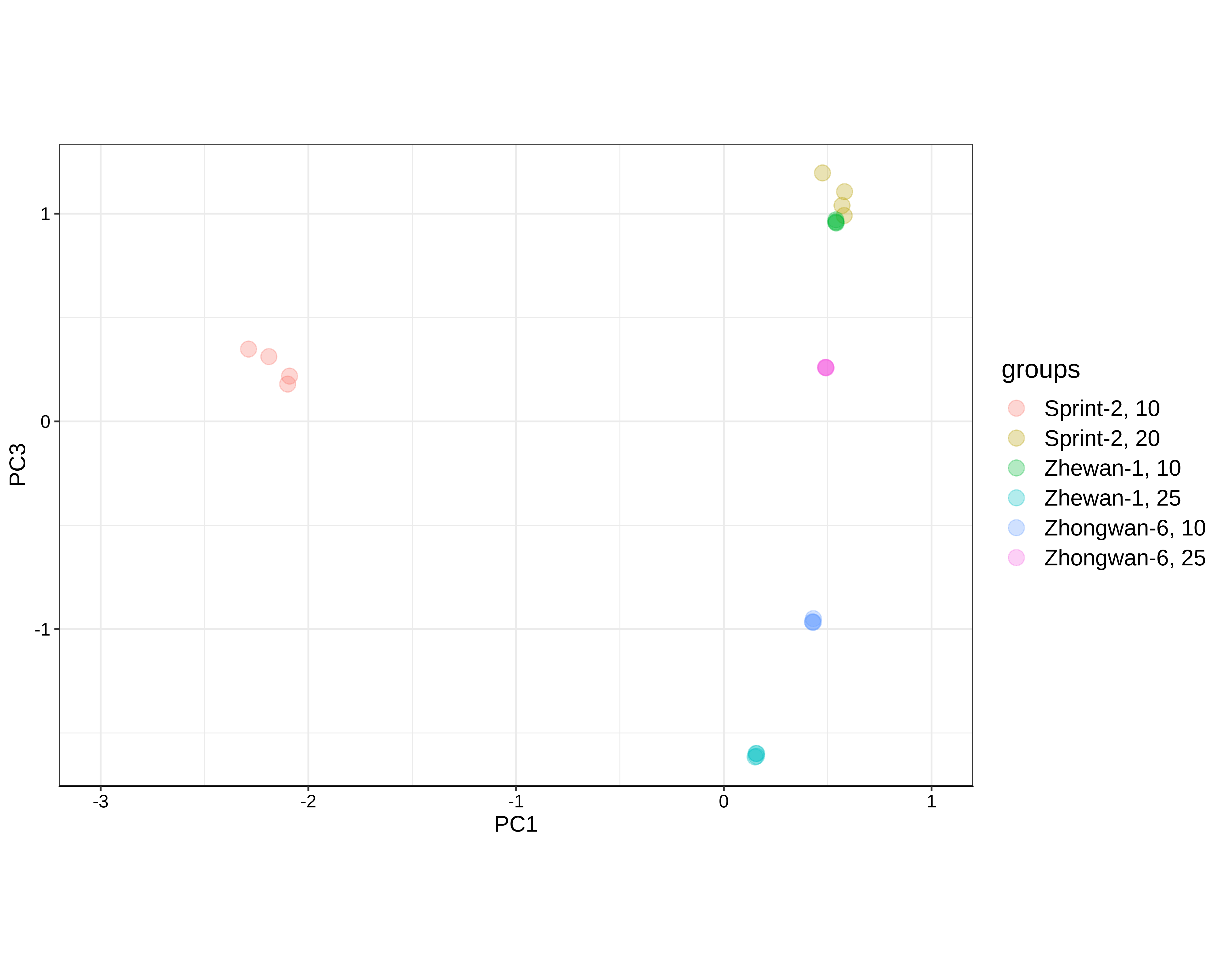

Supplement: Supplementary file 1 [file cells-09-00779-s001.zip › Supplementary materials/FigS9/2.tiff]

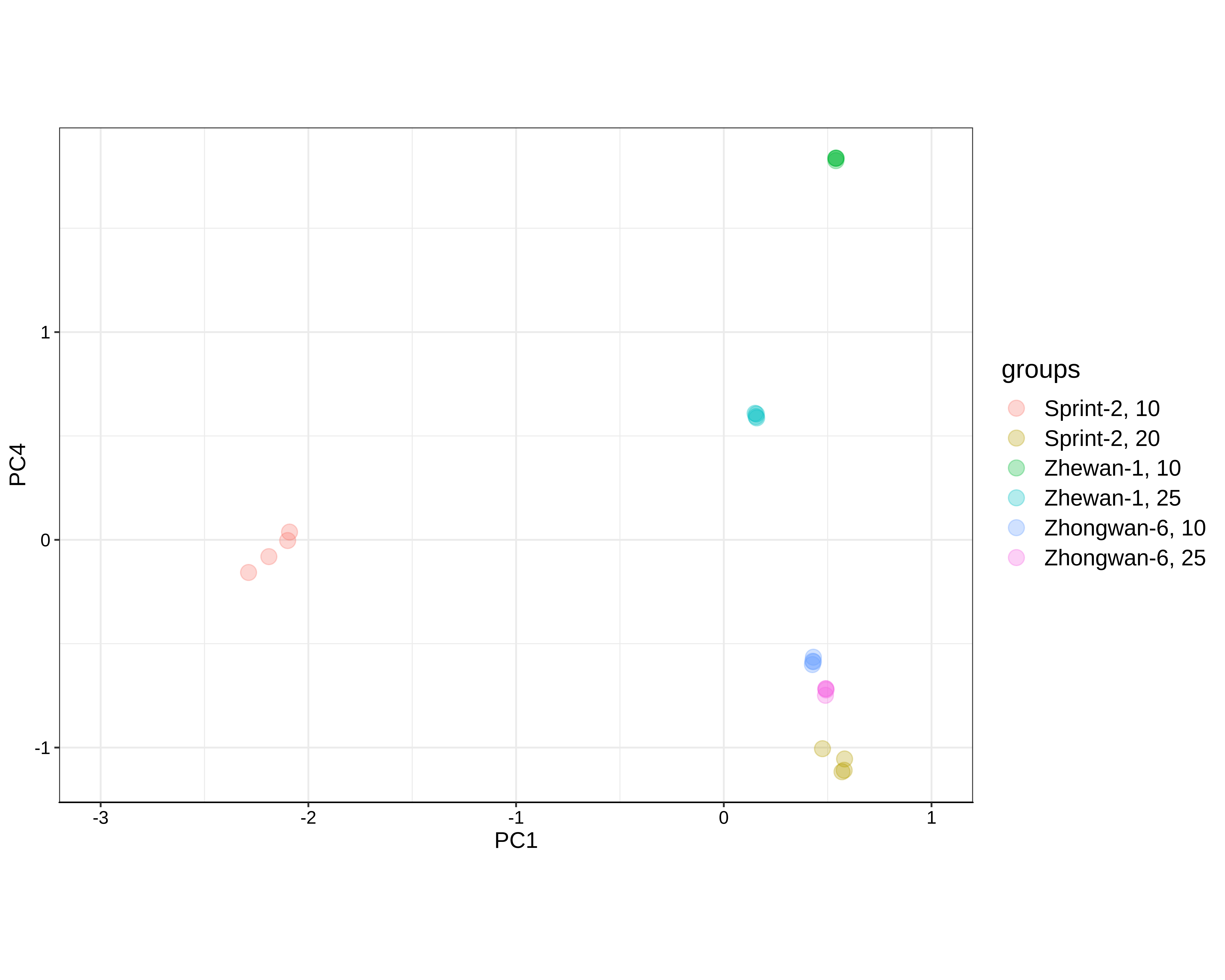

Supplement: Supplementary file 1 [file cells-09-00779-s001.zip › Supplementary materials/FigS9/3.tiff]

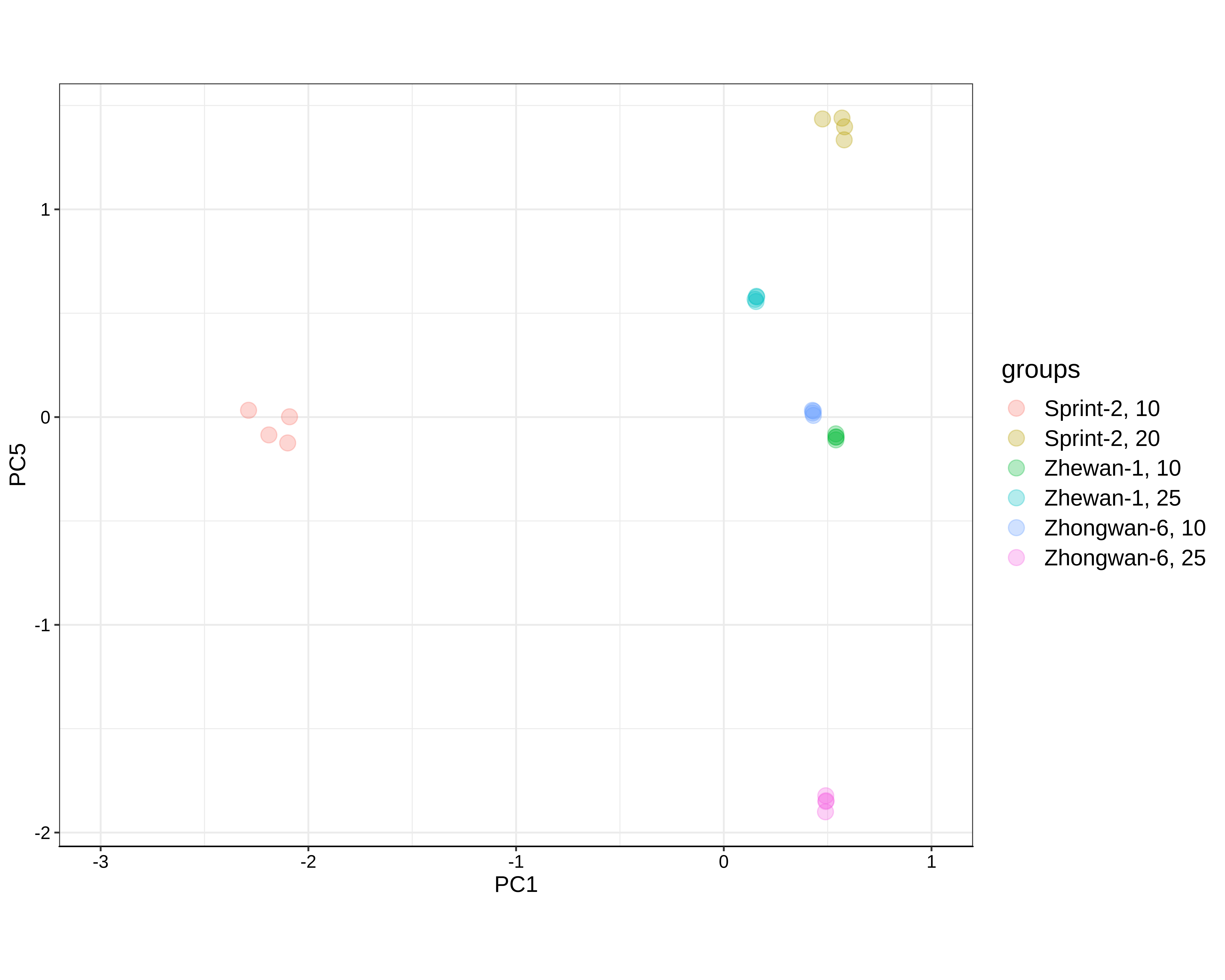

Supplement: Supplementary file 1 [file cells-09-00779-s001.zip › Supplementary materials/FigS9/4.tiff]

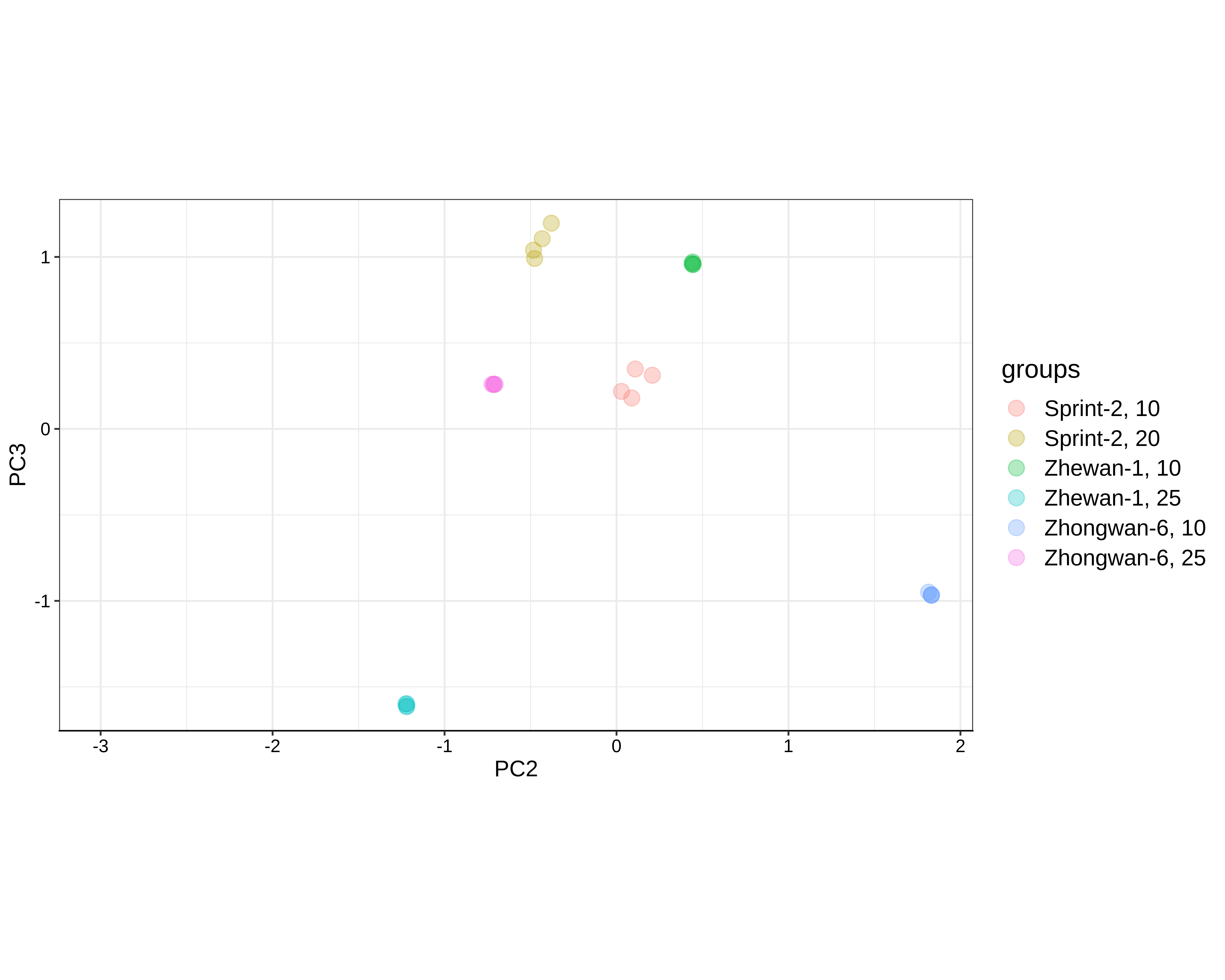

Supplement: Supplementary file 1 [file cells-09-00779-s001.zip › Supplementary materials/FigS9/5.tiff]

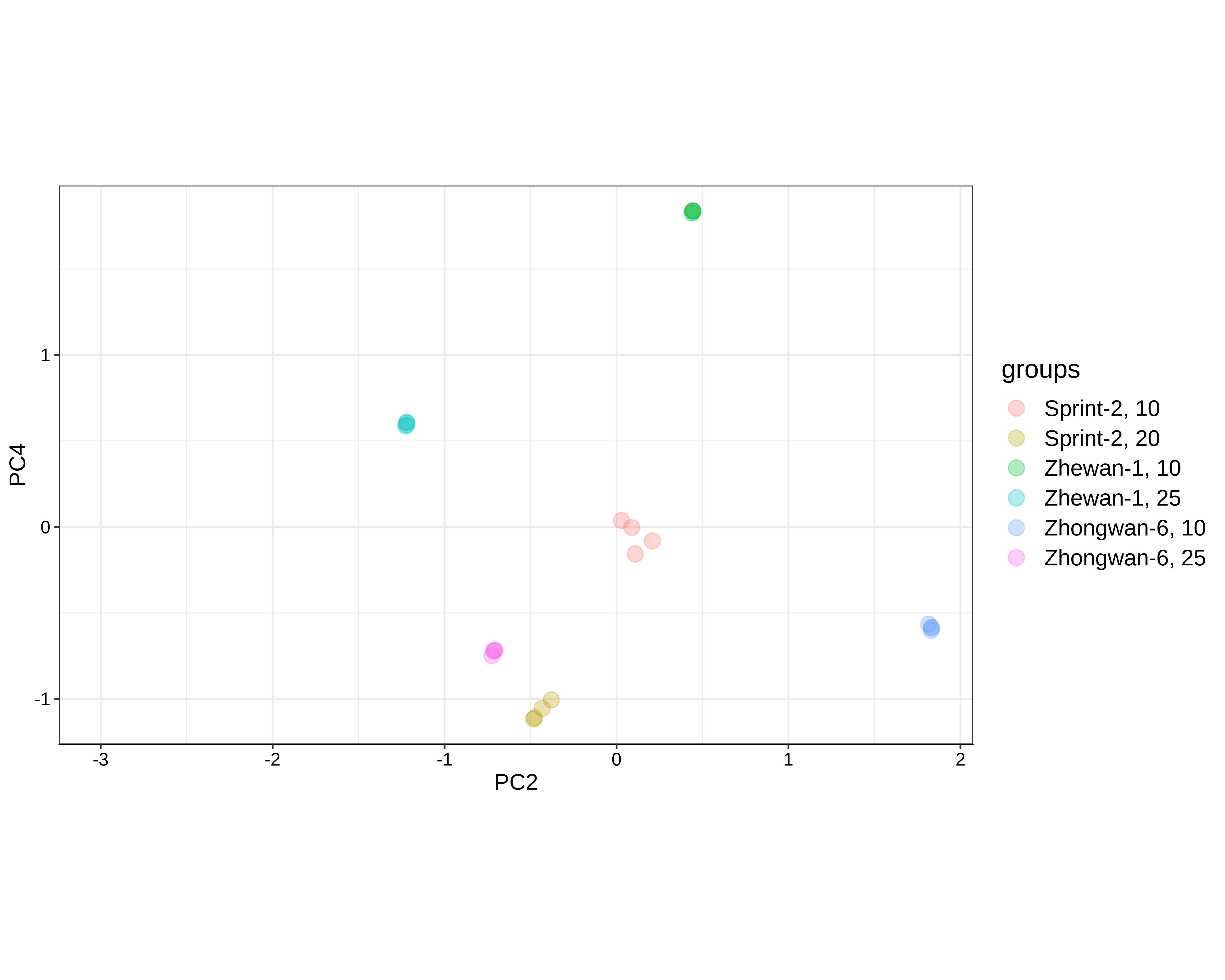

Supplement: Supplementary file 1 [file cells-09-00779-s001.zip › Supplementary materials/FigS9/6.tiff]

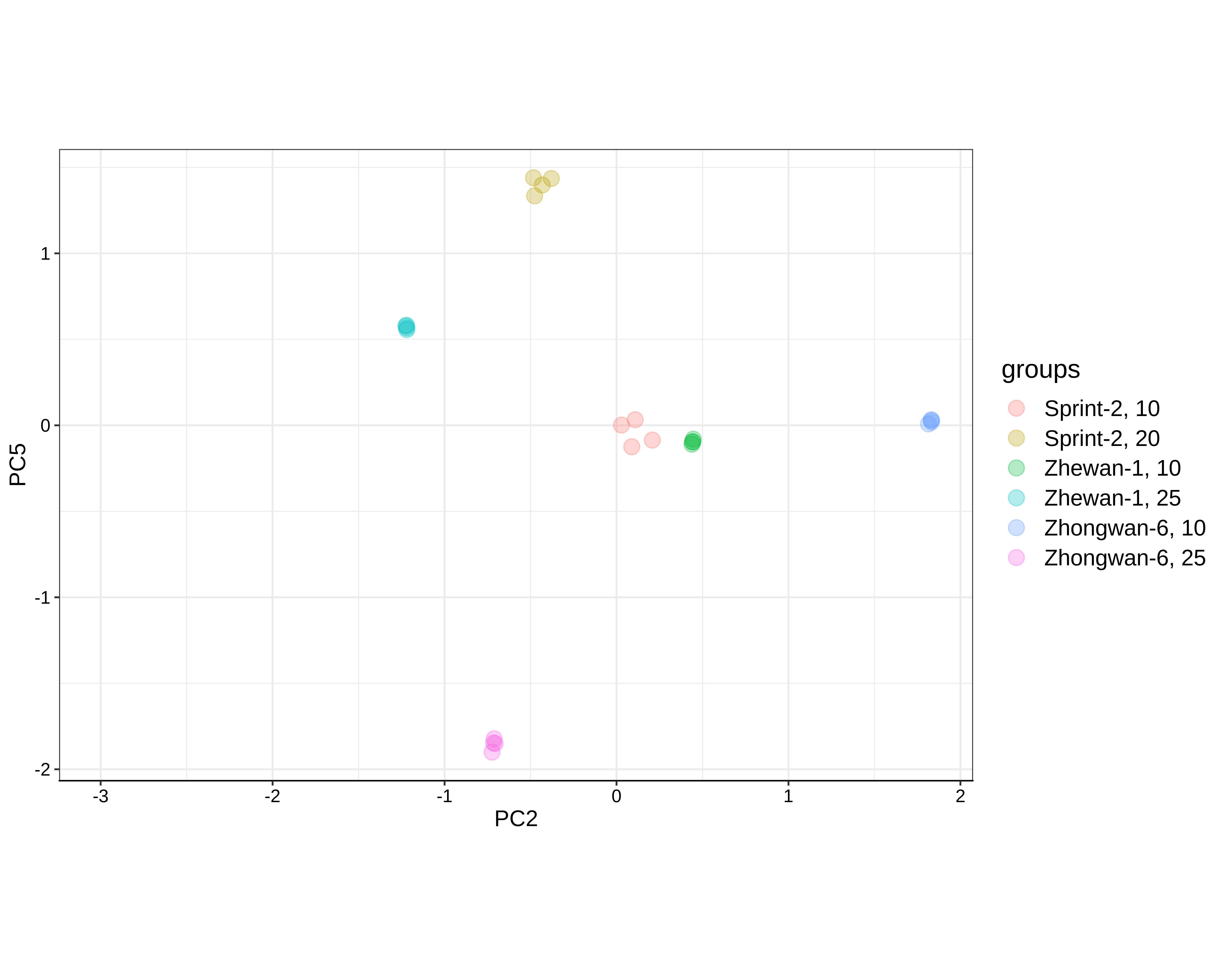

Supplement: Supplementary file 1 [file cells-09-00779-s001.zip › Supplementary materials/FigS9/7.tiff]

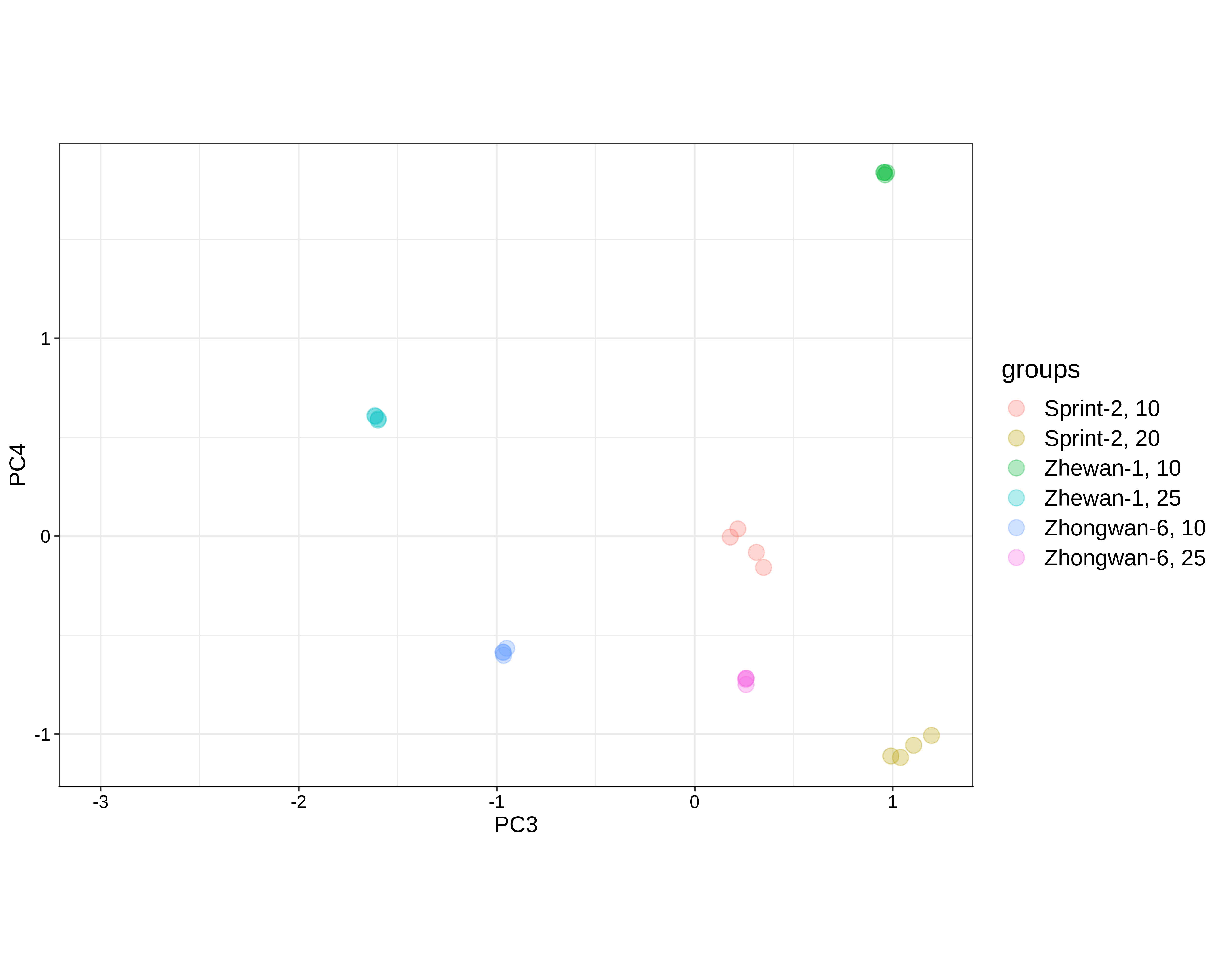

Supplement: Supplementary file 1 [file cells-09-00779-s001.zip › Supplementary materials/FigS9/8.tiff]

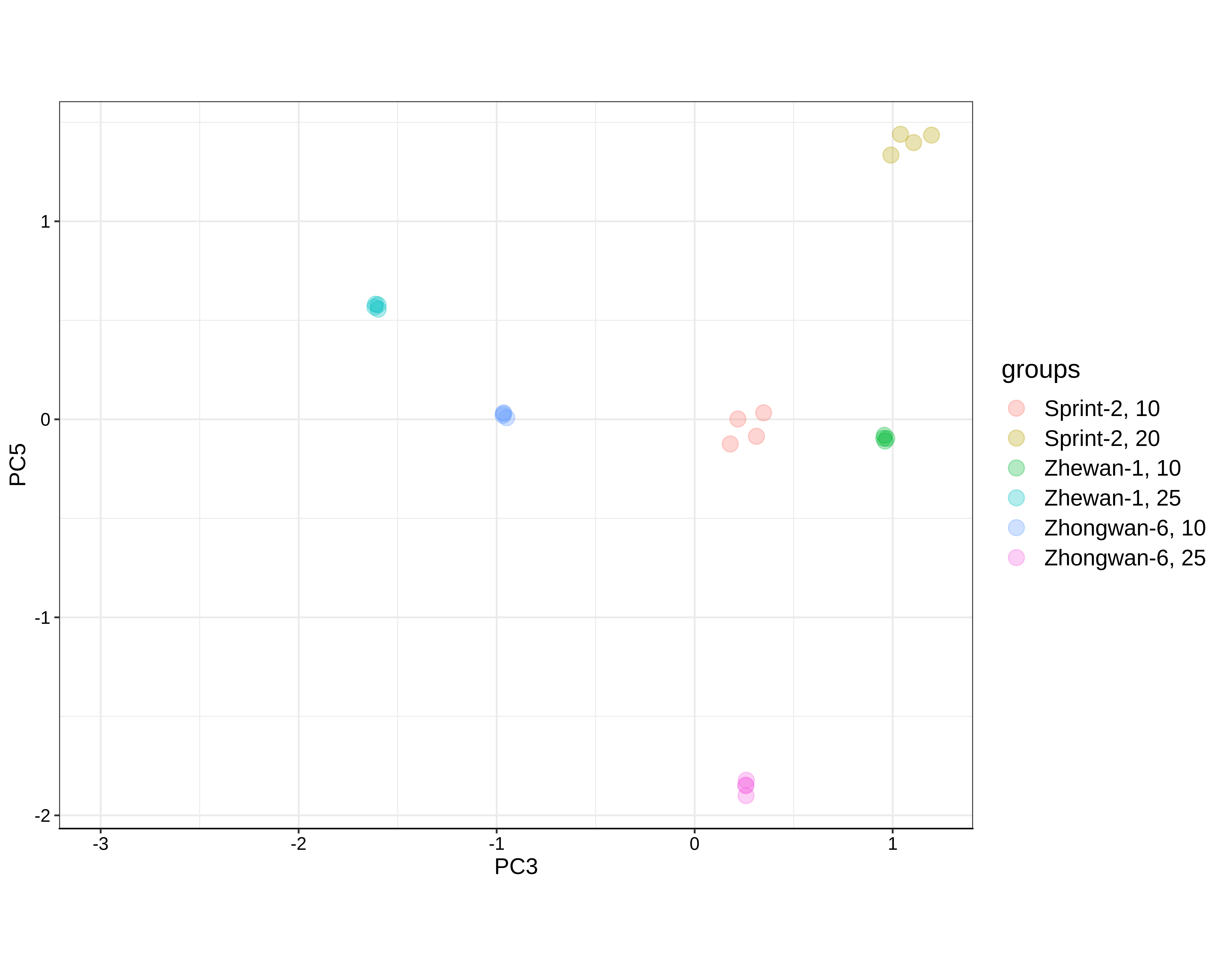

Supplement: Supplementary file 1 [file cells-09-00779-s001.zip › Supplementary materials/FigS9/9.tiff]
